# Transcriptomic and genetic studies identify *NFAT5* as a candidate gene for cocaine dependence

**DOI:** 10.1038/tp.2015.158

**Published:** 2015-10-27

**Authors:** N Fernàndez-Castillo, J Cabana-Domínguez, J Soriano, C Sànchez-Mora, C Roncero, L Grau-López, E Ros-Cucurull, C Daigre, M M J van Donkelaar, B Franke, M Casas, M Ribasés, B Cormand

**Affiliations:** 1 Departament de Genètica, Facultat de Biologia, Universitat de Barcelona, Barcelona, Catalonia, Spain; 2 Centro de Investigación Biomédica en Red de Enfermedades Raras (CIBERER), Barcelona, Spain; 3 Institut de Biomedicina de la Universitat de Barcelona, Barcelona, Catalonia, Spain; 4 Departament d'Estructura i Constituents de la Matèria, Universitat de Barcelona, Barcelona, Spain; 5 Department of Psychiatry, Hospital Universitari Vall d'Hebron, Barcelona, Spain; 6 Psychiatric Genetics Unit, Hospital Universitari Vall d'Hebron, Universitat Autònoma de Barcelona, Barcelona, Spain; 7 Biomedical Network Research Center on Mental Health (CIBERSAM), Barcelona, Spain; 8 Addiction and Dual Diagnosis Unit, Psychiatric Service, Hospital Universitari Vall d'Hebron, Agència de Salut Pública, Barcelona, Spain; 9 Department of Psychiatry and Legal Medicine, Universitat Autònoma de Barcelona, Barcelona, Spain; 10 Department of Human Genetics, Radboud University Medical Center, Nijmegen, The Netherlands; 11 Donders Institute for Brain, Cognition and Behaviour, Raboud University, Nijmegen, The Netherlands; 12 Department of Psychiatry, Radboud University Medical Center, Nijmegen, The Netherlands

## Abstract

Cocaine reward and reinforcing effects are mediated mainly by dopaminergic neurotransmission. In this study, we aimed at evaluating gene expression changes induced by acute cocaine exposure on SH-SY5Y-differentiated cells, which have been widely used as a dopaminergic neuronal model. Expression changes and a concomitant increase in neuronal activity were observed after a 5 μM cocaine exposure, whereas no changes in gene expression or in neuronal activity took place at 1 μM cocaine. Changes in gene expression were identified in a total of 756 genes, mainly related to regulation of transcription and gene expression, cell cycle, adhesion and cell projection, as well as mitogen-activeated protein kinase (MAPK), CREB, neurotrophin and neuregulin signaling pathways. Some genes displaying altered expression were subsequently targeted with predicted functional single-nucleotide polymorphisms (SNPs) in a case–control association study in a sample of 806 cocaine-dependent patients and 817 controls. This study highlighted associations between cocaine dependence and five SNPs predicted to alter microRNA binding at the 3′-untranslated region of the *NFAT5* gene. The association of SNP rs1437134 with cocaine dependence survived the Bonferroni correction for multiple testing. A functional effect was confirmed for this variant by a luciferase reporter assay, with lower expression observed for the rs1437134G allele, which was more pronounced in the presence of hsa-miR-509. However, brain volumes in regions of relevance to addiction, as assessed with magnetic resonance imaging, did not correlate with *NFAT5* variation. These results suggest that the *NFAT5* gene, which is upregulated a few hours after cocaine exposure, may be involved in the genetic predisposition to cocaine dependence.

## Introduction

Cocaine is a psychostimulant drug of abuse and its use has become a public health problem worldwide. Cocaine's pleasurable and addictive effects are thought to be mediated mainly through dopamine (DA), which is a key neurotransmitter in reward pathways.^1^ Cocaine binds the DA transporter producing an increase in DA concentration at the synapses and thus stimulating neurons in brain regions involved in reward and reinforcement behavior.^1, 2, 3^

Cocaine's chronic and acute effects on gene expression have been studied using a broad range of animal models and experimental paradigms and procedures, including human post-mortem samples.^4, 5^ These studies have identified gene expression changes in the brain related to diverse functional categories including synaptic communication and neuroplasticity, receptors, ion channels and transporters, cytoskeleton, extracellular matrix, oligodentrocytes and myelin, mitochondrial function, apoptosis and cell death, transcription factors and signal transduction. Moreover, two important pathways have been found affected by changes in gene expression: the mitogen-activated protein kinase (MAPK) and the synaptic long-term potentiation signal transduction pathways.^4, 5^

The repeated use of cocaine induces molecular and cellular adaptations in the central nervous system, such as synaptic changes and neuronal remodeling, and as the consumption becomes chronic those adaptations become stable.^6^ Individual's genetic background and environment determine the initial sensitivity to first drug exposure and how individual nerve cells and circuits adapt to chronic drug exposure, which could establish the development of addiction in some individuals but not others.^7^ Around 15–16% of cocaine users develop dependence, and heritability for cocaine addiction has been estimated around 60–70%.^8, 9, 10^ Some of those genetic factors may lie in genes that mediate acute and chronic cocaine's effects, conferring initial vulnerability to the establishment of drug-induced adaptations.

Compared with other drugs of abuse, relatively few association studies have been performed on cocaine dependence, and little is known about the genetic susceptibility to this psychiatric disorder.^11^ Some association studies have focused on candidate genes, especially on DA-related genes, the majority failing to detect associations or showing controversial results. Only associations with two genes, *CNR1* (cannabinoid receptor 1, brain) and *CHRNA5* (cholinergic receptor, nicotinic, alpha 5, neuronal) have been replicated so far.^11, 12, 13^ Other studies have assessed hundreds of single-nucleotide polymorphisms (SNPs) in multiple genes within candidate systems, and two genome-wide association studies have been reported in cocaine dependence, identifying shared as well as specific associations in European Americans and in African American populations.^14, 15, 16, 17^

We aimed at discovering novel genes involved in the susceptibility to cocaine dependence that could mediate its effects. Under the hypothesis that sequence variants in genes showing differential expression induced by cocaine may contribute to cocaine dependence, and considering the essential role that DA has in cocaine's effects and addiction, we designed a two-stage study by (i) identifying cocaine-induced changes in gene expression in a dopaminergic neuron-like model (SH-SY5Y) using microarray technology and (ii) subsequently considering differentially expressed genes as potential candidates for cocaine dependence, by assessing predicted functional SNPs in these genes through a case–control association study.

## Materials and methods

A brief description of the materials and methods is presented below. For detailed information of all procedures see Supplementary Information.

### Cell culture and cocaine treatments

SH-SY5Y cells (ATCC, LGC Standards, Middlesex, UK) were differentiated with retinoic acid (Sigma-Aldrich Corporate, St. Louis, MO, USA) during 7 days at a final concentration of 10 μM supplemented on the media (50:50 Dulbecco's modified Eagle's medium:F12, 10% fetal bovine serum and 1% P/S, Gibco, Life Technologies, Carlsbad, CA, USA). SH-SY5Y differentiation was assessed by changes compatible with neuron-like morphology and neurite outgrowth, expression of tyrosine hydroxylase as a dopaminergic neuronal marker by western blot, and cell cycle analysis (Supplementary Figure 1). Cytotoxicity of cocaine was assessed with XTT assays (Cell Proliferation Kit II, Roche Life Sciences, Branford, CT, USA) at 0, 1, 5, 10, 15 and 20 μM of cocaine–HCl. The range of cocaine concentrations were selected based on concentrations determined in human plasma and brain in different studies.^1, 18, 19, 20, 21, 22, 23, 24^ For gene expression analysis, cocaine treatment was performed on differentiated SH-SY5Y cells at 0, 1 and 5 μM, selected on the basis of a previous microarray study^25^ and the range observed in cocaine abusers.^19, 21^ After 30 min of exposure, the medium was replaced and cells were retrieved at 6 or 24 h.

### Microarray and qRT-PCR experiments

RNA was isolated from nine dishes per condition (RNeasy Mini Kit, Qiagen, Hilden, Germany) and pools of three dishes were hybridized to the GeneChip Human Genome U133 Plus 2.0 Array (Affymetrix, Santa Clara, CA, USA). Microarray data have been deposited in NCBI's Gene Expression Omnibus (GEO) and are accessible through GEO Series accession number GSE71939 (http://www.ncbi.nlm.nih.gov/geo/query/acc.cgi?acc=GSE71939).

For quantitative real-time (qRT-PCR) validation, we initially selected genes showing differences in expression ⩾1.5-fold, a total of 143. From those we considered genes included in representative enriched functional categories, pathways or gene networks. Finally, we selected eight genes based on their functions and possible involvement in mediating cocaine effects and neuroadaptations, in which expression had not previously been found to be altered by cocaine. Selected genes were validated using qRT-PCR and further assessed in new experiments at different time points (2, 4, 5, 6, 7, 8 and 10 h) after a cocaine acute exposure (0 vs 5 μM) with three replicates per condition. We performed a relative quantification of the results of the qRT-PCR experiments using glyceraldehyde-3-phosphate dehydrogenase and hypoxanthine phosphoribosyltransferase (*HPRT1*) expression for normalization.

### Calcium imaging and neuronal activity monitoring

We used calcium imaging (Fluo-4-AM) to monitor changes in neuronal activity in differentiated SH-SY5Y cells at 0, 1, 5 and 10 μM cocaine–HCl exposure as previously described.^26^ Recordings were performed during 15  min per condition, assessing over eight replicates (160–240 active neurons per condition).

### Subjects

Seven genes showing differential expression profiles after an acute cocaine treatment were selected to perform a case–control association study on cocaine dependence. The clinical sample included 806 cocaine-dependent subjects according to DSM-IV TR criteria (Diagnostic and Statistical Manual of Mental Disorders, 4th edn, text revision) seeking treatment in the 'Addiction and Dual Diagnosis Unit' of Vall d'Hebron Hospital (Barcelona, Spain) and 817 sex-matched healthy controls (see Supplementary Table 1 for details). The controls were recruited at the Blood and Tissues Bank of Vall d'Hebron Hospital; none of them had injected drugs intravenously. All individuals were Spanish and Caucasian, with the two last names (one from each parent) of Spanish origin. All of them signed the informed consent, previously approved by the Ethics Committee and were evaluated according to the 'Addiction and Dual Diagnosis Unit' protocol.^27^ DNA samples were isolated from peripheral blood. Population stratification was previously discarded in our sample.^15^

### SNP selection and genotyping

A total of 22 SNPs within seven candidate genes were selected based on their predicted functional effect using the FuncPred software (http://snpinfo.niehs.nih.gov/snpinfo/snpfunc.htm) and two additional SNPs at the *SEMA6D* gene associated with substance dependence in a previous genome-wide association study^28^ were also included in the assay. Finally, 23 SNPs were successfully genotyped with KASP technology with an average genotype call rate of 98.2%, and further evaluated in a case–control association study.

### Functional evaluation of SNP effects on microRNA regulation using a luciferase assay

The effect of SNPs showing consistent predictions on microRNA regulation (using different software tools, see Supplementary Information) was experimentally tested by a luciferase reporter system in HeLa and in SH-SY5Y cells. The 3′-untranslated regions containing both alleles of each SNP were cloned in the pmirGLO Dual-Luciferase miRNA Target Expression Vector (Promega, Madison, WI, USA) and cotransfected into HeLa and SH-SY5Y cells with the corresponding microRNA cloned in a pCMV-MIR vector (OriGene, Rockville, MD, USA). Luciferase expression was assessed using the Dual-luciferase Reporter Assay System (Promega).

### Neuroimaging genetics studies

The effect of *NFAT5* SNPs on regional brain volumes was tested using neuroimaging and genetic data of 1300 self-reported healthy adults from the Dutch Cognomics Resource Brain Imaging Genetics (http://www.cognomics.nl).^29^ Seven regions of interest known to be involved in drug addiction^30^ (orbitofrontal cortex, prefrontal cortex, nucleus accumbens, putamen, caudate nucleus, hippocampus and insula) were assessed in the discovery sample (*n*=645, scanned at 1.5 Tesla) and then in the replication sample (*n*=655, scanned at 3 Tesla; Supplementary Table 2).

### Statistical analyses

We used the *affy* library to perform background correction, normalization and summarization, considering the background method, the Robust Multichip Average method and the median polish method, respectively.^31^ Genes were filtered by signal (threshold log2(40)), and the expression profiles were compared using the limma library. We used DAVID Annotation Tool^32^ for the functional enrichment clustering and the Ingenuity Pathway Analysis v8.8 software (http://www.ingenuity.com/products/ipa) for gene network and canonical pathway enrichment analyses. WebGESTALT was used for microRNA-binding site enrichment analyses considering upregulated and downregulated subsets of genes separately.^33^

The minimal statistical power in the case–control association study was estimated *post hoc* considering the SNP with the lowest minimum allele frequency (MAF = 0.143) and assuming an additive model of inheritance, with an estimated statistical power of 98% using the software Power Calculator for Genetic Studies (http://sph.umich.edu/csg/abecasis/CaTS). Analysis of Hardy–Weinberg equilibrium and the comparison of genotype frequencies between cases and controls under an additive model were performed using the *SNPassoc* R package.^34^ Significant *P*-values were adjusted for age. Bonferroni correction for multiple testing was applied considering 22 independent tests (*P*<0.0022).

For cytotoxicity, qRT-PCR, calcium imaging and luciferase experiments, differences between conditions were evaluated with the IBM SPSS Statistics Software Version 22.0 (Released 2013; IBM, Armonk, NY, USA) using a Mann–Whitney non-parametric *U*-test, as normality was rejected using the Kolmogorov–Smirnov test (as expected, given the small number of samples), and statistical significance was set at *P*<0.05. Brain volume analyses were performed by linear regression using PLINK software (http://pngu.mgh.harvard.edu/~purcell/plink/).

## Results

Under the hypothesis that cocaine-induced gene expression changes may highlight novel candidate genes predisposing to cocaine dependence, we performed an *in vitro* study in a dopaminergic neuron-like model to assess transcriptional changes induced by cocaine. Subsequently, we tested those genes showing differential expression as potential candidates for cocaine dependence through a case–control association study.

### Cocaine-induced changes in gene expression in SH-SY5Y cells differentiated to dopaminergic neurons

**Figure 1:**
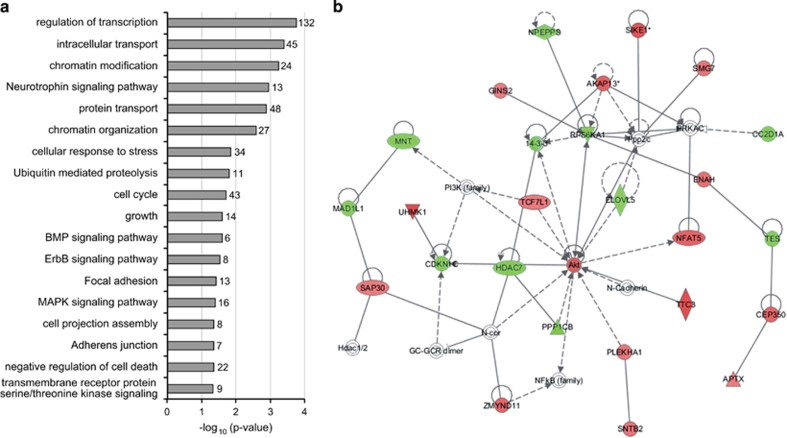
Gene expression changes caused by exposure to 5 μM cocaine *in vitro* after 6 h. (**a**) Representative over-represented biological categories (Gene Ontology terms, GO) and pathways (Kyoto Encyclopedia of Genes and Genomes, KEGG) identified by DAVID software among the differentially expressed genes. The number of genes with altered expression included in each category is indicated on the right side of the bar. (**b**) Gene network involved in molecular transport, cell-to-cell signaling and interaction and cellular development (score=34). The green and red nodes in the pathway indicate the down- and upregulated genes, respectively, induced by 5 μM cocaine–HCl after 6 h.

**Figure 2:**
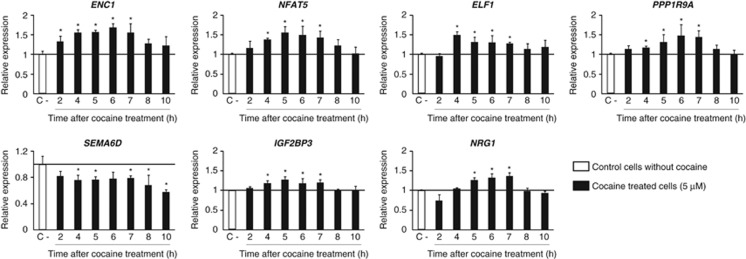
Quantitative real-time PCR validation of gene expression changes identified by microarray experiments. Transcription levels of seven genes involved in neuronal adaptations and transcription were determined by qRT-PCR at different time points after a 30-min exposure to 5 μM cocaine. Significant differences compared with control cells (not exposed to cocaine) normalized to *GAPDH* are indicated (*P<0.05). Error bars indicate s.d.

SH-SY5Y cells differentiated to dopaminergic neurons (Supplementary Figure 1) showed no cocaine cytotoxic effects at any of the conditions under study (data not shown), and gene expression experiments were conducted at 6 or 24 h after 30  min of cocaine treatment at 0, 1 and 5 μM. After 6 h of an acute 30-min exposure to 5 μM cocaine, 756 genes exhibited significantly altered expression levels when compared to untreated cells (419 upregulated and 337 downregulated; Supplementary Table 3). Analysis of functional group over-representation identified several processes, including regulation of transcription, chromatin modification, focal adhesion and cell projection, and also neurotrophin and MAPK signaling pathways, among others (Figure 1a). Gene network construction showed a highly scored network (score=34, Figure 1b) involved in molecular transport, cellular development and cell-to-cell signaling and interaction. The canonical pathways 'neuregulin signaling' and 'cyclic AMP response element-binding protein (CREB) signaling in neurons' were also altered (Supplementary Figure 2). The analysis of enrichment of microRNA-binding sites identified miR-124a, with predicted targets in 22 genes upregulated by cocaine (see Supplementary Table 4). The validation assays of expression patterns at different time points for eight genes showing expression differences ⩾1.5-fold and involved in neuroadaptation, axon guidance, neuroplasticity, neurite outgrowth, neurotrophin signaling pathway or transcription regulation, confirmed increased expression around 6 h after cocaine exposure for ectodermal–neural cortex 1 (*ENC1*), nuclear factor-activated T-cells 5 (*NFAT5*), E74-like factor 1 (*ELF1*), protein phosphatase 1 regulatory subunit 9A (*PPP1R9A*), insulin-like growth factor 2 mRNA-binding protein 3 (*IGF2BP3*) and neuregulin 1 (*NRG1*), and decreased expression for semaphorin 6D (*SEMA6D*) (Figure 2).

**Figure 3:**
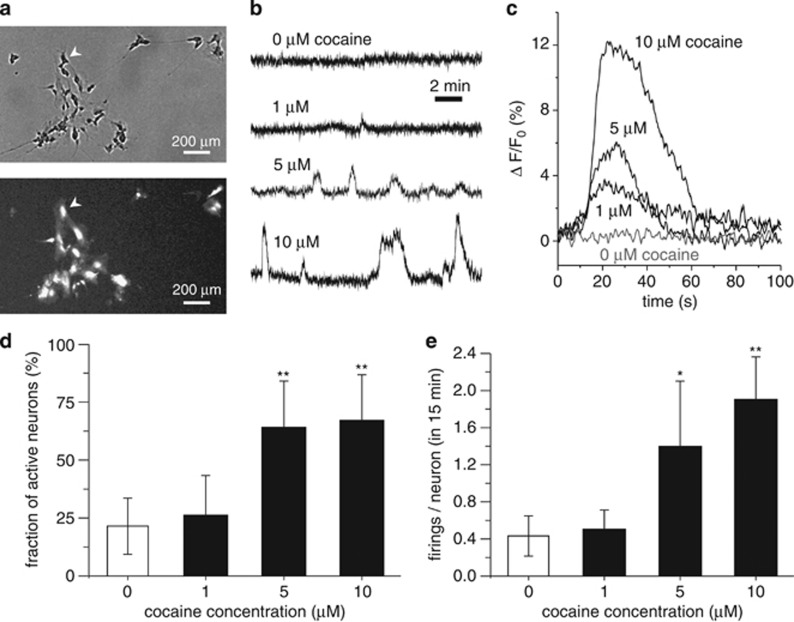
Neuronal activity changes induced by cocaine. (**a**) Comparison of a phase-contrast snapshot (top) with the fluorescence equivalent (bottom). For the latter, bright spots are firing neurons, showing the ability to track neuronal activity with single-cell resolution. The arrowhead indicates the same neuron in both images. (**b**) Representative fluorescence traces of neuronal activity for gradual exposure to higher cocaine concentrations. Neuronal response is weak for 1 μM concentration, and increases progressively for 5 and 10 μM. (**c**) Detail of neuronal response to cocaine, showing that the firing amplitude increases with cocaine concentration. Each trace at a given concentration is an average over the responses of 10 different neurons. (**d**) Comparison of the fraction of active neurons, showing that network activity significantly increases for 5 and 10 μM cocaine exposure compared to the 0 μM (spontaneous acitivity) and 1 μM concentration. (**e**) Average number of activations per neuron for the different conditions, highlighting the much higher neuronal activity at 5 and 10 μM cocaine. Significant differences compared to 0 μM cocaine are indicated. *P<0.05 and **P<0.01. Error bars indicate s.d.

No differences in gene expression were observed in the microarray experiments when cells were treated with 1 μM cocaine or 24 h after exposure. In order to explain the lack of changes observed at this concentration, we hypothesized that neuronal activation may co-occur with transcriptional changes above a specific threshold of cocaine concentration, and 1 μM cocaine may not be sufficient to induce detectable changes in neuronal activity nor in gene expression. For this purpose, we investigated neuronal network activity by means of calcium imaging after exposure to different cocaine concentrations (Figure 3a). We observed a concentration-dependent progressive increase in neuronal response and firing amplitude (Figures 3b and c). No changes in the percentage of active neurons were observed when we compared 0 and 1 μM cocaine treatment (22 and 26%, *P*=0.44), nor in the average number of firing/neuron (0.4 and 0.5 firings/neuron, *P*=0.40) (Figures 3d and e). Increases in active neurons and a higher number of firings/neuron, however, were detected after exposure to 5 μM (64%, *P*=8.7e−05; 1.4 firings/neuron, *P*=0.047) or 10 μM cocaine–HCl (67%, *P*=1.5e−06; 1.9 firings/neuron, *P*=3.6e−03) compared to 0 μM (Figures 3d and e). The absence of differences in neuronal activity below 1 μM correlates with the lack of differences observed in gene expression at this cocaine concentration.

### Case–control association study on cocaine dependence

The seven genes that showed cocaine-induced expression changes validated by qRT-PCR were subsequently considered as candidates to contribute to cocaine dependence susceptibility. Twenty-three potentially functional SNPs in genes showing cocaine-induced changes in expression levels (*NFAT5*, *ELF1*, *PPP1R9A*, *SEMA6D* and *IGF2BP3*) were subsequently followed-up in a case–control association study of 806 cocaine-dependent patients and 817 sex-matched healthy controls. All SNPs, except for rs854524, not considered in the subsequent analyses, were in Hardy–Weinberg equilibrium both in cases and in controls (Supplementary Table 5).

**Figure 4:**
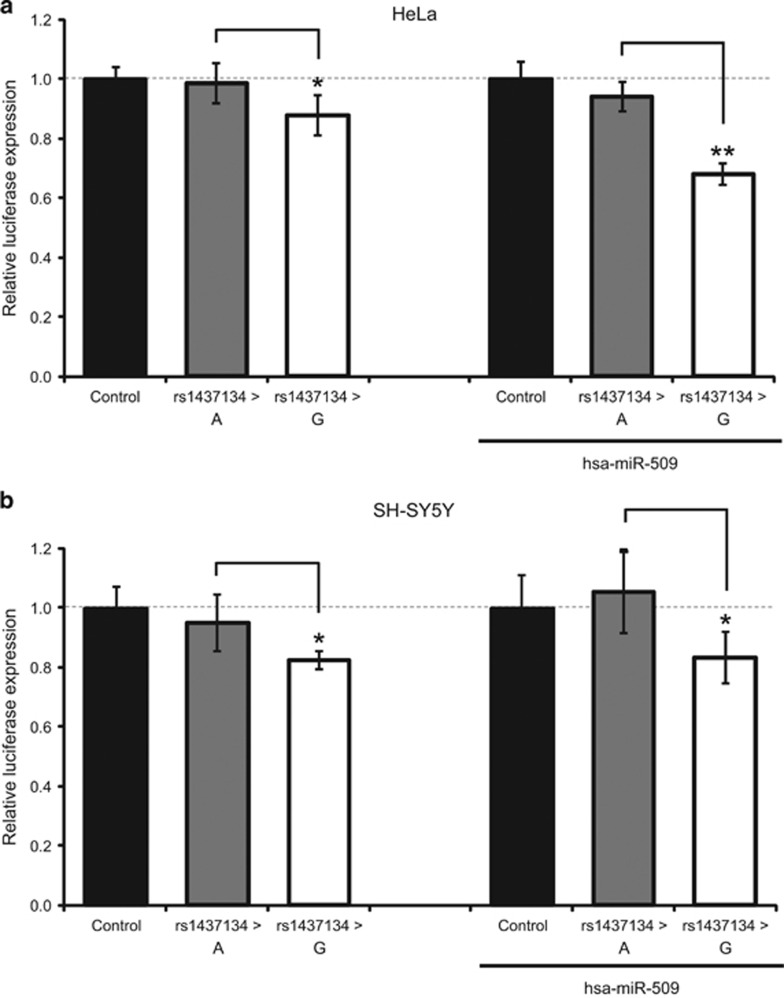
Effect of rs1437134 on gene expression. Effect on gene expression of the two alleles of the associated variant rs1437134 in absence and presence of hsa-miR-509 in HeLa cells (**a**) and in SH-SY5Y cells (**b**). Relative luciferase expression of the pmirGlo vector (control) and the constructs with the 3′-untranslated region of *NFAT5* containing the rs1437134 A and G alleles. Significant differences between the G and the A allele are indicated as *P<0.05 and **P<0.01. Error bars indicate s.d.

The single-marker analysis showed that five SNPs in the 3′-untranslated region of the *NFAT5* gene were associated with cocaine dependence, and two of them (rs1437134 and rs7359336, in high linkage disequilibrium) survived the Bonferroni correction for multiple testing (Table 1; Supplementary Table 5; Supplementary Figure 3). All five variants were predicted to alter binding sites for microRNAs (Supplementary Figure 4) and two of them, rs1437134 and rs11641233, were predicted to alter hsa-miR-509 and hsa-miR-649 binding to the *NFAT5* messenger RNA by at least three different software tools. We subsequently focused on these two SNPs and performed a luciferase reporter assay in HeLa and SH-SY5Y cells. A significant decrease in gene expression was observed for allele rs1437134G compared to allele rs1437134A, both in HeLa and in SH-SY5Y cells (9 and 13%, respectively; Figure 4). In the presence of the microRNA hsa-miR-509, the decrease in gene expression shown by rs1437134G, compared to rs1437134A, was more pronounced in both cell lines (31% in HeLa and 21% in SH-SY5Y; Figure 4). In contrast, no effect on gene expression was detected for rs11641233 in the presence of hsa-miR-649 (data not shown).

Finally, as NFAT5 is a member of the NFAT protein family, involved in axon guidance, and it is highly expressed in the developing and adult brain, we tested possible effects of *NFAT5* variation on brain volumes using brain imaging data. In this way, we aimed at identifying potential mechanisms mediating the effect of this gene on addiction risk. However, no significant correlations were observed between brain volumes in the regions of interest and any of the SNPs investigated (Supplementary Table 6).

## Discussion

This study aimed at uncovering genes mediating cocaine's effects in an *in vitro* model that could eventually participate also in the susceptibility to cocaine dependence. For this purpose, we first identified genes showing differential expression under cocaine exposure in a dopaminergic cell model, and we subsequently investigated their possible role in the predisposition to cocaine dependence by assessing functional common genetic variants through a case–control study. The results of our experimental design pointed at *NFAT5*, which is upregulated by cocaine and bears functional risk variants for cocaine dependence.

To our knowledge, this is the first study assessing gene expression changes induced by cocaine in a dopaminergic cell model, with dopaminergic neurons being the key of the reward system and cocaine pleasurable effects. For this purpose, we used the SH-SY5Y cell line differentiated with retinoic acid, which shows neuron-like morphology, has increased DA content, expresses neuronal and dopaminergic markers, has functional DA transporter, and features excitability, potential propagation and enhanced dopaminergic neurotransmission.^35, 36, 37, 38^

*In vitro* studies assessing the effect of cocaine exposure on gene expression have previously been performed in neuronal progenitor or fetal cells to study prenatal brain alterations, and showed changes in immune and inflammatory responses, and cell-death related genes.^25, 39, 40^ Under our experimental conditions, changes in expression were detected for genes involved in transcription, transport, cell cycle, cell projection and adhesion, and MAPK and CREB signaling, which is in agreement with previous gene expression studies after cocaine abuse performed in humans and animals.^4, 5^ Several genes showing differential expression in our study were also found altered in previous studies performed in human post-mortem samples and rat models, including *ADORA1* (adenosine A1 receptor), *CALM2* (calmodulin 2), *GRIN1* (glutamate receptor, ionotropic, *N*-methyl d-aspartate 1), *LAMB1* (laminin beta 1) and *SMN1* (survival of motor neuron 1, telomeric; see Supplementary Table 3).^41, 42, 43, 44, 45, 46^ We detected the effect of cocaine exposure on SH-SY5Y cells at 6 h after 5 μM cocaine treatment. This is different from a previous study by Crawford *et al.*^25^ in human neuronal progenitor cells showing gene expression changes 24 and 48 h after only 1 μM cocaine treatment. The results of the transcription analysis correlated with our calcium imaging experiments, a technique previously employed in SH-SY5Y cells to assess caffeine and carbachol effects,^47^ that revealed significant increases in neuronal activity after exposure to 5 μM cocaine, but almost no activity changes under 1 μM cocaine. This is consistent with previous studies detecting cocaine concentrations in the caudate-putamen of cocaine abusers in the range of 0.8–1.8 μM (average about 1 μM) during the 30  min after an intravenous cocaine dose of 0.1 mg kg^−1^,^48^ a dose that was not sufficient to produce a subjective ‘high', rush and craving in humans;^1^ these were only observed at higher intravenous doses (0.3 and 0.6 mg kg^−1^, commonly used by cocaine abusers). Thus, 1 μM cocaine in dopaminergic regions of the brain (after a 0.1-mg kg^−1^ dose) would not be enough to produce those cocaine effects, which may correlate with our findings obtained in neuronal activity and expression studies *in vitro*.

Among the genes showing the most pronounced expression changes after exposure to 5 μM cocaine, we selected some showing functions that could potentially mediate cocaine effects and participate in neuronal circuit remodeling and neuroadaptations that lead to cocaine dependence. We succeeded in validating expression changes for seven of the eight selected genes, *ENC1*, *NFAT5*, *ELF1*, *PPP1R9A*, *SEMA6D*, *IGF2BP3* and *NRG1*, whose expression had not previously been described to be altered after exposure to cocaine*. ENC1* (NRP/B) is primarily expressed in neurons and encodes an actin-binding protein that induces neurite outgrowth and has a role in nervous system development and differentiation.^49, 50^ *PPP1R9A* is also expressed in neurons; it encodes Neurabin-1, a synaptic protein that controls neuronal actin cytoskeleton and reorganization and is involved in neurite formation.^51, 52^ Semaphorin 6D, encoded by *SEMA6D*, is involved in axon guidance, and SNPs in this gene have previously been associated with substance dependence in a genome-wide association study.^28, 53, 54^ Other semaphorins have also been found upregulated by cocaine in animal and human studies.^44, 55^ *IG2BP3* is involved in neuronal differentiation and Neuregulin1, encoded by *NRG1*, is a signaling protein that mediates cell-to-cell interactions, neuronal survival, synaptic maturation and maintenance, growth cone dynamics and trafficking of neurotransmitter receptors.^56, 57^ Both *ELF-1* and *NFAT5* encode transcription factors that have been studied mainly in lymphoid cells and immune response. ELF-1 binds to the EBS elements of NFAT1, another NFAT family member.^58^

We subsequently considered these seven genes, which could mediate cocaine's effects and neuroadaptations, as candidates for participating in the susceptibility to cocaine dependence. Functional SNPs in these genes, selected as potential risk factors for this phenotype, were assessed in our Spanish sample of cocaine-dependent patients and controls. Our case–control association study with common genetic variants pointed to five SNPs in *NFAT5* as risk factors for cocaine dependence, with rs1437134 surviving the Bonferroni correction for multiple testing and showing evidence of functional effects on gene expression. The risk allele for cocaine dependence, rs1437134G, determined a decreased *NFAT5* expression, an effect that was more pronounced in the presence of the microRNA hsa-miR-509 in the two cell lines tested. Previous studies support a link between the effects of cocaine and microRNAs. Cocaine chronic exposure resulted in increased Ago2 messenger RNA and protein in the striatum (a key brain region involved in addiction), and consequently an alteration of microRNA expression levels.^59^ Also, cocaine administration induced expression changes in a wide range of microRNAs in dopaminergic neurons in the striatum.^60^ A subset of these microRNAs upregulate genes known to influence the motivational properties of cocaine in mice, such as *Bdnf*, *FosB* and *Cdk5r1*.^60^ Additional evidence suggesting that cocaine may exert its effects on gene expression through the regulation of the microRNA machinery was also found in our study, as an enrichment of predicted binding sites for miR-124a was observed among the list of genes found upregulated by cocaine. Interestingly, miR-124a has previously been reported to be downregulated in SH-SY5Y cells after exposure to cocaine and in the mesolimbic dopaminergic system after chronic cocaine administration.^61, 62^

Our expression and case–control association studies suggest that *NFAT5* may contribute to the vulnerability to cocaine dependence, which is in agreement with previous evidence suggesting that cocaine-induced activation of gene expression may be partially mediated by NFAT-dependent transcription.^63^ Transcription regulated by NFAT is shown to be induced by DA receptor stimulation. Cocaine triggers striatal NFAT4c nuclear translocation, possibly through a DA increase in the synaptic cleft produced by this drug.^63^ Interestingly, *NFAT5* was present in the gene network identified, and NFAT canonical pathways were significantly over-represented in our gene expression study (Figure 1b; Supplementary Figures 2 and 5), which means that several genes regulated by NFAT or encoding related proteins are also differentially expressed after cocaine exposure. The NFAT family is involved in axonal growth and guidance by calcineurin/NFAT signaling pathway. NFAT5 (also known as TonEBP), however, differs from the other family members, as it does not have the calcineurin-binding domain.^64, 65^ It has been involved in regulating response to osmotic stress and hypertonicity in several cell types, including T cells, kidney and neurons, and its activation also upregulates its own transcription.^64, 66, 67, 68, 69^ It is highly expressed in the brain at embryonic stages, but little is known about its function in the brain.^66, 70^ Interestingly, a recent study suggests that NFAT5 could participate in DA synthesis and secretion in renal proximal tubule cells.^71^ If NFAT5 is involved in DA neurotransmission in the brain, genetic variants within this gene may predispose to cocaine dependence through changes in DA activity. This would be in agreement with ‘the reward deficiency syndrome' hypothesis, which postulates that hypodopaminergic activity predisposes to cocaine addiction.^72^

Considering all these data, NFAT5, a transcription factor, could be an important mediator of cocaine's effects by activating NFAT-dependent transcription as well as dopaminergic activity. Cocaine might activate NFAT5 nuclear translocation, as it was shown for another member of the NFAT family,^63^ being responsible for cocaine-induced changes in gene expression, including its own upregulation. It is thus tempting to speculate that genetic variants impacting *NFAT5* will cause an effect on the expression of relevant downstream genes and on DA activity, which could eventually contribute to cocaine dependence phenotypes.

This study should be viewed in terms of several strengths and limitations. Some strengths are as follow: (i) through a comprehensive hypothesis-free study, we have identified variants in a gene that seem to have a functional impact and that may participate in cocaine dependence; (ii) gene expression changes detected with microarrays were validated and are consistent with other studies; (iii) the clinical sample was evaluated by members of the research team in a single hospital following the same clinical assessment; (iv) all individuals, cases and controls, were Spanish, Caucasian and from the same small geographical area in Barcelona, Spain. Some limitations of the study, however, should be recognized: (i) cocaine effects on gene expression and neuronal activity were performed in a dopaminergic neuron-like model, from a tumor cell line, and thus they may differ from those taking place in the brain; (ii) the limited number of replicas in the microarray study may have prevented us from identifying existing differences in gene expression; (iii) in the association study, SNPs in the candidate genes were not selected under genetic coverage criteria, and thus, other variants not tested by us may be involved in cocaine dependence predisposition; (iv) cocaine dependence was not discarded in the control sample, which could lead to false-negative findings in our association study; (v) the SNPs found associated were not assessed in a replication sample; (vi) the functional effect of SNPs on microRNA regulation was studied *in vitro* with a reporter system and overexpressing the microRNAs, and may differ considerably from real conditions; (vii) we had a large sample for the neuroimaging genetics study, but we only tested few brain phenotypes, based on earlier findings on regions of interest to addiction rather than testing brain-wide effects.

To sum up, our data indicate that cocaine-induced changes in gene expression occur in differentiated SH-SH5Y cells a few hours after exposure to the drug, which are related to regulation of transcription and gene expression, cellular movement and neuronal adaptations. These changes occur at 5 μM cocaine, a concentration that increases neuronal activity and firing. Additional evidence suggests that a common functional variant in one of the genes showing increased expression after cocaine exposure, rs1437134 in *NFAT5*, may contribute to cocaine dependence. However, further genetic and functional studies of *NFAT5* are needed to confirm its role in cocaine dependence.

## Figures and Tables

**Table 1:** SNPs associated with cocaine dependence in the *NFAT5* gene in a Spanish sample of 806 cocaine-dependent patients and 817 controls

						
				*Controls* N *(%)*				*Cases* N *(%)*						
*Locus*	*Marker*		*Predicted effect*	*11*	*12*	*22*	*Sum*	*11*	*12*	*22*	*Sum*	P*-value*^a^	*Adj* P*-value*^b^	*OR (95% CI)*
*NFAT5*	rs1437134	G>A	miRNA binding	278 (34.2)	384 (47.3)	150 (18.5)	812	325 (40.5)	370 (46.1)	108 (13.4)	803	0.00118^c^	0.00031^c^	1.27 (1.10–1.45)^d^
	rs7359336	A>G	miRNA binding	278 (34.1)	386 (47.3)	152 (18.6)	816	321 (40.2)	369 (46.2)	109 (13.6)	799	0.00141^c^	0.00035^c^	1.25 (1.09–1.45)^d^
	rs11641233	C>T	miRNA binding	516 (63.2)	264 (32.3)	37 (4.5)	817	461 (57.5)	286 (35.7)	55 (6.8)	802	0.00734	0.02599	1.25 (1.06–1.47)
	rs6499244	A>T	miRNA binding	264 (32.4)	390 (47.8)	161 (19.8)	815	293 (36.5)	385 (48.0)	124 (15.5)	802	0.0156	0.00474	1.37 (1.03–1.37)^d^
	rs12232410	G>A	miRNA binding	516 (63.3)	262 (32.2)	37 (4.5)	815	463 (57.5)	288 (35.8)	54 (6.7)	805	0.00749	0.0351	1.25 (1.06–1.47)

Abbreviations: CI, confidence interval; miRNA, microRNA; OR, odds ratio; SNP, single-nucleotide polymorphism.

a Log-additive model.

b Adjusted by age.

c Survive Bonferroni correction *P*<0.0022.

d When OR<1, the inverted score is shown.

## Supplementary Material

Supplementary Figure Legends

Supplementary Informations
