# Supplementary figures and images for: Transcriptomic and genetic studies identify NFAT5 as a candidate gene for cocaine dependence
Source: Transl Psychiatry. 2015 Oct 27;5(10):e667–. doi: 10.1038/tp.2015.158 (PMC4930134; doi:10.1038/tp.2015.158)

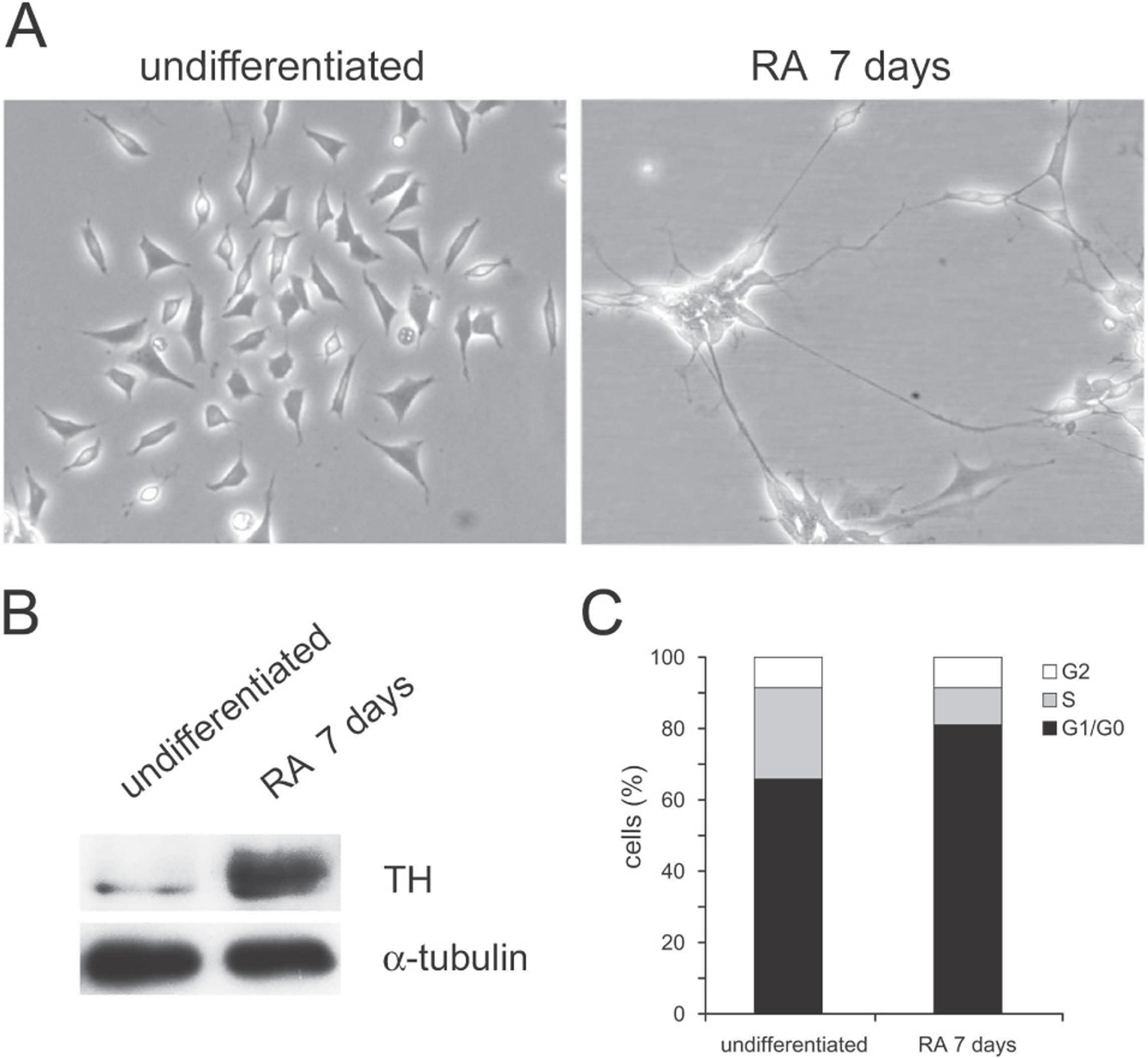

Supplement: Supplementary Figure 1 [file tp2015158x1.tif]

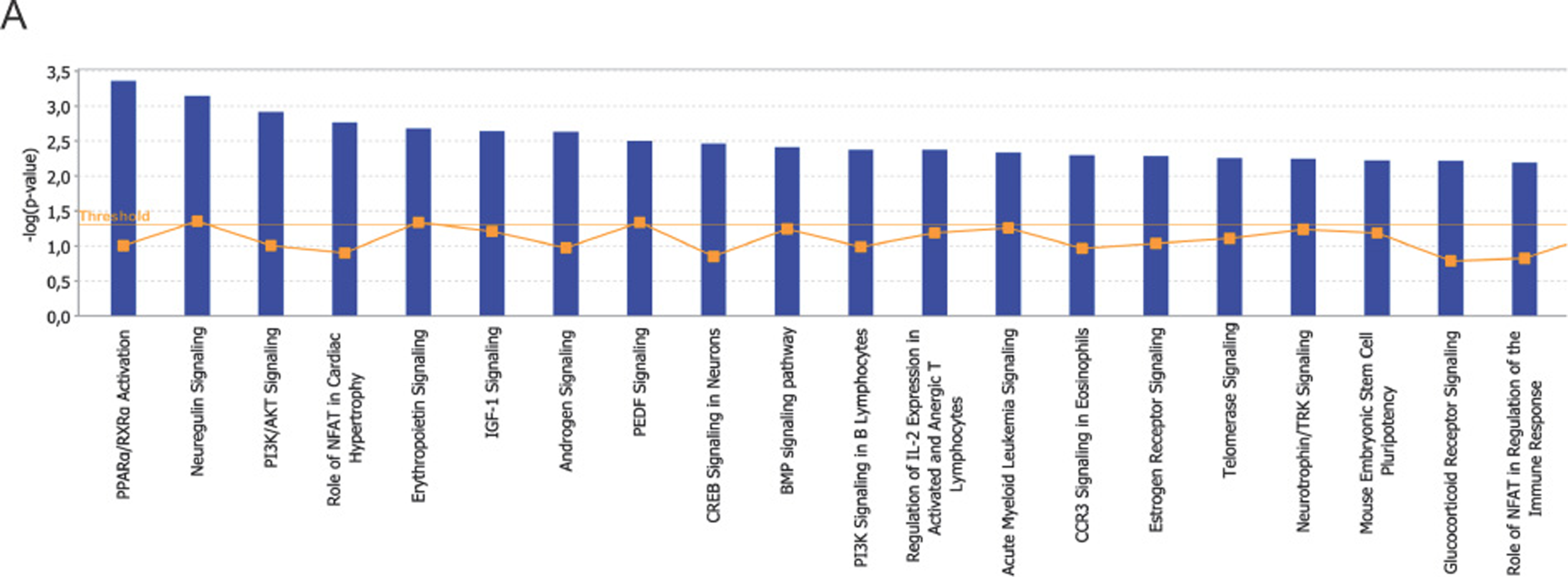

Supplement: Supplementary Figure 2 [file tp2015158x2.tif]

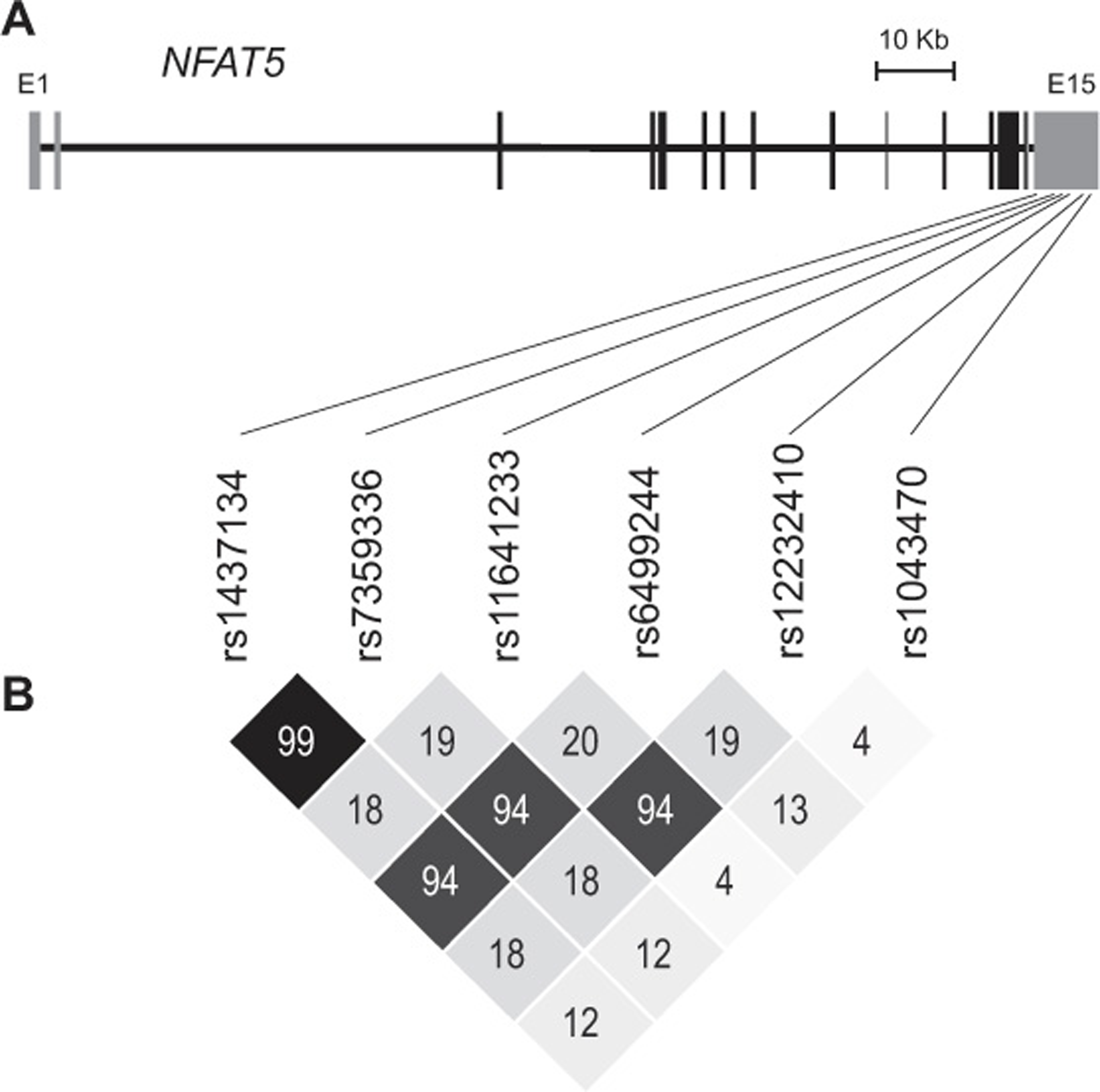

Supplement: Supplementary Figure 3 [file tp2015158x3.tif]

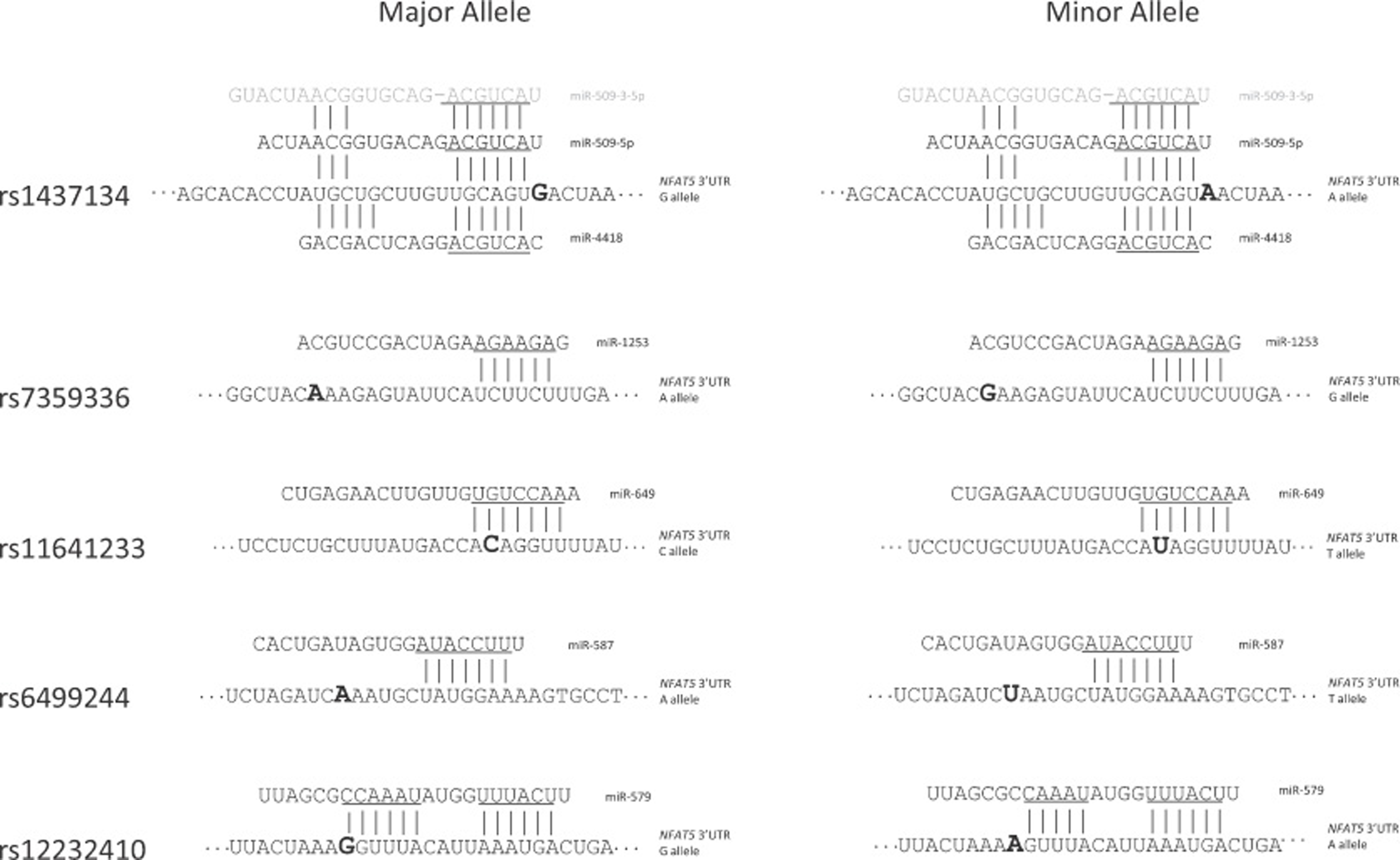

Supplement: Supplementary Figure 4 [file tp2015158x4.tif]

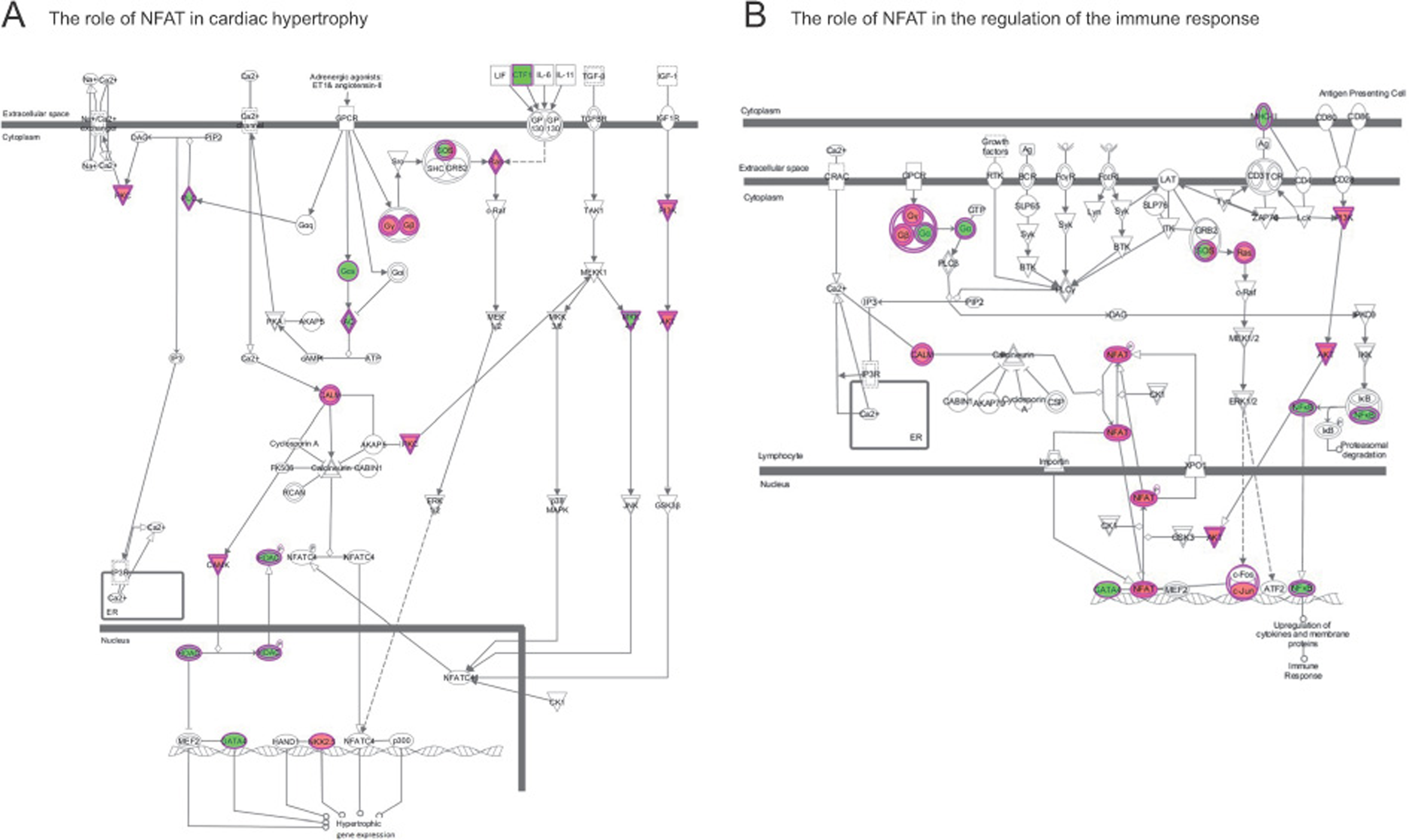

Supplement: Supplementary Figure 5 [file tp2015158x5.tif]
