# Supplementary material for: Transcriptomic and genetic studies identify NFAT5 as a candidate gene for cocaine dependence
Source: Transl Psychiatry. 2015 Oct 27;5(10):e667–. doi: 10.1038/tp.2015.158 (PMC4930134; doi:10.1038/tp.2015.158)
Supplement: Supplementary Figure Legends [file tp2015158x6.doc]

**Supplementary Figure 1. SH-SY5Y differentiation with retinoic acid (RA) during 7 days.** A) Neuron-like morphological changes observed in cultured cells after differentiation, including neurite outgrowth. B) Expression of tyrosine hydroxylase (TH) as a dopamine neuronal marker by Western blot. C) Cell cycle arrest in G0/G1.

**Supplementary Figure 2. Canonical pathways enriched in the differentially expressed genes identified 6 hours after exposure of SH-SY5Y RA-differentiated cells to 5 μM cocaine.**

**Supplementary Figure 3. Linkage disequilibrium among the genotyped SNPs of the *NFAT5* gene.** On top, schematic representation of the *NFAT5* gene. Boxes indicate exons, with the untranslated regions in grey. The six SNPs included in the study are shown on top. Linkage disequilibrium plot of the six SNPs according to Haploview showing r^2^ values. Considering the Confidence Interval algorithm^73^, three SNPs are located in one block in high LD (rs1437134, rs7359336 and rs6499244) and two SNPs lie in another block (rs11641233 and rs12232410), also in high LD in our control sample.

**Supplementary Figure 4. Associated SNPs and effect on microRNA binding.** Sequence alignments of the SNPs associated with cocaine dependence showing their predicted binding to microRNA molecules by TargetScan. Both alleles of each SNP are shown, indicated in bold, and the seed region of the microRNAs is underlined.

**Supplementary Figure 5. NFAT canonical pathways enriched in the differentially expressed genes identified 6 hours after exposure to 5 μM cocaine.** A) The canonical pathway “Role of NFAT in cardiac hypertrophy”. B) The canonical pathway “Role of NFAT in the regulation of the immune response”. The green and red nodes in the pathway indicate the down- and up-regulated genes, respectively, induced 6 hours after exposure to 5 μM cocaine.
