# Supplementary material for: Transcriptomic and genetic studies identify NFAT5 as a candidate gene for cocaine dependence
Source: Transl Psychiatry. 2015 Oct 27;5(10):e667–. doi: 10.1038/tp.2015.158 (PMC4930134; doi:10.1038/tp.2015.158)
Supplement: Supplementary Informations [file tp2015158x7.doc]

**SUPPLEMENTARY INFORMATION**

**1. COCAINE IN VITRO EXPERIMENTS AND MICROARRAYS**

**1.1. Cell culture, differentiation and cocaine treatment**

SH-SY5Y cells (ATCC, LGC Standards, Middlesex, UK) were cultured in previously poly-lysinated 100 mm dishes at 37ºC with 5% CO_2_ in 50:50 Dulbecco’s modified Eagle’s medium (DMEM) : F12 supplemented with 10% fetal bovine serum (FBS) and 1% Penicillin/Streptomycin (Gibco, Life Technologies Corporation, Carlsbad, California). All cells used in this study were between the 8^th^ and the 11^th^ passages. For differentiation to dopaminergic neuron-like cells, around 1.17e04 cells/cm^2^ were harvested with media supplemented with 10 μM retinoic acid (RA, Sigma-Aldrich Corporate, St. Louis, MO, USA) for 7 days, replacing with fresh RA medium on the 4^th^ day. SH-SY5Y differentiation was assessed by changes compatible with neuronal-like morphology and neurite outgrowth, expression of tyrosine-hydroxylase (TH) as a dopaminergic neuronal marker by Western blot, and cell cycle analysis (Supplementary Figure 1). Western blotting of tubulin was performed as described elsewhere^1^ and TH was tested with the same procedure using rabbit polyclonal anti-TH (Chemicon, EMD Millipore Corporation, Billerica, MA, USA) 1:500 dilution with an overnight incubation at 4ºC. Briefly, cell cycle analysis was performed by fixing cells with ethanol and incubating them with a propidium iodide solution (0.1% (v/v) Triton X-100 (Sigma-Aldrich Corporate, St. Louis, MO, USA) in PBS, 0.2 mg/ml RNAse A (DNAse free) (Sigma-Aldrich Corporate, St. Louis, MO, USA) and 200 μl propidium iodide 1 mg/ml (Molecular Probes, Life Technologies Corporation, Carlsbad, CA, USA) during 15 minutes at 37ºC, and then analyzed by flow cytometry.

The range of cocaine concentrations to be studied was selected based on concentrations determined in different human studies: i) in plasma of volunteers after a single dose (0.4 – 1.5 μM);^2^ ii) in plasma of cocaine abusers after repeated doses (1 – 5 μM);^3^ iii) in forensic plasma samples of cocaine abusers (0.8 – 13 μM in plasma);^4^ iv) plasma average concentration after death by cocaine overdose (20 μM);^5^ v) peak plasma levels after intravenous or smoking of the usual cocaine doses (1.6 – 3.3 μM);^6,7^ vi) in plasma of individuals correlating with significant increase of subjective “high” feeling, rush and craving, correlating with >60% DAT occupancy in a PET study (1 – 2.64 μM);^8^ vii) similar brain and plasma cocaine concentrations in humans after an intravenous administration (0.8 – 3 μM).^9^ For cytotoxicity experiments we considered the whole range of concentrations described in those studies (1 – 20 μM) and for the microarray experiments we used the lowest and highest concentrations in the normal range observed among cocaine abusers (1 – 5 μM),^3,9^ which were also used in a previous microarray study.^10^

Once the cells were differentiated, the medium with RA was replaced by fresh medium without RA during 24h before the exposure to cocaine, when the medium was again replaced by either medium without cocaine or medium containing 1 or 5 μM of cocaine hydrochloride (Alcaliber, Madrid, Spain). After 30 minutes of cocaine exposure, the medium was replaced by fresh medium and incubated during 6 or 24 h before retrieval for RNA extraction. Non-treated cells were exposed to the same media replacements. The six treatment groups consisted of untreated (control, 0 μM cocaine-HCl) differentiated SH-SY5Y cells retrieved at 6 and 24 h, differentiated SH-SY5Y cells treated with 1 μM cocaine-HCl retrieved at 6 and 24 h, and differentiated SH-SY5Y cells treated with 5 μM cocaine-HCl retrieved at 6 and 24 h. Nine dishes of cells were used for each condition.

**1.2. Cytotoxicity**

For evaluating cocaine cytotoxicity, an XTT assay was performed using the Cell Proliferation Kit II (XTT) (Roche Life Sciences, Branford, CT, USA), based on the cleavage of the yellow tetrazolium salt XTT to form an orange formazan dye by metabolically active cells. Briefly, SH-SY5Y cells were harvested in 24-well plates and differentiated with RA as described above. The medium was replaced by medium containing cocaine at different concentrations (0, 1, 5, 10, 15 and 20 μM) for an acute 30 or 60 minutes exposure, and then replaced by fresh medium. The XTT assay was performed at 24 or 48 hours after cocaine exposure, four replicates per condition, by replacing the medium with fresh medium supplemented with the XTT labelling reagent and the Electron Coupling Reagent as indicated in the manufacturer’s protocol. Absorbance was measured at 490nm in a 96-well plate 2 and 4h after adding the medium with XTT. Absorbance differences between conditions were evaluated using a U-Mann-Whitney non-parametric test, since normality was rejected using the Kolmogorov-Smirnov test (as expected given the small number of observations), and statistical significance was set at P<0.05 considering a two-sided test.

**1.3. RNA isolation and microarray hybridization**

For each condition (exposure to a given cocaine concentration for a preset time after an acute exposure), nine dishes containing treated SH-SY5Y differentiated cells were trypsinized, the pellets were homogenized using Qiashredder (Qiagen, Hilden, Germany) and total RNA was isolated using the RNeasy Mini Kit (Qiagen, Hilden, Germany). RNA concentration was determined using the NanoDrop ND-1000 spectrophotometer (NanoDrop Technologies, Termo Fisher Scientific Inc., Wilmington, DE, USA) and integrity was evaluated using the Bioanalyzer 2100 platform (Agilent Technologies, Santa Clara, CA, USA). The average RIN value (RNA Integrity Number) was 9.7, being 8.6 the lowest value. RNA samples were stored at -80ºC until analyzed. RNA pools from three dishes were used for each replicate in the microarray. For the microarray experiment we used the GeneChip® Human Genome U133 Plus 2.0 Array (Affymetrix Inc., Santa Clara, CA, USA), which contains probes that cover over 47,000 transcripts from over 38,500 well-characterized genes. A total of 18 chips were used: three pools per condition (0, 1 and 5 μM of cocaine-HCl) and two time points, 6 and 24 hours after an acute 30-min cocaine exposure. Two μg of RNA from each pool were used to hybridize arrays at the Genomics Unit of Hospital Clínic-IDIBAPS (Barcelona, Spain). Chips were scanned using a GenePix4000B scanner and raw data was obtained using the GenePix Pro 4.0 and the GCOS software. Microarray data have been deposited in NCBI's Gene Expression Omnibus (GEO) and are accessible through GEO Series accession number GSE71939 (<http://www.ncbi.nlm.nih.gov/geo/query/acc.cgi?acc=GSE71939>).

**1.4. Statistical and bioinformatics analysis of the expression microarrays**

Analysis of the microarray data was performed using the Bioconductor software for R environment and the *affy* library (www.bioconductor.org).^11^ The quality assessment of the chips was performed using the *affyPLM* library. Background correction, normalization and summarization were performed using the background, Robust Multichip Average (RMA)^12^ and median-polish methods, respectively. Genes were filtered by signal (threshold log_2_(40)), and we also discarded those probes that did not correspond to known genes. Genetic profiles at 6 and 24 hours were assessed separately in all steps to avoid interference, and a total number of 24,035 and 26,170 expressed probes (for 6 and 24 hours, respectively) were finally considered for the subsequent analyses. The Linear Modelling for Microarray Analysis (LIMMA) package^13^ was used for the class comparison, in which the expression patterns for 0 μM – 1 μM, 0 μM – 5 μM, and 1 μM – 5 μM conditions at 6 and 24 hours were compared. Corrections for multiple testing were applied by adjusting the p-values with a 10% False Discovery Rate (FDR). Functional group enrichment of genes showing significant differences in expression was performed using the DAVID Annotation Tool (http://david.abcc.ncifcrf.gov) considering GO biological processes (FAT category) and KEGG pathways.^14,15^ Canonical pathway enrichment analyses and gene networks were investigated with Ingenuity Pathway Analysis 8.8 software (http://www.ingenuity.com/products/ipa; Ingenuity Systems, Redwood city, CA, USA). MicroRNA binding sites enrichment analyses were performed using the WebGESTALT software (http://bioinfo.vanderbilt.edu/webgestalt) considering separately up-regulated and down-regulated genes.^16^

**1.5. Quantitative RT-PCR**

For quantitative Real Time PCR (qRT-PCR) validation we initially selected genes showing differences in expression ≥ 1.5 fold, a total of 143. From those, we considered genes included in representative enriched functional categories, pathways or gene networks. Finally, we selected eight genes based on their function and possible involvement in mediating cocaine effects that had not been previously identified to be altered by cocaine.

Validation of the selected results obtained in the microarray study was performed with the samples used in the microarray at 6 hours after cocaine exposure (0μM and 5μM). Gene expression was also assessed at different time points (2, 4, 5, 6, 7, 8 and 10 h) after cocaine acute exposure (0 μM or 5 μM) considering three replicates per condition. Cell culture and differentiation, cocaine treatment and RNA isolation were performed as described above.

Total RNA from the samples was transcribed using the High capacity cDNA Reverse Transcription Kit (Applied Biosystems, Foster City, CA, USA). QRT-PCR experiments were performed for eight genes using the LightCycler 480 II system and the Universal Probe Library (Life Sciences, Branford, CT, USA). Gene assays were designed with the Universal ProbeLibrary Assay Design Center software (Roche Life Sciences, Branford, CT, USA). Sequence of the primers and probes used are available upon request. Relative quantification was performed for each selected gene and glyceraldehyde-3-phosphate dehydrogenase (*GAPDH*) and hypoxanthine phosphoribosyltransferase (*HPRT1*) were used to normalize the relative amounts of mRNA. Gene expression changes for each comparison were evaluated using a U-Mann-Whitney non-parametric test, since normality was rejected using the Kolmogorov-Smirnov test (as expected given the small number of observations), and statistical significance was set at P<0.05 considering a two-sided test.

**1.6. Calcium imaging and neuronal activity monitoring**

We used calcium imaging in the cell cultures to monitor the changes in neuronal activity upon cocaine treatment in an area containing about 200 neurons. SH-SY5Y cells were grown and differentiated as described above in 35 mm dishes. Prior to imaging, dishes were gently washed with 4ml PBS at room temperature to remove the original culture medium. Next, we incubated the cultures for 45 min in a solution that contained 1ml of recording medium (RM, consisting of 128 mM NaCl, 1 mM CaCl_2_, 1 mM MgCl_2_, 45 mM sucrose, 10 mM glucose, and 0.01 M Hepes; pH 7.4) and 4 μg/ml of the cell-permeant calcium sensitive dye Fluo-4-AM. Then we washed the culture with 2 ml of fresh RM to remove residual free Fluo-4. This medium was discarded to place 4 ml of fresh RM, the final medium for actual recordings.

The culture dish was mounted on a Zeiss inverted microscope equipped with a 5X objective and a 0.32X optical zoom that provided a spatial resolution of 2.9 μm/pixel. Spontaneous neuronal activity was monitored through a Hamamatsu Orca Flash 2.8 CMOS camera attached to the microscope, in combination with a light source for fluorescence. Grey-scale images of neuronal activity were acquired at a speed of 13.3 frames per second (75 ms interval between two consecutive frames), and a size of 960x720 pixels. These settings provided a final field of view of 2.8x2.1 mm that contained 150-300 neurons.

For data acquisition and analysis we proceeded as follows: We first recorded spontaneous activity (i.e. without cocaine exposure) for 15 min to characterize the background activity of the network. We next added cocaine to the media to reach a concentration C_1_ (set as either 1 or 5 μM), and measured for 15 min. Finally, we increased the cocaine concentration to C_2_ =10 μM, and measured for additional 15 min. The settings of our recording device allowed the identification of single neuronal bodies with sufficient spatial resolution. Hence, we manually selected all available neurons as regions of interest (ROIs), and then extracted the average grey-scale level (i.e. the fluorescence amplitude) for each region as a function of time. Neuronal firing events were detected as a fast rise in the fluorescence signal, as illustrated in Figure 3.

For each selected neuron and condition (0 μM, 1 μM, 5 μM, and 10 μM of cocaine-HCl), we determined two network descriptors, namely the fraction of active neurons and the average activity of the network. Active cells were those that showed activity at any condition within our total recording time of 45 min. We typically observed about 20-30 active cells per culture. The fraction of active neurons in a culture was calculated as the number of cells that showed activity in a given condition divided by the total number of active cells monitored. The average activity of the network was determined as the total number of firings events divided by the total number of active cells. Data in both studies were finally averaged among different cultures. In total, for each condition, we averaged over eight different replicates per condition (a total of 160-240 active neurons per condition). Differences between conditions were evaluated using a U-Mann-Whitney non-parametric test, since normality was rejected using the Kolmogorov-Smirnov test (as expected given the small number of observations), and statistical significance was set at P<0.05 considering a one-sided test.

**2. CASE-CONTROL ASSOCIATION STUDY ON COCAINE DEPENDENCE**

**2.1. Subjects**

The patient sample consisted of 806 cocaine dependent subjects (mean age 35.7 ± 8 years and 81.4% males (*n =* 656)), from which 10.3% were dependent only to cocaine and not to any other substance (*n* = 83) (Supplementary Table 3). All patients were recruited and evaluated at the Psychiatry Department of the Hospital Universitari Vall d’Hebron (Barcelona, Spain) according to DSM-IV TR criteria (Diagnostic and Statistical Manual of Mental Disorders, 4th ed., text revision). The Structured Clinical Interview (SCID)^17^ was administered and volunteers with current DSM-IV diagnosis of dependence were included in the study. All patients were evaluated following a protocol of the Unit.^18^

The control sample consisted of 817 sex-matched healthy controls (mean age 55.9 ± 16 years, 81.8% males (*n* = 668)) (Supplementary Table 3). All controls were recruited at the Blood and Tissues Bank of Barcelona. None of them had injected drugs intravenously. Both patients and controls were Spanish and Caucasian, with the two last names (one from each parent) of Spanish origin. The study was approved by the Ethics Committee of Hospital Universitari Vall d’Hebron, and written informed consent was obtained from all participating individuals. Population stratification was previously discarded in our sample.^19^

**2.2. DNA isolation and quantification**

Genomic DNA samples were obtained from peripheral blood lymphocytes by a standard salting-out procedure and concentrations were determined using a NanoDrop ND-1000 spectrophotometer (NanoDrop Technologies, Willmington, DE, USA).

**2.3. SNP selection and genotyping**

For the case-control association study we searched for SNPs in the seven genes showing validated differential expression in neuronal cells treated with cocaine, according to both microarray and qRT-PCR experiments. The selected SNPs had MAF>0.15 in the CEU population (HapMap, http://www.hapmap.org), were located within the gene or in a region including 10 Kb upstream and 5 Kb downstream from the gene and were predicted to have a functional effect on the protein, microRNA binding, transcription factor binding or splicing by the SNP Function Prediction software (http://snpinfo.niehs.nih.gov/snpinfo/snpfunc.htm). Variants in two genes were not tested: *ENC1* did not have any SNP fulfilling these criteria and *NRG1* had three non-synonymous SNPs that were not predicted to be damaging by polyphen and, thus, were not considered. For *SEMA6D*, seven SNPs were previously found associated with substance dependence in a GWAS^20^ and two SNPs with MAF>0.15 that tag the associated variants, rs3809485 and rs4775708, were also included in our study. A total of 24 SNPs were selected and genotyping was performed using KASP technology (KBiosciences UK Ltd, UK). One SNP assay failed, so a total of 23 SNPs within five genes (*NFAT5*, *ELF1*, *PPP1R9A*, *SEMA6D* and *IGF2BP3*) were successfully genotyped.

**2.4. Case-control association study: statistical analysis**

The minimal statistical power was estimated *post hoc* using the software Power Calculator for Genetic Studies (http://sph.umich.edu/csg/abecasis/CaTS)^21^ under the additive model, assuming an odds ratio (OR) of 1.5 and a significance level of 0.05. For the calculation we considered the lowest MAF value in the control sample (0.143) and a prevalence of 0.0163 for cocaine dependence (10.2% of adult Spanish consumers from which 16% developing dependence (EMCDDA 2012 Annual Report)). The analysis of Hardy–Weinberg equilibrium (threshold set at P*=*0.01) and the comparison of genotype frequencies between cases and controls under the additive model was performed using the *SNPassoc* R package.^22^ Significant *P* values were adjusted for age. Bonferroni correction for multiple testing was applied considering 22 independent tests, with the threshold for significance set at P<0.0022.

**2.5. Linkage Disequilibrium assessment**

To assess linkage disequilibrium (LD) between genotyped SNPs in the *NFAT5* gene we used the Haploview 4.2 software^23^ and considered our genotyped control sample. We considered that SNPs were in high linkage disequilibrium if r^2^ >0.9.

**3. FUNCTIONAL EFFECT OF ASSOCIATED SNPs**

**3.1. Effect of SNP variants on the regulation of gene expression by microRNAs using a reporter system**

All associated SNPs were predicted to have a functional effect on microRNA binding by the SNP Function Prediction software. We first checked if those microRNAs had validated target sites in the predicted regions of *NFAT5* 3’UTR region using DIANA-TarBase (http://diana.imis.athena-innovation.gr/DianaTools/index.php?r=tarbase/). To assess potential functional effect of these SNPs we further checked with other programs: mrSNP (http://mrsnp.osu.edu/), mirSNP (http://202.38.126.151/hmdd/mirsnp/search/) and miRdSNP (http://mirdsnp.ccr.buffalo.edu/). Only SNPs rs1437134 and rs11641233 showed consistent predictions and seemed to alter hsa-mir-509-5p and hsa-miR-649 binding, respectively. Additionally, TargetScan (http://www.targetscan.org/) predicted binding of hsa-mir-509-5p and hsa-miR-649 to the genomic regions corresponding to SNPs rs1437134 and rs11641233, respectively.

We used a luciferase reporter system to test the effect of rs1437134 and rs11641233 on *NFAT5* gene expression. We used the pmirGLO Dual-Luciferase microRNA Target Expression Vector (Promega, Madison, WI, USA) to clone the 3’UTR regions containing the SNPs. This vector allows the evaluation of microRNA binding to the predicted sites by cloning the microRNA target region 3’ from the firefly luciferase gene (*luc2*), which is the primary reporter gene. The vector also contains the Renilla luciferase gene (hRluc-neo) to normalize expression.

The regions containing the SNPs rs1437134 and rs11641233 were amplified by PCR in heterozygous individuals using the following primers containing the restriction sites *Nhe*I and *Xba*I (underlined): rs1437134 primers 5’-ACACGCTAGCTGACTGCAAAAGAGCACACC-3’ and 5’- ACAATCTAGAAGGAATTGCGCCTAGGAA-3’, and rs11641233 primers 5’-ACACGCTAGCTTTTTAAATTAATTAGCTTTCCTCTGC-3’ and 5’- AGAATCTAGATGGGACCATCCACAAATGAT-3’ (Sigma-Aldrich Corporate, St. Louis, MO, USA). PCR products were purified using PCR-Illustra GFX PCR and Gel Band purification kit (GE Healthcare, Waukesha, WI, USA). PCR fragments and pmirGLO Dual-Luciferase microRNA Target Expression Vector were digested with *Nhe*I and *Xba*I and the Cut Smart buffer (New England Biolabs, Ipswich, MA, USA) for 3 hours at 37ºC. The vector was dephosphorylated with the Shrimp Alkaline Phosphatase (SAP, Promega, Madison, WI, USA) for 15 minutes at 37ºC and inactivated during 15 minutes at 65 ºC. Ligation was performed at 16ºC O/N with the DNA ligation kit version 2.1 (Takara Bio Inc., Otsu, Shiga, Japan) considering a 3:1 insert:vector molecular proportion. Ligation was transformed in Subcloning Efficiency™ DH5α™ Competent Cells (Invitrogen™, Paisley, Scotland, UK) following manufacturer’s recommendations. Plasmidic DNA extraction was performed with the High Pure Plasmid Isolation Kit and Genopure Plasmid Midi-Kit (Roche Life Sciences, Branford, CT, USA). Sanger sequenciation was performed using the BigDye Terminator v3.1 Cycle Sequencing Kit (Applied Biosystems, Foster City, CA, USA). The following constructs were obtained: pmiRGLO–rs1437134A, pmiRGLO–rs1437134G, pmiRGLO–rs11641233C and pmiRGLO–rs11641233T.

The microRNAs genes hsa-miR-509-1 and hsa-miR-649 were obtained as clones into the pCMV-MIR vector (OriGene, Rockville, MD, USA), which contains a GFP reporter for transfection. All clones used in the study were sequenced in full to confirm their integrity.

Experiments were performed in HeLa and in SH-SY5Y cells, which were previously discarded to be contaminated with mycoplasm with the kit Mycoplasma gel detection kit (Biotools B&M Labs S.A., Madrid, Spain).

HeLa cells were cultured in 12-well plates with DMEM, 10% FBS and 1% Penicillin/Streptomycin (Gibco, Life technologies Corporation, Carlsbad, CA, USA) in a 5% CO_2_ humidified atmosphere at 37 °C. For each condition we always co-transfected two constructs in a 1:1 molecular ratio considering 530 ng for all pmirGLO constructs and 470 ng for all microRNAs constructs. When the pmirGLO constructs were not cotransfected with a microRNA construct we used 250 ng of pmax GFP vector (Lonza, Basel, Switzerland). Each well plate was transfected with 3 μl Lipofectamine 2000 Reagent (Invitrogen™, Paisley, Scotland, UK) and the following constructs: a) pmiR-Glo and pmax GFP, b) pmiR-Glo and pCMV-hsa-miR-509-1, c) pmiR-Glo – rs1437134>A and pmax GFP, d) pmiR-Glo – rs1437134>A and pCMV-hsa-miR-509-1, e) pmiR-Glo – rs1437134>G and pmax GFP, f) pmiR-Glo – rs1437134>G and pCMV-hsa-miR-509-1, g) pmiR-Glo and pCMV-hsa-miR-649, h) pmiR-Glo – rs11641233>C and pmax GFP, i) pmiR-Glo – rs11641233>C and pCMV-hsa-miR-649, j) pmiR-Glo – rs11641233>T and pmax GFP, k) pmiR-Glo – rs11641233>T and pCMV-hsa-miR-649. Five plate replicates were used for the experiment. Cells were retrieved 24 hours after transfection and washed with PBS twice. Cell lysates were obtained using the Passive Lysis Buffer (Promega, Madison, WI, USA) and were stored at -80ºC.

In order to confirm the effect of rs1437134 on gene expression in another cell line, SH-SY5Y cells were cultured in 6-well plates as specified above (Page 1), without following differentiation protocol. For transfection we used the Amaxa Cell Line Nucleofector Kit V (Lonza, Basel, Switzerland) following the manufacturer’s instructions. For each condition performed for rs1437134 (a-f, as detailed above), 2×10^6^ cells/well were transfected, and three experimental replicates were used for the experiments. Cells were also retrieved 24 hours after transfection and lysis was performed as described above.

Luciferase expression assays were performed by technical duplicates using the Dual-Luciferase Reporter Assay System (Promega, Madison, WI, USA) and following the manufacturer’s protocol for two injector luminometers. We used the luminometer Modulus Microplate Multimode Reader (Turner BioSystems, Sunnyvale, CA, USA). Relative luciferase expression (luciferase/renilla expression) of each allele was normalized to their corresponding control (pmirGLO or pmirGLO with the corresponding microRNA). Differences between conditions were evaluated using a U-Mann-Whitney non-parametric test, since normality was rejected using the Kolmogorov-Smirnov test (as expected given the small number of samples), and statistical significance was set at P<0.05 considering a two-sided test.

**3.2. SNPs correlation with brain volume differences using brain imaging analysis**

The Brain Imaging Genetics (BIG; http://www.cognomics.nl) study consists of healthy volunteers who participated in diverse small-scale studies at the Donders Centre for Cognitive Neuroimaging, Nijmegen, The Netherlands.^24^ The present study included a subset of 1300 BIG subjects (744 females; mean age 22.9 (SD = 3.8)) who had undergone anatomical (T1-weighted) MRI scans and for whom genome-wide SNP genotype data were available.

MRI data were acquired using either 1.5 Tesla (n=645) or 3 Tesla (n=655) Siemens scanners (Siemens Medical Systems, Erlangen, Germany). Details on scanning parameters and other neuroimaging procedures used in BIG are described in Guadalupe et al.^25^ Automated parcellation of cerebral cortical regions was performed according to the Desikan atlas^26^ using the FreeSurfer package.^27^ Subcortical segmentations were also produced using FreeSurfer with the standard “-recon-all” processing pipeline and default parameters. Volumes were available for the following seven addiction associated brain structures^28^: medial orbitofrontal cortex, prefrontal cortex (defined as superior frontal gyrus, rostral middle frontal gyrus, caudal middle frontal gyrus, inferior frontal gyrus, lateral and medial divisions of orbitofrontal cortex and frontal pole), and bilateral nucleus accumbens, putamen, caudate nucleus, hippocampus and insula. Ventral tegmental area (VTA), important in dopaminergic pathways of the reward system, could not be assessed. Estimates of total brain volume (TBV) were calculated as the voxel-wise sum of the gray matter and white matter probability maps produced by the VBM8 toolbox (http://dbm.neuro.uni-jena.de/vbm/), implemented in SPM8 (http://www.fil.ion.ucl.ac.uk/spm/). Outliers more extreme than 3.5 SD from the mean were excluded from analysis.

Genotyping and imputation procedures performed in BIG are described in detail in Guadalupe et al.^29^ In short, whole genome genotyping was done using the Affymetrix Genome-Wide Human SNP Array 6.0 (Affymetrix Inc., Santa Clara, CA, USA). MACH software was used for haplotype phasing and minimac for the final imputation, ^30,31^with the 1000 Genomes Phase 1.v3 EUR reference panel.^32^ SNPs rs1437134, rs7359336, rs11641233, rs6499244 and rs12232410 were extracted from the data for the first stage of analysis using PLINK v1.07.^33^ Since rs7359336 and rs6499244 were in strong linkage disequilibrium (LD) with rs1437134 (r^2^ > .9), and rs12232410 was in strong LD with rs11641233 (r^2^ >.9), only rs1437134 and rs11641233 (r^2^=.207) were included in the analysis. For a second gene-wide step of analysis, all available variants of the *NFAT5* gene were extracted, including a 100 kb flanking region to capture regulatory sequences. SNPs failing Hardy-Weinberg equilibrium test at a threshold of P<10-6, with minor allele frequency lower than 1% or with missingness rates higher than 5% were removed from the data. Participants with call rates lower than 95% were also excluded from analysis. For the gene-wide analysis, linkage disequilibrium pruned genotypes were used (r^2^ threshold of .8). Eighty SNPs were left for analysis.

We carried out our analysis in two stages. First, associations of *NFAT5* SNPs rs1437134 and rs11641233 with volumes of prefrontal cortex, medial orbitofrontal cortex, nucleus accumbens, putamen, caudate nucleus, hippocampus and insula were assessed using linear regression as implemented in PLINK v1.07.^33^ Results were considered significant at P<0.05 divided by the number of structures and SNPs for each cohort (P<0.0036). As a second step, the effect of common variants of *NFAT5* on the brain volumes was investigated using the statistical approach described by Bralten et al.^34^ consisting of SNP-by-SNP linear regression and estimation of the effect of the whole gene. The BIG subjects scanned at 1.5 T were used as the discovery sample in all analyses and replication was attempted in the independent sample scanned at 3.0 T. In addition, the analyses were performed in the combined sample. All analyses were adjusted for gender, age and total brain volume. Field strength was added as a covariate for the combined analysis.

**REFERENCES**

1. Rodriguez-Pascau L, Gort L, Schuchman EH, Vilageliu L, Grinberg D, Chabas A. Identification and characterization of SMPD1 mutations causing Niemann-Pick types A and B in Spanish patients. *Hum Mutat* 2009; **30:** 1117-1122.

2. Van Dyke C, Barash PG, Jatlow P, Byck R. Cocaine: plasma concentrations after intranasal application in man. *Science* 1976; **191:** 859-861.

3. Jufer RA, Walsh SL, Cone EJ. Cocaine and metabolite concentrations in plasma during repeated oral administration: development of a human laboratory model of chronic cocaine use. *J Anal Toxicol* 1998; **22:** 435-444.

4. Stephens BG, Jentzen JM, Karch S, Mash DC, Wetli CV. Criteria for the interpretation of cocaine levels in human biological samples and their relation to the cause of death. *Am J Forensic Med Pathol* 2004; **25:** 1-10.

5. Ellenhorn MJ, Barceloux DG. *Medical toxicology* Elsevier: New York, 1988, 644-661pp.

6. Jatlow P. Cocaine: analysis, pharmacokinetics, and metabolic disposition. *Yale J Biol Med* 1988; **61:** 105-113.

7. Paly D, Jatlow P, Van Dyke C, Jeri FR, Byck R. Plasma cocaine concentrations during cocaine paste smoking. *Life Sci* 1982; **30:** 731-738.

8. Volkow ND, Wang GJ, Fischman MW, Foltin RW, Fowler JS, Abumrad NN *et al.* Relationship between subjective effects of cocaine and dopamine transporter occupancy. *Nature* 1997; **386:** 827-830.

9. Fowler JS, Volkow ND, Wolf AP, Dewey SL, Schlyer DJ, Macgregor RR *et al.* Mapping cocaine binding sites in human and baboon brain in vivo. *Synapse* 1989; **4:** 371-377.

10. Crawford FC, Wood ML, Wilson SE, Mathura VS, Hollen TR, Geall F *et al.* Cocaine induced inflammatory response in human neuronal progenitor cells. *J Neurochem* 2006; **97:** 662-674.

11. Gentleman RC, Carey VJ, Bates DM, Bolstad B, Dettling M, Dudoit S *et al.* Bioconductor: open software development for computational biology and bioinformatics. *Genome Biol* 2004; **5:** R80.

12. Irizarry RA, Hobbs B, Collin F, Beazer-Barclay YD, Antonellis KJ, Scherf U *et al.* Exploration, normalization, and summaries of high density oligonucleotide array probe level data. *Biostatistics* 2003; **4:** 249-264.

13. Smyth GK. Linear models and empirical bayes methods for assessing differential expression in microarray experiments. *Stat Appl Genet Mol Biol* 2004; **3:** Article3.

14. Dennis G, Jr., Sherman BT, Hosack DA, Yang J, Gao W, Lane HC *et al.* DAVID: Database for Annotation, Visualization, and Integrated Discovery. *Genome Biol* 2003; **4:** P3.

15. Huang da W, Sherman BT, Lempicki RA. Systematic and integrative analysis of large gene lists using DAVID bioinformatics resources. *Nat Protoc* 2009; **4:** 44-57.

16. Zhang B, Kirov S, Snoddy J. WebGestalt: an integrated system for exploring gene sets in various biological contexts. *Nucleic Acids Res* 2005; **33:** W741-748.

17. First M, Spitzer R, Gibbon M, JBW W. *Structured Clinical Interview for DSM-IV disorders (SCID-IV)*. Washington, DC: American Psychiatric Press.1997.

18. Roncero C, Daigre C, Barral C, Ros-Cucurull E, Grau-Lopez L, Rodriguez-Cintas L *et al.* Neuroticism associated with cocaine-induced psychosis in cocaine-dependent patients: a cross-sectional observational study. *PLoS One* 2014; **9:** e106111.

19. Fernandez-Castillo N, Roncero C, Grau-Lopez L, Barral C, Prat G, Rodriguez-Cintas L *et al.* Association study of 37 genes related to serotonin and dopamine neurotransmission and neurotrophic factors in cocaine dependence. *Genes Brain Behav* 2013; **12:** 39-46.

20. Drgon T, Zhang PW, Johnson C, Walther D, Hess J, Nino M *et al.* Genome wide association for addiction: replicated results and comparisons of two analytic approaches. *PLoS One* 2010; **5:** e8832.

21. Skol AD, Scott LJ, Abecasis GR, Boehnke M. Joint analysis is more efficient than replication-based analysis for two-stage genome-wide association studies. *Nat Genet* 2006; **38:** 209-213.

22. Gonzalez JR, Armengol L, Sole X, Guino E, Mercader JM, Estivill X *et al.* SNPassoc: an R package to perform whole genome association studies. *Bioinformatics* 2007; **23:** 644-645.

23. Barrett JC, Fry B, Maller J, Daly MJ. Haploview: analysis and visualization of LD and haplotype maps. *Bioinformatics* 2005; **21:** 263-265.

24. Franke B, Vasquez AA, Veltman JA, Brunner HG, Rijpkema M, Fernandez G. Genetic variation in CACNA1C, a gene associated with bipolar disorder, influences brainstem rather than gray matter volume in healthy individuals. *Biol Psychiatry* 2010; **68:** 586-588.

25. Guadalupe T, Zwiers MP, Wittfeld K, Teumer A, Vasquez AA, Hoogman M *et al.* Asymmetry within and around the human planum temporale is sexually dimorphic and influenced by genes involved in steroid hormone receptor activity. *Cortex* 2015; **62:** 41-55.

26. Desikan RS, Segonne F, Fischl B, Quinn BT, Dickerson BC, Blacker D *et al.* An automated labeling system for subdividing the human cerebral cortex on MRI scans into gyral based regions of interest. *Neuroimage* 2006; **31:** 968-980.

27. Fischl B, Salat DH, Busa E, Albert M, Dieterich M, Haselgrove C *et al.* Whole brain segmentation: automated labeling of neuroanatomical structures in the human brain. *Neuron* 2002; **33:** 341-355.

28. Volkow ND, Fowler JS, Wang GJ. The addicted human brain viewed in the light of imaging studies: brain circuits and treatment strategies. *Neuropharmacology* 2004; **47 Suppl 1:** 3-13.

29. Guadalupe T, Zwiers MP, Teumer A, Wittfeld K, Vasquez AA, Hoogman M *et al.* Measurement and genetics of human subcortical and hippocampal asymmetries in large datasets. *Hum Brain Mapp* 2014; **35:** 3277-3289.

30. Howie BN, Donnelly P, Marchini J. A flexible and accurate genotype imputation method for the next generation of genome-wide association studies. *PLoS Genet* 2009; **5:** e1000529.

31. Li Y, Willer CJ, Ding J, Scheet P, Abecasis GR. MaCH: using sequence and genotype data to estimate haplotypes and unobserved genotypes. *Genet Epidemiol* 2010; **34:** 816-834.

32. 1000 Genomes Project Consortium, Abecasis GR, Altshuler D, Auton A, Brooks LD, Durbin RM *et al.* A map of human genome variation from population-scale sequencing. *Nature* 2010; **467:** 1061-1073.

33. Purcell S, Neale B, Todd-Brown K, Thomas L, Ferreira MA, Bender D *et al.* PLINK: a tool set for whole-genome association and population-based linkage analyses. *Am J Hum Genet* 2007; **81:** 559-575.

34. Bralten J, Arias-Vasquez A, Makkinje R, Veltman JA, Brunner HG, Fernandez G *et al.* Association of the Alzheimer's gene SORL1 with hippocampal volume in young, healthy adults. *Am J Psychiatry* 2011; **168:** 1083-1089.
