# Supplementary material for: Transcriptomic and genetic studies identify NFAT5 as a candidate gene for cocaine dependence
Source: Transl Psychiatry. 2015 Oct 27;5(10):e667–. doi: 10.1038/tp.2015.158 (PMC4930134; doi:10.1038/tp.2015.158)
Supplement: Supplementary Table 1 [file tp2015158x8.doc]

| **Supplementary Table 1**. Descriptive characteristics of Spanish Caucasian individuals with cocaine dependence and controls | | | | | |
| --- | --- | --- | --- | --- | --- |
|  |  |  |  |  |  |
|  |  | **Cocaine dependence sample**  **N = 806** |  | **Control sample**  **N = 817** |  |
|  | **Gender N (%)** |  |  |  |  |
|  |  |  |  |  |  |
|  | Male | 656 (81.4) |  | 668 (81.8) |  |
|  | Female | 150 (18.6) |  | 149 (18.2) |  |
|  |  |  |  |  |  |
|  | **Age (mean and SD)** |  |  |  |  |
|  |  |  |  |  |  |
|  |  | 35.7 ± 8.0 |  | 55.9 ± 15.9 |  |
|  |  |  |  |  |  |
|  | **Substance Dependence N (%)** |  |  |  |  |
|  |  |  |  |  |  |
|  | **Total SD*** |  |  |  |  |
|  | Cocaine | 806 (100) |  | - |  |
|  | Only cocaine dependence** | 83 (10.3) |  | - |  |
|  | Alcohol | 230 (28.6) |  | - |  |
|  | Cannabis | 265 (32.9) |  | - |  |
|  | Opiates | 165 (20.5) |  | - |  |
|  | Tobacco | 595 (73.8) |  | - |  |
|  | Benzodiacepines | 57 (7.1) |  | - |  |
|  | Amphetamines and metamphetamines | 20 (2.5) |  | - |  |
|  | Ecstasy | 12 (1.5) |  | - |  |
|  | Others | 6 (0.7) |  | - |  |
|  |  |  |  |  |  |
|  | **Psychotic symptoms N (%) Available data in 79.3% patients** |  |  |  |  |
|  |  |  |  |  |  |
|  | Presence | 393 (48.8) |  | - |  |
|  | Absence | 246 (30.5) |  | - |  |
|  |  |  |  |  |  |

*Substance Dependence

**Cocaine dependent patients without any other drug use, abuse or dependence but occasional alcohol use (not considering tobacco).
