# Supplementary material for: Transcriptomic and genetic studies identify NFAT5 as a candidate gene for cocaine dependence
Source: Transl Psychiatry. 2015 Oct 27;5(10):e667–. doi: 10.1038/tp.2015.158 (PMC4930134; doi:10.1038/tp.2015.158)
Supplement: Supplementary Table 2 [file tp2015158x9.doc]

| **Supplementary Table 2.** Descriptive characteristics of the Brain Imaging Genetics (BIG; http://www.cognomics.nl) study consisting of self-reported healthy adults | | | | | |
| --- | --- | --- | --- | --- | --- |
|  |  |  |  |  |  |
|  |  | **Discovery sample**  **(1.5 Tesla) N=645** | **Replication sample**  **(3 Tesla) N=655** | **Complete sample**  **N=1300** |  |
|  | **Gender N (%)** |  |  |  |  |
|  |  |  |  |  |  |
|  | Male | 270 (41.9) | 286 (43.7) | 556 (42.8) |  |
|  | Female | 375 (58.1) | 369 (56.3) | 744 (57.2) |  |
|  |  |  |  |  |  |
|  | **Age (mean and SD)** |  |  |  |  |
|  |  |  |  |  |  |
|  |  | 23.12 ± 4.0 | 22.73 ± 3.6 | 22.73 ± 3.6 |  |
|  |  |  |  |  |  |
|  | **Total Brain Volume (mean and SD)** |  |  |  |  |
|  |  |  |  |  |  |
|  | TBV | 1252.75 ± 112.8 | 1240.68 ± 117.6 | 1246.30 ± 114.6 |  |
|  |  |  |  |  |  |
|  | **Brain Volumes (mean and SD)** |  |  |  |  |
|  |  |  |  |  |  |
|  | Prefrontal cortex | 154852.32* ± 15122.2 | 152827.84* ± 15871.8 | 153833.07* ± 15531.2 |  |
|  | Insula | 15021.29* ± 1652.8 | 14942.11* ± 1654.2 | 14981.36* ± 1653.3 |  |
|  | Hippocampus | 8810.20* ± 858.0 | 8994.33* ± 858.7 | 8902.91* ± 862.9 |  |
|  | Nucleus accumbens | 1067.48* ± 179.4 | 1098.29* ± 169.3 | 1083.03* ± 175.0 |  |
|  | Caudate nucleus | 8028.29* ± 970.9 | 8029.19* ± 954.0 | 8028.75* ± 962.1 |  |
|  | Putamen | 10959.71* ± 1265.5 | 11209.01* ± 1228.1 | 11085.32* ± 1252.5 |  |
|  | Medial orbitofrontal cortex | 11418.03* ± 1256.11 | 10771.15* ± 1239.4 | 11092.10* ± 1288.5 |  |

*mm^3^
