# Supplementary material for: Transcriptomic and genetic studies identify NFAT5 as a candidate gene for cocaine dependence
Source: Transl Psychiatry. 2015 Oct 27;5(10):e667–. doi: 10.1038/tp.2015.158 (PMC4930134; doi:10.1038/tp.2015.158)
Supplement: Supplementary Table 3 [file tp2015158x10.doc]

| **Supplementary Table 3.** Genes differentially expressed 6 hours after exposure of SH-SY5Y cells differentiated to dopaminergic neurons to 5 μM cocaine | | |
| --- | --- | --- |
|  |  |  |
|  | | **Probe** | **Gene symbol** | **Gene Name** | **P-value** | **Adj. P-value** | **5 – 0 μM**  **Fold Change** | | --- | --- | --- | --- | --- | --- | | **Up-regulated** | |  |  |  |  | |  |  |  |  |  |  | | 216450_x_at | HSP90B1 | heat shock protein 90kDa beta (Grp94), member 1 | 1.73E-06 | 0.014 | 2.17 | | 215992_s_at | RAPGEF2 | Rap guanine nucleotide exchange factor (GEF) 2 | 2.70E-06 | 0.014 | 1.57 | | 224321_at | TMEFF2 | transmembrane protein with EGF-like and two follistatin-like domains 2 | 3.39E-06 | 0.014 | 1.58 | | 221088_s_at | PPP1R9A | protein phosphatase 1, regulatory (inhibitor) subunit 9A | 5.31E-06 | 0.014 | 1.74 | | 209254_at | KIAA0265 | KIAA0265 protein | 5.47E-06 | 0.014 | 1.90 | | 202773_s_at | SFRS8 | splicing factor, arginine/serine-rich 8 (suppressor-of-white-apricot homolog, Drosophila) | 6.16E-06 | 0.014 | 1.67 | | 230629_s_at | EP400 | E1A binding protein p400 | 6.40E-06 | 0.014 | 1.70 | | 1558747_at | SMCHD1 | structural maintenance of chromosomes flexible hinge domain containing 1 | 6.43E-06 | 0.014 | 2.17 | | 207937_x_at | FGFR1 | fibroblast growth factor receptor 1 (fms-related tyrosine kinase 2, Pfeiffer syndrome) | 7.67E-06 | 0.014 | 1.45 | | 210943_s_at | LYST | lysosomal trafficking regulator | 9.29E-06 | 0.015 | 1.59 | | 215886_x_at | USP12 | ubiquitin specific peptidase 12 | 1.06E-05 | 0.015 | 1.70 | | 213328_at | NEK1 | NIMA (never in mitosis gene a)-related kinase 1 | 1.09E-05 | 0.015 | 1.74 | | 232940_s_at | MLL3 | myeloid/lymphoid or mixed-lineage leukemia 3 | 1.14E-05 | 0.015 | 1.48 | | 238350_at | FLJ25778 | hypothetical protein FLJ25778 | 1.14E-05 | 0.015 | 1.88 | | 223578_x_at | PRO1073 | PRO1073 protein | 1.22E-05 | 0.015 | 2.21 | | 209088_s_at | UBN1 | ubinuclein 1 | 1.23E-05 | 0.015 | 1.49 | | 232632_at | TSPAN3 | tetraspanin 3 | 1.31E-05 | 0.015 | 1.82 | | 209678_s_at | PRKCI | protein kinase C, iota | 1.33E-05 | 0.015 | 1.37 | | 227510_x_at | PRO1073 | PRO1073 protein | 1.41E-05 | 0.015 | 2.20 | | 1555450_a_at | NARG1L | NMDA receptor regulated 1-like | 1.55E-05 | 0.016 | 1.39 | | 213494_s_at | YY1 | YY1 transcription factor | 1.85E-05 | 0.018 | 1.41 | | 231735_s_at | PRO1073 | PRO1073 protein | 2.47E-05 | 0.021 | 1.47 | | 240434_at | ABLIM2 | actin binding LIM protein family, member 2 | 2.83E-05 | 0.022 | 1.38 | | 208930_s_at | ILF3 | interleukin enhancer binding factor 3, 90kDa | 3.07E-05 | 0.023 | 1.54 | | 204180_s_at | ZBTB43 | zinc finger and BTB domain containing 43 | 4.04E-05 | 0.026 | 1.45 | | 208232_x_at | NRG1 | neuregulin 1 | 4.28E-05 | 0.027 | 1.59 | | 1554740_a_at | IPP | intracisternal A particle-promoted polypeptide | 4.51E-05 | 0.027 | 1.47 | | 224030_s_at | FAM115A | family with sequence similarity 115, member A | 4.53E-05 | 0.027 | 1.45 | | 201340_s_at | ENC1 | ectodermal-neural cortex (with BTB-like domain) | 4.76E-05 | 0.028 | 1.67 | | 203176_s_at | TFAM | transcription factor A, mitochondrial | 5.63E-05 | 0.030 | 1.39 | | 236371_s_at | TGS1 | trimethylguanosine synthase homolog (S. cerevisiae) | 6.00E-05 | 0.030 | 1.49 | | 239898_x_at | ZNF286A | zinc finger protein 286A | 6.24E-05 | 0.030 | 1.32 | | 238536_at | LOC642236 | similar to FRG1 protein (FSHD region gene 1 protein) | 6.36E-05 | 0.030 | 1.33 | | 237209_s_at | NFRKB | nuclear factor related to kappaB binding protein | 6.67E-05 | 0.030 | 1.39 | | 1555945_s_at | FAM120A | family with sequence similarity 120A | 6.81E-05 | 0.030 | 1.33 | | 242911_at | MED13L | mediator complex subunit 13-like | 6.99E-05 | 0.030 | 1.51 | | 202818_s_at | TCEB3 | transcription elongation factor B (SIII), polypeptide 3 (110kDa, elongin A) | 7.04E-05 | 0.030 | 1.58 | | 212451_at | KIAA0256 | KIAA0256 gene product | 7.34E-05 | 0.030 | 1.82 | | 232021_at | GLT8D3 | glycosyltransferase 8 domain containing 3 | 7.37E-05 | 0.030 | 1.67 | | 227402_s_at | C8orf53 | chromosome 8 open reading frame 53 | 7.45E-05 | 0.030 | 1.32 | | 224568_x_at | MALAT1 | metastasis associated lung adenocarcinoma transcript 1 (non-protein coding) | 7.76E-05 | 0.031 | 2.45 | | 222027_at | NUCKS1 | nuclear casein kinase and cyclin-dependent kinase substrate 1 | 7.98E-05 | 0.031 | 1.78 | | 225452_at | MED1 | mediator complex subunit 1 | 9.36E-05 | 0.035 | 1.40 | | 233985_x_at | PPP1R9A | protein phosphatase 1, regulatory (inhibitor) subunit 9A | 1.00E-04 | 0.037 | 1.35 | | 239022_at | SDHALP2 | succinate dehydrogenase complex, subunit A, flavoprotein pseudogene 2 | 1.03E-04 | 0.037 | 1.74 | | 205184_at | GNG4 | guanine nucleotide binding protein (G protein), gamma 4 | 1.09E-04 | 0.037 | 1.34 | | 213812_s_at | CAMKK2 | calcium/calmodulin-dependent protein kinase kinase 2, beta | 1.09E-04 | 0.037 | 1.32 | | 214600_at | TEAD1 | TEA domain family member 1 (SV40 transcriptional enhancer factor) | 1.10E-04 | 0.037 | 1.66 | | 1558173_a_at | LUZP1 | leucine zipper protein 1 | 1.11E-04 | 0.037 | 1.58 | | 213971_s_at | SUZ12P | suppressor of zeste 12 homolog pseudogene | 1.11E-04 | 0.037 | 1.28 | | 205315_s_at | SNTB2 | syntrophin, beta 2 (dystrophin-associated protein A1, 59kDa, basic component 2) | 1.15E-04 | 0.037 | 1.35 | | 204964_s_at | SSPN | sarcospan (Kras oncogene-associated gene) | 1.15E-04 | 0.037 | 1.47 | | 208844_at | VDAC3 | voltage-dependent anion channel 3 | 1.31E-04 | 0.040 | 1.29 | | 212078_s_at | MLL | myeloid/lymphoid or mixed-lineage leukemia (trithorax homolog, Drosophila) | 1.36E-04 | 0.041 | 1.35 | | 213281_at | JUN | jun oncogene | 1.51E-04 | 0.041 | 1.42 | | 227514_at | LOC162073 | hypothetical protein LOC162073 | 1.53E-04 | 0.041 | 1.43 | | 220342_x_at | EDEM3 | ER degradation enhancer, mannosidase alpha-like 3 | 1.55E-04 | 0.041 | 1.50 | | 1559548_at | ACVR2B | activin A receptor, type IIB | 1.57E-04 | 0.041 | 1.41 | | 243295_at | RBM27 | RNA binding motif protein 27 | 1.58E-04 | 0.041 | 1.50 | | 202412_s_at | USP1 | ubiquitin specific peptidase 1 | 1.58E-04 | 0.041 | 1.46 | | 212420_at | ELF1 | E74-like factor 1 (ets domain transcription factor) | 1.58E-04 | 0.041 | 1.74 | | 208664_s_at | TTC3 | tetratricopeptide repeat domain 3 | 1.60E-04 | 0.041 | 1.52 | | 209433_s_at | PPAT | phosphoribosyl pyrophosphate amidotransferase | 1.65E-04 | 0.042 | 1.39 | | 208325_s_at | AKAP13 | A kinase (PRKA) anchor protein 13 | 1.72E-04 | 0.042 | 1.60 | | 215220_s_at | TPR | translocated promoter region (to activated MET oncogene) | 1.74E-04 | 0.042 | 1.70 | | 214474_at | PRKAB2 | protein kinase, AMP-activated, beta 2 non-catalytic subunit | 1.78E-04 | 0.042 | 1.61 | | 200702_s_at | DDX24 | DEAD (Asp-Glu-Ala-Asp) box polypeptide 24 | 1.81E-04 | 0.043 | 1.32 | | 227039_at | AKAP13 | A kinase (PRKA) anchor protein 13 | 1.91E-04 | 0.044 | 1.56 | | 242617_at | TMED8 | transmembrane emp24 protein transport domain containing 8 | 1.96E-04 | 0.044 | 1.23 | | 201831_s_at | USO1 | USO1 homolog, vesicle docking protein (yeast) | 1.97E-04 | 0.044 | 1.61 | | 1555913_at | GON4L | gon-4-like (C. elegans) | 1.99E-04 | 0.044 | 1.47 | | 212649_at | DHX29 | DEAH (Asp-Glu-Ala-His) box polypeptide 29 | 2.11E-04 | 0.045 | 1.59 | | 203532_x_at | CUL5 | cullin 5 | 2.15E-04 | 0.046 | 1.37 | | 240105_at | C21orf66 | chromosome 21 open reading frame 66 | 2.21E-04 | 0.046 | 1.80 | | 233368_s_at | RBJ | rab and DnaJ domain containing | 2.22E-04 | 0.046 | 1.57 | | 222508_s_at | ARGLU1 | arginine and glutamate rich 1 | 2.25E-04 | 0.046 | 1.70 | | 222387_s_at | VPS35 | vacuolar protein sorting 35 homolog (S. cerevisiae) | 2.29E-04 | 0.046 | 1.47 | | 214693_x_at | NBPF1 | neuroblastoma breakpoint family, member 1 | 2.30E-04 | 0.046 | 1.71 | | 224693_at | C20orf108 | chromosome 20 open reading frame 108 | 2.30E-04 | 0.046 | 1.30 | | 207188_at | CDK3 | cyclin-dependent kinase 3 | 2.32E-04 | 0.046 | 1.34 | | 1555765_a_at | GNG4 | guanine nucleotide binding protein (G protein), gamma 4 | 2.47E-04 | 0.048 | 1.38 | | 239002_at | ASPM | asp (abnormal spindle) homolog, microcephaly associated (Drosophila) | 2.50E-04 | 0.048 | 1.70 | | 203833_s_at | TGOLN2 | trans-golgi network protein 2 | 2.58E-04 | 0.049 | 1.63 | | 200607_s_at | RAD21 | RAD21 homolog (S. pombe) | 2.67E-04 | 0.049 | 1.32 | | 210172_at | SF1 | splicing factor 1 | 2.67E-04 | 0.049 | 2.17 | | 229010_at | CBL | Cas-Br-M (murine) ecotropic retroviral transforming sequence | 2.73E-04 | 0.049 | 1.74 | | 233559_s_at | WDFY1 | WD repeat and FYVE domain containing 1 | 2.83E-04 | 0.050 | 1.63 | | 1557910_at | HSP90AB1 | heat shock protein 90kDa alpha (cytosolic), class B member 1 | 2.85E-04 | 0.050 | 1.44 | | 236957_at | CDCA2 | cell division cycle associated 2 | 3.01E-04 | 0.052 | 1.83 | | 211993_at | WNK1 | WNK lysine deficient protein kinase 1 | 3.03E-04 | 0.052 | 1.58 | | 216449_x_at | HSP90B1 | heat shock protein 90kDa beta (Grp94), member 1 | 3.05E-04 | 0.052 | 1.43 | | 212492_s_at | JMJD2B | jumonji domain containing 2B | 3.07E-04 | 0.052 | 1.49 | | 235598_at | SMAD2 | SMAD family member 2 | 3.08E-04 | 0.052 | 1.34 | | 223505_s_at | RBJ | rab and DnaJ domain containing | 3.13E-04 | 0.052 | 1.27 | | 214900_at | ZKSCAN1 | zinc finger with KRAB and SCAN domains 1 | 3.17E-04 | 0.052 | 1.45 | | 227152_at | C12orf35 | chromosome 12 open reading frame 35 | 3.18E-04 | 0.052 | 1.52 | | 214052_x_at | BAT2D1 | BAT2 domain containing 1 | 3.25E-04 | 0.053 | 1.54 | | 57739_at | DND1 | dead end homolog 1 (zebrafish) | 3.47E-04 | 0.054 | 1.22 | | 201793_x_at | SMG7 | Smg-7 homolog, nonsense mediated mRNA decay factor (C. elegans) | 3.49E-04 | 0.054 | 1.35 | | 201085_s_at | SON | SON DNA binding protein | 3.50E-04 | 0.054 | 1.40 | | 208879_x_at | PRPF6 | PRP6 pre-mRNA processing factor 6 homolog (S. cerevisiae) | 3.56E-04 | 0.055 | 1.65 | | 239046_at | TAF1B | TATA box binding protein (TBP)-associated factor, RNA polymerase I, B, 63kDa | 3.65E-04 | 0.055 | 1.57 | | 241419_at | ARHGAP1 | Rho GTPase activating protein 1 | 3.66E-04 | 0.055 | 1.25 | | 222946_s_at | C1orf135 | chromosome 1 open reading frame 135 | 3.67E-04 | 0.055 | 1.33 | | 201615_x_at | CALD1 | caldesmon 1 | 3.72E-04 | 0.055 | 1.50 | | 221734_at | PRRC1 | proline-rich coiled-coil 1 | 3.75E-04 | 0.056 | 1.42 | | 1554249_a_at | ZNF638 | zinc finger protein 638 | 3.83E-04 | 0.056 | 1.64 | | 235266_at | ATAD2 | ATPase family, AAA domain containing 2 | 3.89E-04 | 0.056 | 1.51 | | 214659_x_at | YLPM1 | YLP motif containing 1 | 3.89E-04 | 0.056 | 1.35 | | 222626_at | RBM26 | RNA binding motif protein 26 | 4.03E-04 | 0.056 | 1.43 | | 223940_x_at | MALAT1 | metastasis associated lung adenocarcinoma transcript 1 (non-protein coding) | 4.12E-04 | 0.057 | 2.02 | | 200598_s_at | HSP90B1 | heat shock protein 90kDa beta (Grp94), member 1 | 4.13E-04 | 0.057 | 1.43 | | 201072_s_at | SMARCC1 | SWI/SNF related, matrix associated, actin dependent regulator of chromatin, subfamily c, member 1 | 4.22E-04 | 0.057 | 1.36 | | 205187_at | SMAD5 | SMAD family member 5 | 4.26E-04 | 0.058 | 1.55 | | 226898_s_at | SFPQ | splicing factor proline/glutamine-rich (polypyrimidine tract binding protein associated) | 4.31E-04 | 0.058 | 1.74 | | 201101_s_at | BCLAF1 | BCL2-associated transcription factor 1 | 4.38E-04 | 0.058 | 1.46 | | 220764_at | PPP4R2 | protein phosphatase 4, regulatory subunit 2 | 4.44E-04 | 0.058 | 1.36 | | 201702_s_at | PPP1R10 | protein phosphatase 1, regulatory (inhibitor) subunit 10 | 4.58E-04 | 0.059 | 1.55 | | 227101_at | ZNF800 | zinc finger protein 800 | 4.69E-04 | 0.059 | 1.52 | | 216941_s_at | TAF1B | TATA box binding protein (TBP)-associated factor, RNA polymerase I, B, 63kDa | 4.71E-04 | 0.059 | 1.27 | | 240119_at | TEPP | testis/prostate/placenta-expressed protein | 4.72E-04 | 0.059 | 1.28 | | 226422_at | ERGIC2 | ERGIC and golgi 2 | 4.72E-04 | 0.059 | 1.20 | | 31637_s_at | NR1D1 | nuclear receptor subfamily 1, group D, member 1 | 4.94E-04 | 0.060 | 1.27 | | 230477_at | LOC147299 | hypothetical protein LOC147299 | 4.96E-04 | 0.060 | 1.23 | | 212926_at | SMC5 | structural maintenance of chromosomes 5 | 4.97E-04 | 0.060 | 1.56 | | 223888_s_at | LARS | leucyl-tRNA synthetase | 4.98E-04 | 0.060 | 1.92 | | 213875_x_at | C6orf62 | chromosome 6 open reading frame 62 | 5.08E-04 | 0.061 | 1.23 | | 208178_x_at | TRIO | triple functional domain (PTPRF interacting) | 5.12E-04 | 0.061 | 1.36 | | 90265_at | CENTA1 | centaurin, alpha 1 | 5.16E-04 | 0.061 | 1.24 | | 201208_s_at | TNFAIP1 | tumor necrosis factor, alpha-induced protein 1 (endothelial) | 5.20E-04 | 0.061 | 1.36 | | 201879_at | ARIH1 | ariadne homolog, ubiquitin-conjugating enzyme E2 binding protein, 1 (Drosophila) | 5.25E-04 | 0.061 | 1.39 | | 1554977_at | LOC198437 | bA299N6.3 | 5.26E-04 | 0.061 | 1.43 | | 1552978_a_at | SCAMP1 | secretory carrier membrane protein 1 | 5.27E-04 | 0.061 | 1.63 | | 217826_s_at | UBE2J1 | ubiquitin-conjugating enzyme E2, J1 (UBC6 homolog, yeast) | 5.34E-04 | 0.061 | 1.37 | | 213998_s_at | DDX17 | DEAD (Asp-Glu-Ala-Asp) box polypeptide 17 | 5.38E-04 | 0.061 | 1.78 | | 203056_s_at | PRDM2 | PR domain containing 2, with ZNF domain | 5.40E-04 | 0.061 | 1.81 | | 223577_x_at | MALAT1 | metastasis associated lung adenocarcinoma transcript 1 (non-protein coding) | 5.42E-04 | 0.061 | 1.40 | | 223028_s_at | SNX9 | sorting nexin 9 | 5.48E-04 | 0.061 | 1.38 | | 217644_s_at | SOS2 | son of sevenless homolog 2 (Drosophila) | 5.61E-04 | 0.061 | 1.92 | | 214305_s_at | SF3B1 | splicing factor 3b, subunit 1, 155kDa | 5.62E-04 | 0.061 | 1.61 | | 202323_s_at | ACBD3 | acyl-Coenzyme A binding domain containing 3 | 5.71E-04 | 0.061 | 1.33 | | 223038_s_at | FAM60A | family with sequence similarity 60, member A | 5.71E-04 | 0.061 | 1.23 | | 221016_s_at | TCF7L1 | transcription factor 7-like 1 (T-cell specific, HMG-box) | 5.72E-04 | 0.061 | 1.27 | | 37793_r_at | RAD51L3 | RAD51-like 3 (S. cerevisiae) | 5.74E-04 | 0.061 | 1.39 | | 201730_s_at | TPR | translocated promoter region (to activated MET oncogene) | 5.80E-04 | 0.061 | 1.34 | | 212208_at | MED13L | mediator complex subunit 13-like | 5.83E-04 | 0.061 | 1.33 | | 231272_at | MED7 | mediator complex subunit 7 | 5.84E-04 | 0.061 | 1.45 | | 220219_s_at | LRRC37A3 | leucine rich repeat containing 37, member A3 | 5.91E-04 | 0.061 | 1.27 | | 1554633_a_at | MYT1L | myelin transcription factor 1-like | 5.91E-04 | 0.061 | 1.49 | | 236408_at | KLHDC5 | kelch domain containing 5 | 5.93E-04 | 0.061 | 1.44 | | 223984_s_at | NUPL1 | nucleoporin like 1 | 5.95E-04 | 0.061 | 1.41 | | 200841_s_at | EPRS | glutamyl-prolyl-tRNA synthetase | 5.98E-04 | 0.061 | 1.65 | | 201728_s_at | KIAA0100 | KIAA0100 | 6.03E-04 | 0.061 | 1.39 | | 236665_at | CCDC18 | coiled-coil domain containing 18 | 6.03E-04 | 0.061 | 1.65 | | 215434_x_at | NBPF10 | neuroblastoma breakpoint family, member 10 | 6.14E-04 | 0.061 | 1.70 | | 219769_at | INCENP | inner centromere protein antigens 135/155kDa | 6.17E-04 | 0.061 | 1.37 | | 226173_at | TBC1D25 | TBC1 domain family, member 25 | 6.18E-04 | 0.061 | 1.19 | | 218006_s_at | ZNF22 | zinc finger protein 22 (KOX 15) | 6.23E-04 | 0.061 | 1.28 | | 212758_s_at | ZEB1 | zinc finger E-box binding homeobox 1 | 6.25E-04 | 0.061 | 1.40 | | 235977_at | LONRF2 | LON peptidase N-terminal domain and ring finger 2 | 6.25E-04 | 0.061 | 1.53 | | 1554512_a_at | CCDC123 | coiled-coil domain containing 123 | 6.32E-04 | 0.061 | 1.24 | | 223818_s_at | RSF1 | remodeling and spacing factor 1 | 6.38E-04 | 0.062 | 1.41 | | 242722_at | LMO7 | LIM domain 7 | 6.45E-04 | 0.062 | 2.13 | | 215155_at | HEXA | hexosaminidase A (alpha polypeptide) | 6.45E-04 | 0.062 | 1.25 | | 239270_at | PLCXD3 | phosphatidylinositol-specific phospholipase C, X domain containing 3 | 6.49E-04 | 0.062 | 1.36 | | 211086_x_at | NEK1 | NIMA (never in mitosis gene a)-related kinase 1 | 6.56E-04 | 0.062 | 1.46 | | 218745_x_at | TMEM161A | transmembrane protein 161A | 6.62E-04 | 0.062 | 1.25 | | 200956_s_at | SSRP1 | structure specific recognition protein 1 | 6.63E-04 | 0.062 | 1.28 | | 216962_at | OR5T2 | olfactory receptor, family 5, subfamily T, member 2 | 6.66E-04 | 0.062 | 1.27 | | 1558045_a_at | LOC441528 | hypothetical protein LOC441528 | 6.66E-04 | 0.062 | 1.30 | | 236481_at | FAH | fumarylacetoacetate hydrolase (fumarylacetoacetase) | 6.94E-04 | 0.063 | 1.29 | | 1553672_at | ENAH | enabled homolog (Drosophila) | 6.97E-04 | 0.063 | 1.40 | | 212620_at | ZNF609 | zinc finger protein 609 | 7.12E-04 | 0.064 | 1.30 | | 224311_s_at | CAB39 | calcium binding protein 39 | 7.25E-04 | 0.064 | 1.59 | | 231953_at | BPTF | bromodomain PHD finger transcription factor | 7.34E-04 | 0.064 | 1.37 | | 224563_at | WASF4 | WAS protein family, member 4 | 7.34E-04 | 0.064 | 1.44 | | 208610_s_at | SRRM2 | serine/arginine repetitive matrix 2 | 7.36E-04 | 0.064 | 1.51 | | 214313_s_at | EIF5B | eukaryotic translation initiation factor 5B | 7.41E-04 | 0.064 | 1.47 | | 236254_at | VPS13B | vacuolar protein sorting 13 homolog B (yeast) | 7.42E-04 | 0.064 | 1.36 | | 244110_at | MLL | myeloid/lymphoid or mixed-lineage leukemia (trithorax homolog, Drosophila) | 7.53E-04 | 0.065 | 1.25 | | 226643_s_at | NUDCD2 | NudC domain containing 2 | 7.55E-04 | 0.065 | 1.41 | | 223879_s_at | OXR1 | oxidation resistance 1 | 7.63E-04 | 0.065 | 1.40 | | 228628_at | SRGAP2P1 | SLIT-ROBO Rho GTPase activating protein 2 pseudogene 1 | 7.64E-04 | 0.065 | 1.24 | | 1557227_s_at | TPR | translocated promoter region (to activated MET oncogene) | 7.69E-04 | 0.065 | 2.06 | | 238800_s_at | ZCCHC6 | zinc finger, CCHC domain containing 6 | 7.79E-04 | 0.066 | 1.33 | | 222704_at | POLR1A | polymerase (RNA) I polypeptide A, 194kDa | 7.88E-04 | 0.066 | 1.19 | | 206232_s_at | B4GALT6 | UDP-Gal:betaGlcNAc beta 1,4- galactosyltransferase, polypeptide 6 | 7.90E-04 | 0.066 | 1.58 | | 235833_at | PPAT | phosphoribosyl pyrophosphate amidotransferase | 7.96E-04 | 0.066 | 1.30 | | 209127_s_at | SART3 | squamous cell carcinoma antigen recognized by T cells 3 | 8.02E-04 | 0.066 | 1.25 | | 224854_s_at | SLAIN2 | SLAIN motif family, member 2 | 8.03E-04 | 0.066 | 1.35 | | 201683_x_at | TOX4 | TOX high mobility group box family member 4 | 8.09E-04 | 0.066 | 1.34 | | 1564002_a_at | C6orf199 | chromosome 6 open reading frame 199 | 8.10E-04 | 0.066 | 1.43 | | 1558942_at | ZNF813 | zinc finger protein 813 | 8.16E-04 | 0.066 | 1.28 | | 241820_at | RIF1 | RAP1 interacting factor homolog (yeast) | 8.28E-04 | 0.066 | 1.54 | | 35160_at | LDB1 | LIM domain binding 1 | 8.37E-04 | 0.066 | 1.23 | | 237741_at | SLC25A36 | solute carrier family 25, member 36 | 8.59E-04 | 0.067 | 1.67 | | 209013_x_at | TRIO | triple functional domain (PTPRF interacting) | 8.66E-04 | 0.068 | 1.31 | | 213963_s_at | SAP30 | Sin3A-associated protein, 30kDa | 8.74E-04 | 0.068 | 1.25 | | 201294_s_at | WSB1 | WD repeat and SOCS box-containing 1 | 8.82E-04 | 0.068 | 1.45 | | 242135_at | LOC642236 | similar to FRG1 protein (FSHD region gene 1 protein) | 8.90E-04 | 0.069 | 1.26 | | 242609_x_at | ADCK4 | aarF domain containing kinase 4 | 8.97E-04 | 0.069 | 1.43 | | 1554873_at | CSPP1 | centrosome and spindle pole associated protein 1 | 8.98E-04 | 0.069 | 1.42 | | 220223_at | ATAD5 | ATPase family, AAA domain containing 5 | 9.01E-04 | 0.069 | 1.30 | | 235376_at | USP4 | ubiquitin specific peptidase 4 (proto-oncogene) | 9.08E-04 | 0.069 | 1.63 | | 229081_at | SLC25A13 | solute carrier family 25, member 13 (citrin) | 9.08E-04 | 0.069 | 1.29 | | 232127_at | CLCN5 | chloride channel 5 (nephrolithiasis 2, X-linked, Dent disease) | 9.08E-04 | 0.069 | 1.52 | | 230180_at | DDX17 | DEAD (Asp-Glu-Ala-Asp) box polypeptide 17 | 9.16E-04 | 0.069 | 2.58 | | 1560599_a_at | CCDC123 | coiled-coil domain containing 123 | 9.19E-04 | 0.069 | 1.41 | | 244224_x_at | LYNX1 | Ly6/neurotoxin 1 | 9.36E-04 | 0.069 | 1.36 | | 208503_s_at | GATAD1 | GATA zinc finger domain containing 1 | 9.60E-04 | 0.070 | 1.20 | | 230000_at | RNF213 | ring finger protein 213 | 9.63E-04 | 0.070 | 1.39 | | 218521_s_at | UBE2W | ubiquitin-conjugating enzyme E2W (putative) | 9.75E-04 | 0.070 | 1.39 | | 210461_s_at | ABLIM1 | actin binding LIM protein 1 | 9.76E-04 | 0.070 | 1.47 | | 1559108_at | VPS53 | vacuolar protein sorting 53 homolog (S. cerevisiae) | 9.80E-04 | 0.071 | 1.50 | | 219393_s_at | AKT3 | v-akt murine thymoma viral oncogene homolog 3 (protein kinase B, gamma) | 9.85E-04 | 0.071 | 1.54 | | 226365_at | LOC728555 | hypothetical LOC728555 | 9.93E-04 | 0.071 | 1.36 | | 220794_at | GREM2 | gremlin 2, cysteine knot superfamily, homolog (Xenopus laevis) | 9.94E-04 | 0.071 | 1.46 | | 221960_s_at | RAB2A | RAB2A, member RAS oncogene family | 9.95E-04 | 0.071 | 1.71 | | 206578_at | NKX2-5 | NK2 transcription factor related, locus 5 (Drosophila) | 9.97E-04 | 0.071 | 1.35 | | 221976_s_at | HDGFRP3 | hepatoma-derived growth factor, related protein 3 | 1.01E-03 | 0.071 | 1.68 | | 224853_at | SLAIN2 | SLAIN motif family, member 2 | 1.02E-03 | 0.071 | 1.38 | | 222850_s_at | DNAJB14 | DnaJ (Hsp40) homolog, subfamily B, member 14 | 1.03E-03 | 0.071 | 1.33 | | 1554084_a_at | NOL9 | nucleolar protein 9 | 1.03E-03 | 0.071 | 1.34 | | 212382_at | TCF4 | transcription factor 4 | 1.04E-03 | 0.071 | 1.34 | | 223134_at | BBX | bobby sox homolog (Drosophila) | 1.07E-03 | 0.073 | 1.24 | | 201711_x_at | RANBP2 | RAN binding protein 2 | 1.09E-03 | 0.074 | 1.31 | | 1557388_at | RTTN | rotatin | 1.10E-03 | 0.075 | 1.42 | | 226919_at | LYRM2 | LYR motif containing 2 | 1.11E-03 | 0.075 | 1.33 | | 208900_s_at | TOP1 | topoisomerase (DNA) I | 1.11E-03 | 0.075 | 1.48 | | 225476_at | BAT4 | HLA-B associated transcript 4 | 1.12E-03 | 0.075 | 1.27 | | 224227_s_at | BDP1 | B double prime 1, subunit of RNA polymerase III transcription initiation factor IIIB | 1.13E-03 | 0.076 | 1.82 | | 203589_s_at | TFDP2 | transcription factor Dp-2 (E2F dimerization partner 2) | 1.16E-03 | 0.076 | 1.23 | | 201679_at | ARS2 | arsenate resistance protein 2 | 1.17E-03 | 0.076 | 1.29 | | 227171_at | CCT4 | chaperonin containing TCP1, subunit 4 (delta) | 1.18E-03 | 0.076 | 1.38 | | 216493_s_at | IGF2BP3 | insulin-like growth factor 2 mRNA binding protein 3 | 1.18E-03 | 0.076 | 1.79 | | 242635_s_at | NAPEPLD | N-acyl phosphatidylethanolamine phospholipase D | 1.19E-03 | 0.077 | 1.23 | | 1558046_x_at | LOC441528 | hypothetical protein LOC441528 | 1.22E-03 | 0.078 | 1.26 | | 244486_at | PINK1 | PTEN induced putative kinase 1 | 1.22E-03 | 0.078 | 1.25 | | 217777_s_at | PTPLAD1 | protein tyrosine phosphatase-like A domain containing 1 | 1.23E-03 | 0.078 | 1.39 | | 219858_s_at | FLJ20160 | FLJ20160 protein | 1.23E-03 | 0.078 | 1.36 | | 219998_at | HSPC159 | galectin-related protein | 1.23E-03 | 0.078 | 1.36 | | 212763_at | CAMSAP1L1 | calmodulin regulated spectrin-associated protein 1-like 1 | 1.24E-03 | 0.078 | 1.60 | | 228563_at | GJC1 | gap junction protein, gamma 1, 45kDa | 1.24E-03 | 0.078 | 1.32 | | 1555360_a_at | DNAJC11 | DnaJ (Hsp40) homolog, subfamily C, member 11 | 1.25E-03 | 0.078 | 1.39 | | 201446_s_at | TIA1 | TIA1 cytotoxic granule-associated RNA binding protein | 1.25E-03 | 0.078 | 1.32 | | 213623_at | KIF3A | kinesin family member 3A | 1.26E-03 | 0.078 | 1.19 | | 206668_s_at | SCAMP1 | secretory carrier membrane protein 1 | 1.26E-03 | 0.078 | 1.36 | | 205787_x_at | RP11-74E24.2 | zinc finger CCCH-type domain-containing pseudogene | 1.27E-03 | 0.078 | 1.76 | | 222463_s_at | BACE1 | beta-site APP-cleaving enzyme 1 | 1.29E-03 | 0.079 | 1.33 | | 1559808_at | PLEC1 | plectin 1, intermediate filament binding protein 500kDa | 1.29E-03 | 0.079 | 1.28 | | 224517_at | POLR2J4 | polymerase (RNA) II (DNA directed) polypeptide J, 13.3kDa pseudogene | 1.29E-03 | 0.079 | 1.51 | | 230546_at | VASH1 | vasohibin 1 | 1.30E-03 | 0.079 | 1.35 | | 224335_s_at | BACE1 | beta-site APP-cleaving enzyme 1 | 1.30E-03 | 0.079 | 1.57 | | 230071_at | SEPT11 | septin 11 | 1.32E-03 | 0.079 | 1.34 | | 221279_at | GDAP1 | ganglioside-induced differentiation-associated protein 1 | 1.34E-03 | 0.079 | 1.44 | | 232218_at | C9orf119 | chromosome 9 open reading frame 119 | 1.34E-03 | 0.079 | 1.20 | | 220295_x_at | DEPDC1 | DEP domain containing 1 | 1.34E-03 | 0.079 | 1.71 | | 209669_s_at | SERBP1 | SERPINE1 mRNA binding protein 1 | 1.34E-03 | 0.079 | 1.18 | | 218554_s_at | ASH1L | ash1 (absent, small, or homeotic)-like (Drosophila) | 1.35E-03 | 0.079 | 1.31 | | 236769_at | LOC158402 | hypothetical protein LOC158402 | 1.35E-03 | 0.079 | 1.27 | | 209136_s_at | USP10 | ubiquitin specific peptidase 10 | 1.35E-03 | 0.079 | 1.60 | | 201070_x_at | SF3B1 | splicing factor 3b, subunit 1, 155kDa | 1.35E-03 | 0.079 | 1.56 | | 222404_x_at | PTPLAD1 | protein tyrosine phosphatase-like A domain containing 1 | 1.35E-03 | 0.079 | 1.18 | | 219158_s_at | NARG1 | NMDA receptor regulated 1 | 1.36E-03 | 0.079 | 1.34 | | 214955_at | TMPRSS6 | transmembrane protease, serine 6 | 1.38E-03 | 0.079 | 1.31 | | 1557370_s_at | MYCBP2 | MYC binding protein 2 | 1.40E-03 | 0.079 | 1.30 | | 239833_at | COMMD1 | copper metabolism (Murr1) domain containing 1 | 1.40E-03 | 0.079 | 1.54 | | 204121_at | GADD45G | growth arrest and DNA-damage-inducible, gamma | 1.40E-03 | 0.079 | 1.28 | | 1558924_s_at | CLIP1 | CAP-GLY domain containing linker protein 1 | 1.42E-03 | 0.079 | 1.73 | | 225640_at | LOC401504 | hypothetical gene supported by AK091718 | 1.42E-03 | 0.079 | 1.26 | | 235003_at | UHMK1 | U2AF homology motif (UHM) kinase 1 | 1.42E-03 | 0.079 | 1.83 | | 238966_at | BRUNOL4 | bruno-like 4, RNA binding protein (Drosophila) | 1.43E-03 | 0.080 | 1.31 | | 207661_s_at | SH3PXD2A | SH3 and PX domains 2A | 1.44E-03 | 0.080 | 1.20 | | 233080_s_at | PRPF40A | PRP40 pre-mRNA processing factor 40 homolog A (S. cerevisiae) | 1.45E-03 | 0.080 | 1.28 | | 209920_at | BMPR2 | bone morphogenetic protein receptor, type II (serine/threonine kinase) | 1.45E-03 | 0.080 | 1.37 | | 229570_at | LAMA5 | laminin, alpha 5 | 1.45E-03 | 0.080 | 1.25 | | 219024_at | PLEKHA1 | pleckstrin homology domain containing, family A (phosphoinositide binding specific) member 1 | 1.46E-03 | 0.080 | 1.32 | | 216554_s_at | MBNL1 | muscleblind-like (Drosophila) | 1.50E-03 | 0.081 | 1.44 | | 221052_at | TDRKH | tudor and KH domain containing | 1.50E-03 | 0.081 | 1.25 | | 205809_s_at | WASL | Wiskott-Aldrich syndrome-like | 1.51E-03 | 0.081 | 1.60 | | 1556222_at | RP11-291L22.2 | similar to cell division cycle 10 | 1.51E-03 | 0.081 | 1.34 | | 200907_s_at | PALLD | palladin, cytoskeletal associated protein | 1.54E-03 | 0.082 | 1.28 | | 205188_s_at | SMAD5 | SMAD family member 5 | 1.55E-03 | 0.082 | 1.77 | | 213119_at | SLC36A1 | solute carrier family 36 (proton/amino acid symporter), member 1 | 1.59E-03 | 0.083 | 1.20 | | 1569472_s_at | TTC3 | tetratricopeptide repeat domain 3 | 1.60E-03 | 0.083 | 1.79 | | 1553106_at | C5orf24 | chromosome 5 open reading frame 24 | 1.62E-03 | 0.083 | 1.93 | | 219507_at | RSRC1 | arginine/serine-rich coiled-coil 1 | 1.62E-03 | 0.083 | 1.41 | | 221276_s_at | SYNC1 | syncoilin, intermediate filament 1 | 1.63E-03 | 0.083 | 1.23 | | 1552274_at | PXK | PX domain containing serine/threonine kinase | 1.64E-03 | 0.083 | 1.37 | | 241955_at | HECTD1 | HECT domain containing 1 | 1.64E-03 | 0.083 | 1.62 | | 1555125_at | C21orf66 | chromosome 21 open reading frame 66 | 1.65E-03 | 0.083 | 1.39 | | 202028_s_at | RPL38 | ribosomal protein L38 | 1.65E-03 | 0.083 | 1.32 | | 1555272_at | LOC728194 | radial spoke head 10 homolog B (Chlamydomonas)-like | 1.66E-03 | 0.083 | 1.31 | | 219576_at | MAP7D3 | MAP7 domain containing 3 | 1.66E-03 | 0.083 | 1.25 | | 1563321_s_at | MLLT10 | myeloid/lymphoid or mixed-lineage leukemia (trithorax homolog, Drosophila); translocated to, 10 | 1.67E-03 | 0.083 | 1.40 | | 202132_at | WWTR1 | WW domain containing transcription regulator 1 | 1.68E-03 | 0.083 | 1.37 | | 219387_at | CCDC88A | coiled-coil domain containing 88A | 1.68E-03 | 0.083 | 1.48 | | 212332_at | RBL2 | retinoblastoma-like 2 (p130) | 1.69E-03 | 0.083 | 1.43 | | 1552812_a_at | SENP1 | SUMO1/sentrin specific peptidase 1 | 1.70E-03 | 0.083 | 1.41 | | 242762_s_at | KIAA1946 | KIAA1946 | 1.73E-03 | 0.084 | 1.40 | | 223222_at | SLC25A19 | solute carrier family 25 (mitochondrial thiamine pyrophosphate carrier), member 19 | 1.75E-03 | 0.084 | 1.18 | | 227918_s_at | ZYG11B | zyg-11 homolog B (C. elegans) | 1.75E-03 | 0.084 | 1.39 | | 208003_s_at | NFAT5 | nuclear factor of activated T-cells 5, tonicity-responsive | 1.77E-03 | 0.085 | 1.56 | | 238831_at | TMEM33 | transmembrane protein 33 | 1.78E-03 | 0.085 | 1.68 | | 203784_s_at | DDX28 | DEAD (Asp-Glu-Ala-Asp) box polypeptide 28 | 1.79E-03 | 0.085 | 1.29 | | 1567906_at | SOX4 | SRY (sex determining region Y)-box 4 | 1.79E-03 | 0.085 | 1.34 | | 1558251_a_at | ZNF587 | zinc finger protein 587 | 1.81E-03 | 0.085 | 1.21 | | 216997_x_at | TLE4 | transducin-like enhancer of split 4 (E(sp1) homolog, Drosophila) | 1.82E-03 | 0.085 | 1.59 | | 206667_s_at | SCAMP1 | secretory carrier membrane protein 1 | 1.82E-03 | 0.085 | 1.71 | | 1557595_at | GINS2 | GINS complex subunit 2 (Psf2 homolog) | 1.84E-03 | 0.085 | 1.24 | | 225965_at | DDHD1 | DDHD domain containing 1 | 1.84E-03 | 0.085 | 1.41 | | 1558641_at | LOC202051 | hypothetical protein LOC202051 | 1.85E-03 | 0.085 | 1.22 | | 206538_at | MRAS | muscle RAS oncogene homolog | 1.85E-03 | 0.085 | 1.24 | | 236506_at | NIN | ninein (GSK3B interacting protein) | 1.87E-03 | 0.085 | 1.29 | | 208744_x_at | HSPH1 | heat shock 105kDa/110kDa protein 1 | 1.87E-03 | 0.085 | 1.47 | | 224589_at | XIST | X (inactive)-specific transcript (non-protein coding) | 1.88E-03 | 0.085 | 1.35 | | 1554159_a_at | ZMYND11 | zinc finger, MYND domain containing 11 | 1.88E-03 | 0.085 | 1.53 | | 219717_at | C4orf30 | chromosome 4 open reading frame 30 | 1.89E-03 | 0.085 | 1.41 | | 223254_s_at | KIAA1333 | KIAA1333 | 1.89E-03 | 0.085 | 1.49 | | 1553107_s_at | C5orf24 | chromosome 5 open reading frame 24 | 1.89E-03 | 0.085 | 1.24 | | 241611_s_at | FNDC3A | fibronectin type III domain containing 3A | 1.90E-03 | 0.085 | 1.58 | | 228067_at | C2orf55 | chromosome 2 open reading frame 55 | 1.91E-03 | 0.085 | 1.27 | | 225852_at | ANKRD17 | ankyrin repeat domain 17 | 1.94E-03 | 0.086 | 1.26 | | 1567213_at | PNN | pinin, desmosome associated protein | 1.94E-03 | 0.086 | 1.40 | | 235653_s_at | THAP6 | THAP domain containing 6 | 1.95E-03 | 0.086 | 1.48 | | 226967_at | FIZ1 | FLT3-interacting zinc finger 1 | 1.97E-03 | 0.086 | 1.24 | | 203348_s_at | ETV5 | ets variant gene 5 (ets-related molecule) | 1.99E-03 | 0.087 | 1.20 | | 210214_s_at | BMPR2 | bone morphogenetic protein receptor, type II (serine/threonine kinase) | 1.99E-03 | 0.087 | 1.40 | | 238474_at | NUP43 | nucleoporin 43kDa | 1.99E-03 | 0.087 | 1.38 | | 221524_s_at | RRAGD | Ras-related GTP binding D | 2.00E-03 | 0.087 | 1.24 | | 214451_at | TFAP2B | transcription factor AP-2 beta (activating enhancer binding protein 2 beta) | 2.00E-03 | 0.087 | 1.19 | | 228673_s_at | EML4 | echinoderm microtubule associated protein like 4 | 2.00E-03 | 0.087 | 1.63 | | 224631_at | ZFP91 | zinc finger protein 91 homolog (mouse) | 2.01E-03 | 0.087 | 1.21 | | 242787_at | INCENP | inner centromere protein antigens 135/155kDa | 2.03E-03 | 0.087 | 1.18 | | 212808_at | NFATC2IP | nuclear factor of activated T-cells, cytoplasmic, calcineurin-dependent 2 interacting protein | 2.05E-03 | 0.088 | 1.38 | | 1557385_at | FLJ13305 | hypothetical protein FLJ13305 | 2.06E-03 | 0.088 | 1.36 | | 216609_at | TXN | thioredoxin | 2.09E-03 | 0.088 | 1.23 | | 213926_s_at | HRB | HIV-1 Rev binding protein | 2.10E-03 | 0.088 | 1.57 | | 228688_at | FANCF | Fanconi anemia, complementation group F | 2.13E-03 | 0.089 | 1.33 | | 202044_at | GRLF1 | glucocorticoid receptor DNA binding factor 1 | 2.15E-03 | 0.089 | 1.17 | | 203609_s_at | ALDH5A1 | aldehyde dehydrogenase 5 family, member A1 (succinate-semialdehyde dehydrogenase) | 2.16E-03 | 0.089 | 1.42 | | 239202_at | RAB3B | RAB3B, member RAS oncogene family | 2.16E-03 | 0.089 | 1.33 | | 208629_s_at | HADHA | hydroxyacyl-Coenzyme A dehydrogenase/3-ketoacyl-Coenzyme A thiolase/enoyl-Coenzyme A hydratase (trifunctional protein), alpha subunit | 2.17E-03 | 0.089 | 1.25 | | 235173_at | MBNL1 | muscleblind-like (Drosophila) | 2.17E-03 | 0.089 | 1.24 | | 228250_at | FNIP1 | folliculin interacting protein 1 | 2.18E-03 | 0.089 | 1.40 | | 201723_s_at | GALNT1 | UDP-N-acetyl-alpha-D-galactosamine:polypeptide N-acetylgalactosaminyltransferase 1 (GalNAc-T1) | 2.20E-03 | 0.089 | 1.26 | | 238608_at | LAMB1 | laminin, beta 1 | 2.21E-03 | 0.089 | 1.33 | | 206536_s_at | XIAP | X-linked inhibitor of apoptosis | 2.22E-03 | 0.089 | 1.54 | | 211580_s_at | PIK3R3 | phosphoinositide-3-kinase, regulatory subunit 3 (gamma) | 2.23E-03 | 0.089 | 1.32 | | 231999_at | ANKRD11 | ankyrin repeat domain 11 | 2.23E-03 | 0.089 | 1.27 | | 206500_s_at | C14orf106 | chromosome 14 open reading frame 106 | 2.24E-03 | 0.089 | 1.76 | | 213189_at | DKFZp667G2110 | hypothetical protein DKFZp667G2110 | 2.29E-03 | 0.090 | 1.21 | | 232913_at | TMED8 | transmembrane emp24 protein transport domain containing 8 | 2.32E-03 | 0.091 | 1.36 | | 211085_s_at | STK4 | serine/threonine kinase 4 | 2.33E-03 | 0.091 | 1.47 | | 243132_at | APTX | aprataxin | 2.34E-03 | 0.091 | 1.24 | | 1555892_s_at | LOC253039 | hypothetical protein LOC253039 | 2.35E-03 | 0.091 | 1.30 | | 213826_s_at | H3F3A | H3 histone, family 3A | 2.36E-03 | 0.091 | 1.31 | | 213763_at | HIPK2 | homeodomain interacting protein kinase 2 | 2.38E-03 | 0.092 | 1.37 | | 212374_at | FEM1B | fem-1 homolog b (C. elegans) | 2.41E-03 | 0.092 | 1.25 | | 204183_s_at | ADRBK2 | adrenergic, beta, receptor kinase 2 | 2.46E-03 | 0.094 | 1.21 | | 1568877_a_at | ACBD5 | acyl-Coenzyme A binding domain containing 5 | 2.48E-03 | 0.094 | 1.48 | | 241702_at | HNRPD | heterogeneous nuclear ribonucleoprotein D (AU-rich element RNA binding protein 1, 37kDa) | 2.50E-03 | 0.095 | 1.57 | | 201183_s_at | CHD4 | chromodomain helicase DNA binding protein 4 | 2.51E-03 | 0.095 | 1.19 | | 214941_s_at | PRPF40A | PRP40 pre-mRNA processing factor 40 homolog A (S. cerevisiae) | 2.51E-03 | 0.095 | 1.17 | | 218210_at | FN3KRP | fructosamine-3-kinase-related protein | 2.52E-03 | 0.095 | 1.16 | | 239978_at | PITPNB | phosphatidylinositol transfer protein, beta | 2.52E-03 | 0.095 | 1.53 | | 235551_at | WDR4 | WD repeat domain 4 | 2.55E-03 | 0.095 | 1.36 | | 236613_at | RBM25 | RNA binding motif protein 25 | 2.55E-03 | 0.095 | 1.37 | | 232013_at | C9orf102 | chromosome 9 open reading frame 102 | 2.58E-03 | 0.096 | 1.49 | | 236557_at | ZBTB38 | zinc finger and BTB domain containing 38 | 2.58E-03 | 0.096 | 1.31 | | 221683_s_at | CEP290 | centrosomal protein 290kDa | 2.59E-03 | 0.096 | 1.31 | | 209257_s_at | SMC3 | structural maintenance of chromosomes 3 | 2.60E-03 | 0.096 | 1.23 | | 213957_s_at | CEP350 | centrosomal protein 350kDa | 2.62E-03 | 0.096 | 1.55 | | 201593_s_at | ZC3H15 | zinc finger CCCH-type containing 15 | 2.62E-03 | 0.096 | 1.15 | | 212007_at | UBXD2 | UBX domain containing 2 | 2.64E-03 | 0.096 | 1.34 | | 229571_at | CALM2 | calmodulin 2 (phosphorylase kinase, delta) | 2.64E-03 | 0.096 | 1.46 | | 204999_s_at | ATF5 | activating transcription factor 5 | 2.65E-03 | 0.096 | 1.25 | | 242828_at | FIGN | fidgetin | 2.66E-03 | 0.096 | 1.22 | | 241871_at | CAMK4 | calcium/calmodulin-dependent protein kinase IV | 2.69E-03 | 0.097 | 1.28 | | 231011_at | LARP2 | La ribonucleoprotein domain family, member 2 | 2.69E-03 | 0.097 | 1.42 | | 206240_s_at | ZNF136 | zinc finger protein 136 | 2.72E-03 | 0.097 | 1.20 | | 211871_x_at | GNB5 | guanine nucleotide binding protein (G protein), beta 5 | 2.72E-03 | 0.097 | 1.39 | | 1553755_at | NXNL1 | nucleoredoxin-like 1 | 2.74E-03 | 0.097 | 1.36 | | 221705_s_at | SIKE | suppressor of IKK epsilon | 2.74E-03 | 0.097 | 1.74 | | 202631_s_at | APPBP2 | amyloid beta precursor protein (cytoplasmic tail) binding protein 2 | 2.74E-03 | 0.097 | 1.30 | | 217094_s_at | ITCH | itchy E3 ubiquitin protein ligase homolog (mouse) | 2.75E-03 | 0.097 | 1.18 | | 204047_s_at | PHACTR2 | phosphatase and actin regulator 2 | 2.75E-03 | 0.097 | 1.53 | | 230226_s_at | JARID1A | jumonji, AT rich interactive domain 1A | 2.76E-03 | 0.097 | 1.33 | | 1554907_a_at | HYDIN | hydrocephalus inducing homolog (mouse) | 2.77E-03 | 0.097 | 1.21 | | 204077_x_at | ENTPD4 | ectonucleoside triphosphate diphosphohydrolase 4 | 2.79E-03 | 0.097 | 1.29 | | 201914_s_at | SEC63 | SEC63 homolog (S. cerevisiae) | 2.81E-03 | 0.097 | 1.18 | | 216804_s_at | PDLIM5 | PDZ and LIM domain 5 | 2.81E-03 | 0.097 | 1.38 | | 214248_s_at | TRIM2 | tripartite motif-containing 2 | 2.81E-03 | 0.097 | 1.27 | | 230443_at | NHP2L1 | NHP2 non-histone chromosome protein 2-like 1 (S. cerevisiae) | 2.83E-03 | 0.097 | 1.29 | | 221765_at | UGCG | UDP-glucose ceramide glucosyltransferase | 2.84E-03 | 0.097 | 1.81 | | 243857_at | MORF4L2 | mortality factor 4 like 2 | 2.84E-03 | 0.097 | 1.63 | | 217560_at | GGA1 | golgi associated, gamma adaptin ear containing, ARF binding protein 1 | 2.91E-03 | 0.098 | 1.29 | | 230887_at | CDC14B | CDC14 cell division cycle 14 homolog B (S. cerevisiae) | 2.91E-03 | 0.098 | 1.29 | | 236108_at | KIAA1632 | KIAA1632 | 2.93E-03 | 0.098 | 1.27 | | 215682_at | LOC440792 | proline dehydrogenase (oxidase) 1 pseudogene | 2.94E-03 | 0.098 | 1.18 | | 224775_at | IWS1 | IWS1 homolog (S. cerevisiae) | 2.95E-03 | 0.098 | 1.50 | | 223653_x_at | BRUNOL4 | bruno-like 4, RNA binding protein (Drosophila) | 2.95E-03 | 0.098 | 1.39 | | 1552680_a_at | CASC5 | cancer susceptibility candidate 5 | 2.96E-03 | 0.098 | 1.40 | | 218211_s_at | MLPH | melanophilin | 2.96E-03 | 0.098 | 1.28 | | 1564467_at | FLJ13305 | hypothetical protein FLJ13305 | 2.96E-03 | 0.098 | 1.26 | | 1557128_at | FAM111B | family with sequence similarity 111, member B | 2.97E-03 | 0.098 | 1.86 | | 203653_s_at | COIL | coilin | 2.97E-03 | 0.098 | 1.33 | | 242349_at | HECTD1 | HECT domain containing 1 | 2.99E-03 | 0.098 | 1.55 | | 1568680_s_at | YTHDC2 | YTH domain containing 2 | 3.00E-03 | 0.098 | 1.44 | | 1553252_a_at | BRWD3 | bromodomain and WD repeat domain containing 3 | 3.01E-03 | 0.098 | 1.73 | | 211515_s_at | RIPK5 | receptor interacting protein kinase 5 | 3.01E-03 | 0.098 | 1.38 | | 217234_s_at | EZR | ezrin | 3.01E-03 | 0.098 | 1.34 | | 230313_at | TK2 | thymidine kinase 2, mitochondrial | 3.02E-03 | 0.098 | 1.32 | | 212307_s_at | OGT | O-linked N-acetylglucosamine (GlcNAc) transferase (UDP-N-acetylglucosamine:polypeptide-N-acetylglucosaminyl transferase) | 3.04E-03 | 0.098 | 1.49 | | 1558533_at | KRBA2 | KRAB-A domain containing 2 | 3.04E-03 | 0.098 | 1.17 | | 236251_at | ITGAV | integrin, alpha V (vitronectin receptor, alpha polypeptide, antigen CD51) | 3.05E-03 | 0.098 | 1.66 | | 210239_at | IRX5 | iroquois homeobox 5 | 3.06E-03 | 0.099 | 1.38 | | 209024_s_at | SYNCRIP | synaptotagmin binding, cytoplasmic RNA interacting protein | 3.08E-03 | 0.099 | 1.40 | | 244055_at | RYR3 | ryanodine receptor 3 | 3.09E-03 | 0.099 | 1.37 | | 219066_at | PPCDC | phosphopantothenoylcysteine decarboxylase | 3.13E-03 | 0.099 | 1.20 | |  |  |  |  |  |  | | **Down-regulated** | |  |  |  |  | |  |  |  |  |  |  | | 215778_x_at | HAB1 | B1 for mucin | 1.05E-07 | 0.003 | -1.88 | | 216289_at | GPR144 | G protein-coupled receptor 144 | 2.29E-06 | 0.014 | -1.51 | | 214316_x_at | CALR | calreticulin | 6.58E-06 | 0.014 | -1.76 | | 238199_x_at | LOC440552 | OK/SW-cl.16 | 7.43E-06 | 0.014 | -1.90 | | 244656_at | RASL10B | RAS-like, family 10, member B | 2.32E-05 | 0.021 | -1.76 | | 229561_at | LRRC16B | leucine rich repeat containing 16B | 2.38E-05 | 0.021 | -1.43 | | 213177_at | MAPK8IP3 | mitogen-activated protein kinase 8 interacting protein 3 | 2.51E-05 | 0.021 | -1.44 | | 239446_x_at | DCBLD2 | discoidin, CUB and LCCL domain containing 2 | 2.69E-05 | 0.022 | -1.95 | | 240686_x_at | TFRC | transferrin receptor (p90, CD71) | 3.29E-05 | 0.024 | -1.62 | | 213754_s_at | PAIP1 | poly(A) binding protein interacting protein 1 | 3.51E-05 | 0.025 | -1.37 | | 230929_s_at | UBE2J2 | ubiquitin-conjugating enzyme E2, J2 (UBC6 homolog, yeast) | 3.82E-05 | 0.026 | -1.63 | | 208730_x_at | RAB2A | RAB2A, member RAS oncogene family | 3.98E-05 | 0.026 | -1.43 | | 214174_s_at | PDLIM4 | PDZ and LIM domain 4 | 4.85E-05 | 0.028 | -1.39 | | 225587_at | TMEM129 | transmembrane protein 129 | 5.22E-05 | 0.029 | -1.36 | | 229747_x_at | MGC40489 | hypothetical protein MGC40489 | 5.62E-05 | 0.030 | -1.71 | | 210782_x_at | GRIN1 | glutamate receptor, ionotropic, N-methyl D-aspartate 1 | 6.01E-05 | 0.030 | -1.40 | | 207914_x_at | EVX1 | even-skipped homeobox 1 | 6.10E-05 | 0.030 | -1.41 | | 221890_at | ZNF335 | zinc finger protein 335 | 6.62E-05 | 0.030 | -1.25 | | 235186_at | LOC388692 | hypothetical gene supported by AK123662 | 6.75E-05 | 0.030 | -1.49 | | 200639_s_at | YWHAZ | tyrosine 3-monooxygenase/tryptophan 5-monooxygenase activation protein, zeta polypeptide | 8.24E-05 | 0.032 | -1.38 | | 209648_x_at | SOCS5 | suppressor of cytokine signaling 5 | 9.05E-05 | 0.035 | -1.44 | | 214403_x_at | SPDEF | SAM pointed domain containing ets transcription factor | 1.07E-04 | 0.037 | -1.35 | | 231846_at | FOXRED2 | FAD-dependent oxidoreductase domain containing 2 | 1.14E-04 | 0.037 | -1.33 | | 227738_s_at | ARMC5 | armadillo repeat containing 5 | 1.20E-04 | 0.038 | -1.39 | | 241346_at | ARHGAP30 | Rho GTPase activating protein 30 | 1.26E-04 | 0.039 | -1.29 | | 213225_at | PPM1B | protein phosphatase 1B (formerly 2C), magnesium-dependent, beta isoform | 1.33E-04 | 0.041 | -1.37 | | 212925_at | C19orf21 | chromosome 19 open reading frame 21 | 1.38E-04 | 0.041 | -1.35 | | 208420_x_at | SUPT6H | suppressor of Ty 6 homolog (S. cerevisiae) | 1.47E-04 | 0.041 | -1.30 | | 223851_s_at | TNFRSF18 | tumor necrosis factor receptor superfamily, member 18 | 1.48E-04 | 0.041 | -1.35 | | 33760_at | PEX14 | peroxisomal biogenesis factor 14 | 1.50E-04 | 0.041 | -1.27 | | 203852_s_at | SMN1 | survival of motor neuron 1, telomeric | 1.53E-04 | 0.041 | -1.51 | | 227781_x_at | FAM57B | family with sequence similarity 57, member B | 1.60E-04 | 0.041 | -1.45 | | 220561_at | IGF2AS | insulin-like growth factor 2 antisense | 1.72E-04 | 0.042 | -1.46 | | 232683_s_at | PARP6 | poly (ADP-ribose) polymerase family, member 6 | 1.75E-04 | 0.042 | -1.24 | | 203488_at | LPHN1 | latrophilin 1 | 1.76E-04 | 0.042 | -1.23 | | 231268_at | LOC645895 | hypothetical LOC645895 | 1.92E-04 | 0.044 | -1.48 | | 201783_s_at | RELA | v-rel reticuloendotheliosis viral oncogene homolog A, nuclear factor of kappa light polypeptide gene enhancer in B-cells 3, p65 (avian) | 1.99E-04 | 0.044 | -1.23 | | 241669_x_at | PRKD2 | protein kinase D2 | 2.08E-04 | 0.045 | -1.81 | | 221869_at | ZNF512B | zinc finger protein 512B | 2.09E-04 | 0.045 | -1.42 | | 221136_at | GDF2 | growth differentiation factor 2 | 2.09E-04 | 0.045 | -1.44 | | 225229_at | AFF4 | AF4/FMR2 family, member 4 | 2.20E-04 | 0.046 | -1.27 | | 235195_at | FBXW2 | F-box and WD repeat domain containing 2 | 2.42E-04 | 0.048 | -1.34 | | 221141_x_at | EPN1 | epsin 1 | 2.48E-04 | 0.048 | -1.29 | | 228744_at | CEP27 | centrosomal protein 27kDa | 2.65E-04 | 0.049 | -1.38 | | 209767_s_at | sep-05 | septin 5 | 2.70E-04 | 0.049 | -1.30 | | 220173_at | C14orf45 | chromosome 14 open reading frame 45 | 2.70E-04 | 0.049 | -1.37 | | 230073_at | DR1 | down-regulator of transcription 1, TBP-binding (negative cofactor 2) | 2.73E-04 | 0.049 | -1.46 | | 227304_at | SMCR8 | Smith-Magenis syndrome chromosome region, candidate 8 | 2.79E-04 | 0.050 | -1.28 | | 212450_at | KIAA0256 | KIAA0256 gene product | 3.05E-04 | 0.052 | -1.38 | | 216180_s_at | SYNJ2 | synaptojanin 2 | 3.07E-04 | 0.052 | -1.30 | | 222244_s_at | TUG1 | taurine upregulated gene 1 | 3.23E-04 | 0.053 | -1.27 | | 218169_at | VAC14 | Vac14 homolog (S. cerevisiae) | 3.29E-04 | 0.053 | -1.24 | | 233734_s_at | OSBPL5 | oxysterol binding protein-like 5 | 3.32E-04 | 0.053 | -1.39 | | 212553_at | KIAA0460 | KIAA0460 | 3.32E-04 | 0.053 | -1.29 | | 223390_at | C9orf37 | chromosome 9 open reading frame 37 | 3.49E-04 | 0.054 | -1.27 | | 204239_s_at | NNAT | neuronatin | 3.61E-04 | 0.055 | -1.25 | | 217058_at | GNAS | GNAS complex locus | 3.82E-04 | 0.056 | -1.25 | | 203379_at | RPS6KA1 | ribosomal protein S6 kinase, 90kDa, polypeptide 1 | 3.93E-04 | 0.056 | -1.29 | | 228026_at | CSDE1 | cold shock domain containing E1, RNA-binding | 3.93E-04 | 0.056 | -1.43 | | 215949_x_at | IGHM | immunoglobulin heavy constant mu | 3.95E-04 | 0.056 | -1.42 | | 217696_at | FUT7 | fucosyltransferase 7 (alpha (1,3) fucosyltransferase) | 4.03E-04 | 0.056 | -1.36 | | 222265_at | TNS4 | tensin 4 | 4.04E-04 | 0.056 | -1.34 | | 207624_s_at | RPGR | retinitis pigmentosa GTPase regulator | 4.17E-04 | 0.057 | -1.26 | | 220734_s_at | LOC727825 | hypothetical LOC727825 | 4.22E-04 | 0.057 | -1.25 | | 209405_s_at | FAM3A | family with sequence similarity 3, member A | 4.32E-04 | 0.058 | -1.31 | | 201990_s_at | CREBL2 | cAMP responsive element binding protein-like 2 | 4.35E-04 | 0.058 | -1.23 | | 201407_s_at | PPP1CB | protein phosphatase 1, catalytic subunit, beta isoform | 4.47E-04 | 0.058 | -1.40 | | 227644_at | RIMS4 | regulating synaptic membrane exocytosis 4 | 4.47E-04 | 0.058 | -1.28 | | 229142_s_at | BBS1 | Bardet-Biedl syndrome 1 | 4.53E-04 | 0.058 | -1.32 | | 229727_x_at | FAM148B | family with sequence similarity 148, member B | 4.53E-04 | 0.058 | -1.47 | | 224501_at | C1orf170 | chromosome 1 open reading frame 170 | 4.62E-04 | 0.059 | -1.25 | | 230576_at | BLOC1S3 | biogenesis of lysosome-related organelles complex-1, subunit 3 | 4.91E-04 | 0.060 | -1.29 | | 212101_at | KPNA6 | karyopherin alpha 6 (importin alpha 7) | 4.96E-04 | 0.060 | -1.26 | | 242402_x_at | CENTD1 | centaurin, delta 1 | 5.22E-04 | 0.061 | -1.52 | | 58994_at | CC2D1A | coiled-coil and C2 domain containing 1A | 5.23E-04 | 0.061 | -1.27 | | 204857_at | MAD1L1 | MAD1 mitotic arrest deficient-like 1 (yeast) | 5.36E-04 | 0.061 | -1.24 | | 227095_at | LEPR | leptin receptor | 5.42E-04 | 0.061 | -1.32 | | 243449_at | OSBP2 | oxysterol binding protein 2 | 5.44E-04 | 0.061 | -1.31 | | 213447_at | SNRPN | small nuclear ribonucleoprotein polypeptide N | 5.73E-04 | 0.061 | -1.23 | | 227820_at | TBC1D25 | TBC1 domain family, member 25 | 5.84E-04 | 0.061 | -1.32 | | 221921_s_at | CADM3 | cell adhesion molecule 3 | 5.90E-04 | 0.061 | -1.31 | | 209643_s_at | PLD2 | phospholipase D2 | 6.00E-04 | 0.061 | -1.22 | | 207845_s_at | ANAPC10 | anaphase promoting complex subunit 10 | 6.09E-04 | 0.061 | -1.23 | | 206813_at | CTF1 | cardiotrophin 1 | 6.11E-04 | 0.061 | -1.50 | | 209472_at | CCBL2 | cysteine conjugate-beta lyase 2 | 6.14E-04 | 0.061 | -1.20 | | 205516_x_at | CIZ1 | CDKN1A interacting zinc finger protein 1 | 6.19E-04 | 0.061 | -1.24 | | 212019_at | RSL1D1 | ribosomal L1 domain containing 1 | 6.42E-04 | 0.062 | -1.38 | | 209111_at | RNF5 | ring finger protein 5 | 6.51E-04 | 0.062 | -1.20 | | 208924_at | RNF11 | ring finger protein 11 | 6.68E-04 | 0.062 | -1.26 | | 214658_at | TICAM2 | toll-like receptor adaptor molecule 2 | 6.69E-04 | 0.062 | -1.32 | | 217976_s_at | DYNC1LI1 | dynein, cytoplasmic 1, light intermediate chain 1 | 6.69E-04 | 0.062 | -1.56 | | 213296_at | RER1 | RER1 retention in endoplasmic reticulum 1 homolog (S. cerevisiae) | 6.75E-04 | 0.062 | -1.22 | | 220788_s_at | RNF31 | ring finger protein 31 | 6.85E-04 | 0.063 | -1.26 | | 222235_s_at | CSGALNACT2 | chondroitin sulfate N-acetylgalactosaminyltransferase 2 | 6.94E-04 | 0.063 | -1.36 | | 226704_at | UBE2J2 | ubiquitin-conjugating enzyme E2, J2 (UBC6 homolog, yeast) | 6.97E-04 | 0.063 | -1.23 | | 233337_s_at | SEZ6L2 | seizure related 6 homolog (mouse)-like 2 | 6.98E-04 | 0.063 | -1.19 | | 218255_s_at | FBRS | fibrosin | 7.15E-04 | 0.064 | -1.25 | | 208874_x_at | PPP2R4 | protein phosphatase 2A activator, regulatory subunit 4 | 7.37E-04 | 0.064 | -1.36 | | 226673_at | SH2D3C | SH2 domain containing 3C | 7.39E-04 | 0.064 | -1.27 | | 234256_at | SEBOX | SEBOX homeobox | 7.48E-04 | 0.064 | -1.25 | | 208946_s_at | BECN1 | beclin 1, autophagy related | 7.95E-04 | 0.066 | -1.22 | | 208660_at | CS | citrate synthase | 7.97E-04 | 0.066 | -1.18 | | 234918_at | GLTSCR2 | glioma tumor suppressor candidate region gene 2 | 8.03E-04 | 0.066 | -1.31 | | 231850_x_at | KIAA1712 | KIAA1712 | 8.04E-04 | 0.066 | -1.34 | | 214630_at | CYP11B2 | cytochrome P450, family 11, subfamily B, polypeptide 2 | 8.12E-04 | 0.066 | -1.40 | | 227203_at | FBXL17 | F-box and leucine-rich repeat protein 17 | 8.15E-04 | 0.066 | -1.25 | | 201321_s_at | SMARCC2 | SWI/SNF related, matrix associated, actin dependent regulator of chromatin, subfamily c, member 2 | 8.19E-04 | 0.066 | -1.21 | | 221988_at | MED26 | mediator complex subunit 26 | 8.20E-04 | 0.066 | -1.19 | | 243766_s_at | TEAD2 | TEA domain family member 2 | 8.25E-04 | 0.066 | -1.31 | | 203842_s_at | MAPRE3 | microtubule-associated protein, RP/EB family, member 3 | 8.32E-04 | 0.066 | -1.38 | | 219972_s_at | C14orf135 | chromosome 14 open reading frame 135 | 8.72E-04 | 0.068 | -1.37 | | 240886_at | CASR | calcium-sensing receptor (hypocalciuric hypercalcemia 1, severe neonatal hyperparathyroidism) | 9.03E-04 | 0.069 | -1.27 | | 219952_s_at | MCOLN1 | mucolipin 1 | 9.21E-04 | 0.069 | -1.27 | | 230780_at | LOC730091 | hypothetical protein LOC730091 | 9.23E-04 | 0.069 | -1.36 | | 239137_x_at | C6orf223 | chromosome 6 open reading frame 223 | 9.29E-04 | 0.069 | -1.26 | | 213318_s_at | BAT3 | HLA-B associated transcript 3 | 9.30E-04 | 0.069 | -1.23 | | 208405_s_at | CD164 | CD164 molecule, sialomucin | 9.37E-04 | 0.069 | -1.28 | | 201193_at | IDH1 | isocitrate dehydrogenase 1 (NADP+), soluble | 9.41E-04 | 0.069 | -1.36 | | 204569_at | ICK | intestinal cell (MAK-like) kinase | 9.62E-04 | 0.070 | -1.30 | | 219908_at | DKK2 | dickkopf homolog 2 (Xenopus laevis) | 9.62E-04 | 0.070 | -1.38 | | 212705_x_at | PNPLA2 | patatin-like phospholipase domain containing 2 | 9.99E-04 | 0.071 | -1.36 | | 218720_x_at | SEZ6L2 | seizure related 6 homolog (mouse)-like 2 | 1.02E-03 | 0.071 | -1.21 | | 231835_at | C1orf93 | chromosome 1 open reading frame 93 | 1.03E-03 | 0.071 | -1.17 | | 201139_s_at | SSB | Sjogren syndrome antigen B (autoantigen La) | 1.03E-03 | 0.071 | -1.30 | | 217916_s_at | FAM49B | family with sequence similarity 49, member B | 1.04E-03 | 0.071 | -1.21 | | 230228_at | LOC284297 | hypothetical LOC284297 | 1.04E-03 | 0.071 | -1.33 | | 235433_at | APOOL | apolipoprotein O-like | 1.06E-03 | 0.072 | -1.22 | | 215045_at | TNRC4 | trinucleotide repeat containing 4 | 1.08E-03 | 0.073 | -1.24 | | 222604_at | GTF3C3 | general transcription factor IIIC, polypeptide 3, 102kDa | 1.12E-03 | 0.075 | -1.31 | | 224914_s_at | DNAJC14 | DnaJ (Hsp40) homolog, subfamily C, member 14 | 1.13E-03 | 0.075 | -1.18 | | 33322_i_at | SFN | stratifin | 1.14E-03 | 0.076 | -1.35 | | 209769_s_at | GP1BB | glycoprotein Ib (platelet), beta polypeptide | 1.14E-03 | 0.076 | -1.45 | | 202778_s_at | ZMYM2 | zinc finger, MYM-type 2 | 1.15E-03 | 0.076 | -1.19 | | 204945_at | PTPRN | protein tyrosine phosphatase, receptor type, N | 1.16E-03 | 0.076 | -1.23 | | 213479_at | NPTX2 | neuronal pentraxin II | 1.17E-03 | 0.076 | -1.37 | | 228792_at | SLC24A5 | solute carrier family 24, member 5 | 1.18E-03 | 0.076 | -1.24 | | 209595_at | GTF2F2 | general transcription factor IIF, polypeptide 2, 30kDa | 1.18E-03 | 0.076 | -1.27 | | 204159_at | CDKN2C | cyclin-dependent kinase inhibitor 2C (p18, inhibits CDK4) | 1.20E-03 | 0.077 | -1.23 | | 220465_at | FLJ12355 | hypothetical protein FLJ12355 | 1.20E-03 | 0.077 | -1.35 | | 215038_s_at | SETD2 | SET domain containing 2 | 1.21E-03 | 0.077 | -1.31 | | 234950_s_at | RFWD2 | ring finger and WD repeat domain 2 | 1.24E-03 | 0.078 | -1.23 | | 205125_at | PLCD1 | phospholipase C, delta 1 | 1.27E-03 | 0.078 | -1.16 | | 218911_at | YEATS4 | YEATS domain containing 4 | 1.28E-03 | 0.079 | -1.32 | | 211837_s_at | PTCRA | pre T-cell antigen receptor alpha | 1.34E-03 | 0.079 | -1.32 | | 212210_at | INTS1 | integrator complex subunit 1 | 1.34E-03 | 0.079 | -1.30 | | 209490_s_at | PPT2 | palmitoyl-protein thioesterase 2 | 1.35E-03 | 0.079 | -1.20 | | 206410_at | NR0B2 | nuclear receptor subfamily 0, group B, member 2 | 1.35E-03 | 0.079 | -1.24 | | 215857_at | NCLN | nicalin homolog (zebrafish) | 1.36E-03 | 0.079 | -1.27 | | 218147_s_at | GLT8D1 | glycosyltransferase 8 domain containing 1 | 1.36E-03 | 0.079 | -1.25 | | 218596_at | TBC1D13 | TBC1 domain family, member 13 | 1.37E-03 | 0.079 | -1.22 | | 213149_at | DLAT | dihydrolipoamide S-acetyltransferase | 1.37E-03 | 0.079 | -1.40 | | 1555037_a_at | IDH1 | isocitrate dehydrogenase 1 (NADP+), soluble | 1.37E-03 | 0.079 | -1.26 | | 226372_at | CHST11 | carbohydrate (chondroitin 4) sulfotransferase 11 | 1.38E-03 | 0.079 | -1.39 | | 54037_at | HPS4 | Hermansky-Pudlak syndrome 4 | 1.39E-03 | 0.079 | -1.20 | | 224615_x_at | HM13 | histocompatibility (minor) 13 | 1.41E-03 | 0.079 | -1.20 | | 211698_at | EID1 | EP300 interacting inhibitor of differentiation 1 | 1.41E-03 | 0.079 | -1.40 | | 225125_at | TMEM32 | transmembrane protein 32 | 1.41E-03 | 0.079 | -1.20 | | 202634_at | POLR2K | polymerase (RNA) II (DNA directed) polypeptide K, 7.0kDa | 1.42E-03 | 0.079 | -1.20 | | 226379_s_at | C19orf25 | chromosome 19 open reading frame 25 | 1.42E-03 | 0.079 | -1.22 | | 218099_at | TEX2 | testis expressed 2 | 1.45E-03 | 0.080 | -1.20 | | 202312_s_at | COL1A1 | collagen, type I, alpha 1 | 1.45E-03 | 0.080 | -1.30 | | 211979_at | GPR107 | G protein-coupled receptor 107 | 1.46E-03 | 0.080 | -1.21 | | 226686_at | CISD2 | CDGSH iron sulfur domain 2 | 1.47E-03 | 0.080 | -1.24 | | 218311_at | MAP4K3 | mitogen-activated protein kinase kinase kinase kinase 3 | 1.47E-03 | 0.080 | -1.27 | | 63009_at | SHQ1 | SHQ1 homolog (S. cerevisiae) | 1.47E-03 | 0.080 | -1.21 | | 201561_s_at | CLSTN1 | calsyntenin 1 | 1.48E-03 | 0.080 | -1.26 | | 222768_s_at | TRMT6 | tRNA methyltransferase 6 homolog (S. cerevisiae) | 1.48E-03 | 0.080 | -1.20 | | 224891_at | FOXO3 | forkhead box O3 | 1.51E-03 | 0.081 | -1.30 | | 236737_at | C17orf56 | chromosome 17 open reading frame 56 | 1.52E-03 | 0.081 | -1.24 | | 213360_s_at | POM121C | POM121 membrane glycoprotein C | 1.52E-03 | 0.081 | -1.18 | | 223695_s_at | ARSD | arylsulfatase D | 1.54E-03 | 0.082 | -1.35 | | 226074_at | PPM1M | protein phosphatase 1M (PP2C domain containing) | 1.56E-03 | 0.082 | -1.19 | | 218487_at | ALAD | aminolevulinate, delta-, dehydratase | 1.56E-03 | 0.082 | -1.26 | | 1554202_x_at | CABP4 | calcium binding protein 4 | 1.57E-03 | 0.082 | -1.21 | | 226492_at | SEMA6D | sema domain, transmembrane domain (TM), and cytoplasmic domain, (semaphorin) 6D | 1.57E-03 | 0.082 | -1.64 | | 215096_s_at | ESD | esterase D/formylglutathione hydrolase | 1.57E-03 | 0.082 | -1.18 | | 1552789_at | TLOC1 | translocation protein 1 | 1.58E-03 | 0.082 | -1.29 | | 212876_at | B4GALT4 | UDP-Gal:betaGlcNAc beta 1,4- galactosyltransferase, polypeptide 4 | 1.58E-03 | 0.082 | -1.28 | | 200643_at | HDLBP | high density lipoprotein binding protein (vigilin) | 1.59E-03 | 0.082 | -1.35 | | 211358_s_at | CIZ1 | CDKN1A interacting zinc finger protein 1 | 1.60E-03 | 0.083 | -1.19 | | 1566177_at | CLYBL | citrate lyase beta like | 1.60E-03 | 0.083 | -1.25 | | 209523_at | TAF2 | TAF2 RNA polymerase II, TATA box binding protein (TBP)-associated factor, 150kDa | 1.63E-03 | 0.083 | -1.26 | | 208788_at | ELOVL5 | ELOVL family member 5, elongation of long chain fatty acids (FEN1/Elo2, SUR4/Elo3-like, yeast) | 1.63E-03 | 0.083 | -1.16 | | 223342_at | RRM2B | ribonucleotide reductase M2 B (TP53 inducible) | 1.64E-03 | 0.083 | -1.28 | | 230192_at | TRIM13 | tripartite motif-containing 13 | 1.65E-03 | 0.083 | -1.19 | | 229546_at | LOC653602 | hypothetical LOC653602 | 1.65E-03 | 0.083 | -1.33 | | 223084_s_at | CCNDBP1 | cyclin D-type binding-protein 1 | 1.66E-03 | 0.083 | -1.21 | | 215354_s_at | PELP1 | proline, glutamate and leucine rich protein 1 | 1.66E-03 | 0.083 | -1.23 | | 228876_at | BAIAP2L2 | BAI1-associated protein 2-like 2 | 1.67E-03 | 0.083 | -1.29 | | 225585_at | RAP2A | RAP2A, member of RAS oncogene family | 1.68E-03 | 0.083 | -1.28 | | 218196_at | OSTM1 | osteopetrosis associated transmembrane protein 1 | 1.69E-03 | 0.083 | -1.18 | | 213105_s_at | C16orf42 | chromosome 16 open reading frame 42 | 1.69E-03 | 0.083 | -1.19 | | 213044_at | ROCK1 | Rho-associated, coiled-coil containing protein kinase 1 | 1.70E-03 | 0.083 | -1.25 | | 213885_at | TRIM3 | tripartite motif-containing 3 | 1.71E-03 | 0.083 | -1.19 | | 244183_x_at | PCDHB3 | protocadherin beta 3 | 1.71E-03 | 0.083 | -1.23 | | 216220_s_at | ADORA1 | adenosine A1 receptor | 1.74E-03 | 0.084 | -1.30 | | 216105_x_at | PPP2R4 | protein phosphatase 2A activator, regulatory subunit 4 | 1.74E-03 | 0.084 | -1.31 | | 202806_at | DBN1 | drebrin 1 | 1.75E-03 | 0.084 | -1.15 | | 220536_at | C14orf115 | chromosome 14 open reading frame 115 | 1.77E-03 | 0.085 | -1.26 | | 212780_at | SOS1 | son of sevenless homolog 1 (Drosophila) | 1.79E-03 | 0.085 | -1.17 | | 209021_x_at | KIAA0652 | KIAA0652 | 1.79E-03 | 0.085 | -1.23 | | 202366_at | ACADS | acyl-Coenzyme A dehydrogenase, C-2 to C-3 short chain | 1.79E-03 | 0.085 | -1.21 | | 208868_s_at | GABARAPL1 | GABA(A) receptor-associated protein like 1 | 1.79E-03 | 0.085 | -1.25 | | 231852_at | THEX1 | three prime histone mRNA exonuclease 1 | 1.81E-03 | 0.085 | -1.76 | | 212507_at | TMEM131 | transmembrane protein 131 | 1.82E-03 | 0.085 | -1.23 | | 201701_s_at | PGRMC2 | progesterone receptor membrane component 2 | 1.83E-03 | 0.085 | -1.26 | | 219487_at | BBS10 | Bardet-Biedl syndrome 10 | 1.83E-03 | 0.085 | -1.31 | | 201091_s_at | CBX3 | chromobox homolog 3 (HP1 gamma homolog, Drosophila) | 1.84E-03 | 0.085 | -1.24 | | 224975_at | NFIA | nuclear factor I/A | 1.85E-03 | 0.085 | -1.67 | | 224789_at | WDR40A | WD repeat domain 40A | 1.85E-03 | 0.085 | -1.19 | | 212484_at | FAM89B | family with sequence similarity 89, member B | 1.85E-03 | 0.085 | -1.24 | | 200948_at | MLF2 | myeloid leukemia factor 2 | 1.86E-03 | 0.085 | -1.18 | | 225813_at | RC3H2 | ring finger and CCCH-type zinc finger domains 2 | 1.86E-03 | 0.085 | -1.20 | | 1560475_at | LOC100129455 | hypothetical protein LOC100129455 | 1.87E-03 | 0.085 | -1.50 | | 209176_at | SEC23IP | SEC23 interacting protein | 1.87E-03 | 0.085 | -1.20 | | 229878_at | KIAA1731 | KIAA1731 | 1.89E-03 | 0.085 | -1.27 | | 225900_at | EXOC6B | exocyst complex component 6B | 1.89E-03 | 0.085 | -1.56 | | 229032_at | WSCD2 | WSC domain containing 2 | 1.92E-03 | 0.085 | -1.31 | | 206267_s_at | MATK | megakaryocyte-associated tyrosine kinase | 1.96E-03 | 0.086 | -1.33 | | 210758_at | PSIP1 | PC4 and SFRS1 interacting protein 1 | 1.96E-03 | 0.086 | -1.16 | | 215596_s_at | ZNF294 | zinc finger protein 294 | 1.97E-03 | 0.086 | -1.34 | | 214656_x_at | MYO1C | myosin IC | 1.97E-03 | 0.086 | -1.19 | | 35179_at | B3GAT3 | beta-1,3-glucuronyltransferase 3 (glucuronosyltransferase I) | 2.01E-03 | 0.087 | -1.22 | | 228397_at | TUG1 | taurine upregulated gene 1 | 2.03E-03 | 0.087 | -1.27 | | 201047_x_at | RAB6A | RAB6A, member RAS oncogene family | 2.03E-03 | 0.087 | -1.19 | | 217931_at | CNPY3 | canopy 3 homolog (zebrafish) | 2.06E-03 | 0.088 | -1.21 | | 201025_at | EIF5B | eukaryotic translation initiation factor 5B | 2.06E-03 | 0.088 | -1.20 | | 218522_s_at | MAP1S | microtubule-associated protein 1S | 2.07E-03 | 0.088 | -1.29 | | 226976_at | KPNA6 | karyopherin alpha 6 (importin alpha 7) | 2.07E-03 | 0.088 | -1.23 | | 237855_at | ZNF777 | zinc finger protein 777 | 2.08E-03 | 0.088 | -1.21 | | 242565_x_at | C21orf57 | chromosome 21 open reading frame 57 | 2.08E-03 | 0.088 | -1.27 | | 221226_s_at | ACCN4 | amiloride-sensitive cation channel 4, pituitary | 2.09E-03 | 0.088 | -1.46 | | 231836_at | HKR1 | GLI-Kruppel family member HKR1 | 2.10E-03 | 0.088 | -1.24 | | 226045_at | FRS2 | fibroblast growth factor receptor substrate 2 | 2.10E-03 | 0.088 | -1.23 | | 226053_at | MAP2K7 | mitogen-activated protein kinase kinase 7 | 2.10E-03 | 0.088 | -1.26 | | 37831_at | SIPA1L3 | signal-induced proliferation-associated 1 like 3 | 2.11E-03 | 0.088 | -1.23 | | 222861_x_at | FBXO2 | F-box protein 2 | 2.11E-03 | 0.088 | -1.24 | | 218981_at | ACN9 | ACN9 homolog (S. cerevisiae) | 2.12E-03 | 0.088 | -1.42 | | 201455_s_at | NPEPPS | aminopeptidase puromycin sensitive | 2.15E-03 | 0.089 | -1.22 | | 216490_x_at | LOC442175 | similar to hCG1811681 | 2.15E-03 | 0.089 | -1.21 | | 1554175_at | CD300LB | CD300 molecule-like family member b | 2.17E-03 | 0.089 | -1.26 | | 228677_s_at | FLJ21438 | hypothetical protein FLJ21438 | 2.17E-03 | 0.089 | -1.32 | | 222010_at | ACAT2 | acetyl-Coenzyme A acetyltransferase 2 | 2.18E-03 | 0.089 | -1.17 | | 1552811_at | WFIKKN1 | WAP, follistatin/kazal, immunoglobulin, kunitz and netrin domain containing 1 | 2.19E-03 | 0.089 | -1.19 | | 243309_at | FLJ27352 | hypothetical LOC145788 | 2.20E-03 | 0.089 | -1.20 | | 213530_at | RAB3GAP1 | RAB3 GTPase activating protein subunit 1 (catalytic) | 2.21E-03 | 0.089 | -1.22 | | 219852_s_at | MORN1 | MORN repeat containing 1 | 2.22E-03 | 0.089 | -1.30 | | 243463_s_at | RIT1 | Ras-like without CAAX 1 | 2.23E-03 | 0.089 | -1.17 | | 202720_at | TES | testis derived transcript (3 LIM domains) | 2.23E-03 | 0.089 | -1.23 | | 227621_at | WTAP | Wilms tumor 1 associated protein | 2.24E-03 | 0.089 | -1.30 | | 1568986_x_at | PIGT | phosphatidylinositol glycan anchor biosynthesis, class T | 2.24E-03 | 0.090 | -1.30 | | 209717_at | EVI5 | ecotropic viral integration site 5 | 2.27E-03 | 0.090 | -1.20 | | 203846_at | TRIM32 | tripartite motif-containing 32 | 2.27E-03 | 0.090 | -1.17 | | 209874_x_at | CNNM2 | cyclin M2 | 2.27E-03 | 0.090 | -1.54 | | 205517_at | GATA4 | GATA binding protein 4 | 2.28E-03 | 0.090 | -1.29 | | 204660_at | GFER | growth factor, augmenter of liver regeneration (ERV1 homolog, S. cerevisiae) | 2.28E-03 | 0.090 | -1.34 | | 243539_at | KIAA1841 | KIAA1841 | 2.28E-03 | 0.090 | -1.18 | | 208336_s_at | GPSN2 | glycoprotein, synaptic 2 | 2.31E-03 | 0.091 | -1.22 | | 217749_at | COPG | coatomer protein complex, subunit gamma | 2.31E-03 | 0.091 | -1.16 | | 219096_at | ARMC7 | armadillo repeat containing 7 | 2.31E-03 | 0.091 | -1.20 | | 239246_at | FARP1 | FERM, RhoGEF (ARHGEF) and pleckstrin domain protein 1 (chondrocyte-derived) | 2.32E-03 | 0.091 | -1.18 | | 236749_at | MNT | MAX binding protein | 2.35E-03 | 0.091 | -1.24 | | 211240_x_at | CTNND1 | catenin (cadherin-associated protein), delta 1 | 2.35E-03 | 0.091 | -1.20 | | 234846_at | DKFZp761P0212 | hypothetical protein DKFZp761P0212 | 2.38E-03 | 0.092 | -1.27 | | 217937_s_at | HDAC7 | histone deacetylase 7 | 2.39E-03 | 0.092 | -1.26 | | 1558530_at | LRTM2 | leucine-rich repeats and transmembrane domains 2 | 2.40E-03 | 0.092 | -1.29 | | 222499_at | MRPS16 | mitochondrial ribosomal protein S16 | 2.42E-03 | 0.092 | -1.22 | | 214594_x_at | ATP8B1 | ATPase, class I, type 8B, member 1 | 2.42E-03 | 0.092 | -1.58 | | 202809_s_at | INTS3 | integrator complex subunit 3 | 2.42E-03 | 0.092 | -1.17 | | 226222_at | KIAA1432 | KIAA1432 | 2.42E-03 | 0.092 | -1.21 | | 212212_s_at | INTS1 | integrator complex subunit 1 | 2.44E-03 | 0.093 | -1.18 | | 203871_at | SENP3 | SUMO1/sentrin/SMT3 specific peptidase 3 | 2.47E-03 | 0.094 | -1.16 | | 203460_s_at | PSEN1 | presenilin 1 (Alzheimer disease 3) | 2.48E-03 | 0.094 | -1.20 | | 200945_s_at | SEC31A | SEC31 homolog A (S. cerevisiae) | 2.50E-03 | 0.095 | -1.16 | | 220798_x_at | PRG2 | plasticity-related gene 2 | 2.52E-03 | 0.095 | -1.21 | | 1560116_a_at | NEDD1 | neural precursor cell expressed, developmentally down-regulated 1 | 2.53E-03 | 0.095 | -1.23 | | 202962_at | KIF13B | kinesin family member 13B | 2.53E-03 | 0.095 | -1.20 | | 213500_at | COPB2 | coatomer protein complex, subunit beta 2 (beta prime) | 2.53E-03 | 0.095 | -1.18 | | 1570241_at | SPATA21 | spermatogenesis associated 21 | 2.54E-03 | 0.095 | -1.29 | | 201620_at | MBTPS1 | membrane-bound transcription factor peptidase, site 1 | 2.59E-03 | 0.096 | -1.18 | | 224659_at | SEPN1 | selenoprotein N, 1 | 2.60E-03 | 0.096 | -1.22 | | 223012_at | UBXD1 | UBX domain containing 1 | 2.61E-03 | 0.096 | -1.16 | | 214077_x_at | MEIS3P1 | Meis homeobox 3 pseudogene 1 | 2.61E-03 | 0.096 | -1.23 | | 235047_x_at | BTBD14B | BTB (POZ) domain containing 14B | 2.63E-03 | 0.096 | -1.29 | | 218171_at | VPS4B | vacuolar protein sorting 4 homolog B (S. cerevisiae) | 2.64E-03 | 0.096 | -1.16 | | 227546_x_at | CCNL2 | cyclin L2 | 2.64E-03 | 0.096 | -1.24 | | 228084_at | PLA2G12A | phospholipase A2, group XIIA | 2.65E-03 | 0.096 | -1.21 | | 205165_at | CELSR3 | cadherin, EGF LAG seven-pass G-type receptor 3 (flamingo homolog, Drosophila) | 2.67E-03 | 0.096 | -1.24 | | 209610_s_at | SLC1A4 | solute carrier family 1 (glutamate/neutral amino acid transporter), member 4 | 2.67E-03 | 0.096 | -1.21 | | 215942_s_at | GTSE1 | G-2 and S-phase expressed 1 | 2.69E-03 | 0.097 | -1.26 | | 204525_at | PHF14 | PHD finger protein 14 | 2.72E-03 | 0.097 | -1.28 | | 208141_s_at | DOHH | deoxyhypusine hydroxylase/monooxygenase | 2.73E-03 | 0.097 | -1.22 | | 203419_at | MLL4 | myeloid/lymphoid or mixed-lineage leukemia 4 | 2.74E-03 | 0.097 | -1.22 | | 212782_x_at | POLR2J | polymerase (RNA) II (DNA directed) polypeptide J, 13.3kDa | 2.74E-03 | 0.097 | -1.16 | | 226274_at | CLCN5 | chloride channel 5 (nephrolithiasis 2, X-linked, Dent disease) | 2.75E-03 | 0.097 | -1.18 | | 218114_at | GGA1 | golgi associated, gamma adaptin ear containing, ARF binding protein 1 | 2.77E-03 | 0.097 | -1.19 | | 202666_s_at | ACTL6A | actin-like 6A | 2.78E-03 | 0.097 | -1.22 | | 217040_x_at | SOX15 | SRY (sex determining region Y)-box 15 | 2.78E-03 | 0.097 | -1.46 | | 218446_s_at | FAM18B | family with sequence similarity 18, member B | 2.78E-03 | 0.097 | -1.27 | | 241357_at | MAPK15 | mitogen-activated protein kinase 15 | 2.80E-03 | 0.097 | -1.25 | | 227637_at | TFCP2 | transcription factor CP2 | 2.82E-03 | 0.097 | -1.23 | | 200902_at | SEP15 | 15 kDa selenoprotein | 2.82E-03 | 0.097 | -1.26 | | 203080_s_at | BAZ2B | bromodomain adjacent to zinc finger domain, 2B | 2.83E-03 | 0.097 | -1.17 | | 238329_at | M-RIP | myosin phosphatase-Rho interacting protein | 2.83E-03 | 0.097 | -1.22 | | 201784_s_at | C11orf58 | chromosome 11 open reading frame 58 | 2.84E-03 | 0.097 | -1.16 | | 212567_s_at | MAP4 | microtubule-associated protein 4 | 2.84E-03 | 0.097 | -1.18 | | 202359_s_at | SNX19 | sorting nexin 19 | 2.85E-03 | 0.097 | -1.22 | | 211142_x_at | HLA-DOA | major histocompatibility complex, class II, DO alpha | 2.88E-03 | 0.098 | -1.20 | | 31799_at | COPB2 | coatomer protein complex, subunit beta 2 (beta prime) | 2.88E-03 | 0.098 | -1.15 | | 221626_at | ZNF506 | zinc finger protein 506 | 2.91E-03 | 0.098 | -1.21 | | 212146_at | PLEKHM2 | pleckstrin homology domain containing, family M (with RUN domain) member 2 | 2.91E-03 | 0.098 | -1.19 | | 235610_at | ALKBH8 | alkB, alkylation repair homolog 8 (E. coli) | 2.91E-03 | 0.098 | -1.52 | | 203389_at | KIF3C | kinesin family member 3C | 2.91E-03 | 0.098 | -1.17 | | 214590_s_at | UBE2D1 | ubiquitin-conjugating enzyme E2D 1 (UBC4/5 homolog, yeast) | 2.91E-03 | 0.098 | -1.27 | | 213251_at | SMARCA5 | SWI/SNF related, matrix associated, actin dependent regulator of chromatin, subfamily a, member 5 | 2.92E-03 | 0.098 | -1.19 | | 209250_at | DEGS1 | degenerative spermatocyte homolog 1, lipid desaturase (Drosophila) | 2.93E-03 | 0.098 | -1.15 | | 210283_x_at | PAIP1 | poly(A) binding protein interacting protein 1 | 2.93E-03 | 0.098 | -1.26 | | 218239_s_at | GTPBP4 | GTP binding protein 4 | 2.95E-03 | 0.098 | -1.18 | | 213348_at | CDKN1C | cyclin-dependent kinase inhibitor 1C (p57, Kip2) | 2.96E-03 | 0.098 | -1.26 | | 226434_at | C7orf47 | chromosome 7 open reading frame 47 | 2.97E-03 | 0.098 | -1.14 | | 202745_at | USP8 | ubiquitin specific peptidase 8 | 2.97E-03 | 0.098 | -1.18 | | 204355_at | DHX30 | DEAH (Asp-Glu-Ala-His) box polypeptide 30 | 2.98E-03 | 0.098 | -1.17 | | 211953_s_at | RANBP5 | RAN binding protein 5 | 2.98E-03 | 0.098 | -1.28 | | 221992_at | LOC440348 | similar to nuclear pore complex interacting protein | 2.99E-03 | 0.098 | -1.32 | | 226225_at | MCC | mutated in colorectal cancers | 3.01E-03 | 0.098 | -1.45 | | 226549_at | SBK1 | SH3-binding domain kinase 1 | 3.03E-03 | 0.098 | -1.27 | | 222236_s_at | DDEFL1 | development and differentiation enhancing factor-like 1 | 3.03E-03 | 0.098 | -1.18 | | 218152_at | HMG20A | high-mobility group 20A | 3.04E-03 | 0.098 | -1.17 | | 212449_s_at | LYPLA1 | lysophospholipase I | 3.05E-03 | 0.098 | -1.28 | | 219488_at | A4GALT | alpha 1,4-galactosyltransferase (globotriaosylceramide synthase) | 3.05E-03 | 0.098 | -1.19 | | 206169_x_at | ZC3H7B | zinc finger CCCH-type containing 7B | 3.09E-03 | 0.099 | -1.41 | | 213521_at | PTPN18 | protein tyrosine phosphatase, non-receptor type 18 (brain-derived) | 3.09E-03 | 0.099 | -1.44 | | 201745_at | TWF1 | twinfilin, actin-binding protein, homolog 1 (Drosophila) | 3.10E-03 | 0.099 | -1.19 | | 229091_s_at | CCNJ | cyclin J | 3.11E-03 | 0.099 | -1.21 | | 201519_at | TOMM70A | translocase of outer mitochondrial membrane 70 homolog A (S. cerevisiae) | 3.11E-03 | 0.099 | -1.25 | | 203563_at | AFAP1 | actin filament associated protein 1 | 3.13E-03 | 0.099 | -1.20 | |  |  |  |  |  |  | |  |
|  |  |  |
