# Supplementary material for: Transcriptomic and genetic studies identify NFAT5 as a candidate gene for cocaine dependence
Source: Transl Psychiatry. 2015 Oct 27;5(10):e667–. doi: 10.1038/tp.2015.158 (PMC4930134; doi:10.1038/tp.2015.158)
Supplement: Supplementary Table 4 [file tp2015158x11.doc]

| **Supplementary Table 4.** Predicted targets for mir-124a in the subset of up-regulated genes 6h after exposure of SH-SY5Y cells differentiated to dopaminergic neurons to cocaine 5 μM | | | | | | |
| --- | --- | --- | --- | --- | --- | --- |
|  | | | | | | |
| hsa_TGCCTTA,MIR-124A target site enrichment: C=535; O=22; E=9.46; R=2.32; rawP=0.0002; adjP=0.0015 | | | | | | |
|  | | | | | | |
|  | **User ID** | **Gene symbol** | **Gene Name** | **Entrez Gene** | **Ensembl** |  |
|  | 1553106_at | C5orf24 | chromosome 5 open reading frame 24 | 134553 | ENSG00000181904 |  |
|  | 1553107_s_at |  |
|  | 217560_at | GGA1 | golgi-associated, gamma adaptin ear containing, ARF binding protein 1 | 26088 | ENSG00000100083 |  |
|  | 214600_at | TEAD1 | TEA domain family member 1 (SV40 transcriptional enhancer factor) | 7003 | ENSG00000187079 |  |
|  | 222463_s_at | BACE1 | beta-site APP-cleaving enzyme 1 | 23621 | ENSG00000265969  ENSG00000186318 |  |
|  | 224335_s_at |  |
|  | 209127_s_at | SART3 | squamous cell carcinoma antigen recognized by T cells 3 | 9733 | ENSG00000075856 |  |
|  | 219158_s_at | NAA15 | N(alpha)-acetyltransferase 15, NatA auxiliary subunit | 80155 | ENSG00000164134 |  |
|  | 222508_s_at | ARGLU1 | arginine and glutamate rich 1 | 55082 | ENSG00000134884 |  |
|  | 1567213_at | PNN | pinin, desmosome associated protein | 5411 | ENSG00000100941 |  |
|  | 221734_at | PRRC1 | proline-rich coiled-coil 1 | 133619 | ENSG00000164244 |  |
|  | 239270_at | PLCXD3 | phosphatidylinositol-specific phospholipase C, X domain containing 3 | 345557 | ENSG00000182836 |  |
|  | 200907_s_at | PALLD | palladin, cytoskeletal associated protein | 23022 | ENSG00000129116 |  |
|  | 233559_s_at | WDFY1 | WD repeat and FYVE domain containing 1 | 57590 | ENSG00000085449 |  |
|  | 203532_x_at | CUL5 | cullin 5 | 8065 | ENSG00000166266 |  |
|  | 213957_s_at | CEP350 | centrosomal protein 350kDa | 9857 | ENSG00000135837 |  |
|  | 219393_s_at | AKT3 | v-akt murine thymoma viral oncogene homolog 3 (protein kinase B, gamma) | 10000 | ENSG00000117020 |  |
|  | 212307_s_at | OGT | O-linked N-acetylglucosamine (GlcNAc) transferase | 8473 | ENSG00000147162 |  |
|  | 230071_at | 40787 | septin 11 | 55752 | ENSG00000138758 |  |
|  | 221276_s_at | SYNC | syncoilin, intermediate filament protein | 81493 | ENSG00000162520 |  |
|  | 203589_s_at | TFDP2 | transcription factor Dp-2 (E2F dimerization partner 2) | 7029 | ENSG00000114126 |  |
|  | 223984_s_at | NUPL1 | nucleoporin like 1 | 9818 | ENSG00000139496 |  |
|  | 211085_s_at | STK4 | serine/threonine kinase 4 | 6789 | ENSG00000101109 |  |
|  | 206232_s_at | B4GALT6 | UDP-Gal:betaGlcNAc beta 1,4- galactosyltransferase, polypeptide 6 | 9331 | ENSG00000118276 |  |
|  |  |  |  |  |  |  |
