# Supplementary material for: Transcriptomic and genetic studies identify NFAT5 as a candidate gene for cocaine dependence
Source: Transl Psychiatry. 2015 Oct 27;5(10):e667–. doi: 10.1038/tp.2015.158 (PMC4930134; doi:10.1038/tp.2015.158)
Supplement: Supplementary Table 5 [file tp2015158x12.doc]

|  | **Supplementary Table 5.** Single-marker association study of 23 SNPs in 806 cocaine-dependent patients and 817 sex-matched unrelated controls | | | | | | | | | | | | | | | | | | |  |
| --- | --- | --- | --- | --- | --- | --- | --- | --- | --- | --- | --- | --- | --- | --- | --- | --- | --- | --- | --- | --- |
|  |  |  |  | **Genotypes** | | | | | | | | |  | **HWE** | |  | **Case-control association study** | | |  |
|  |  |  |  | **Controls N (%)** | | | |  | **Cases N (%)** | | | |  | **Controls** | **Cases** |  |  | | |  |
|  | **Locus** | **Marker** | **Predicted effect** | **11** | **12** | **22** | **Sum** |  | **11** | **12** | **22** | **Sum** | **MAF** | **p-value** | **p-value** |  | **p-value**^1^ | **Adj**  **p-value**^2^ | **(OR -95%CI)** |  |
|  |  |  |  |  |  |  |  |  |  |  |  |  |  |  |  |  |  |  |  |  |
|  | ***NFAT5*** | rs1437134 | miRNA binding | 278 (34.2) | 384 (47.3) | 150 (18.5) | 812 |  | 325 (40.5) | 370 (46.1) | 108 (13.4) | 803 | 0.393 | 0.388 | 0.879 |  | **0.00118**^&^ | **0.00031**^&^ | 1.27 (1.10-1.45)* |  |
|  |  | rs7359336 | miRNA binding | 278 (34.1) | 386 (47.3) | 152 (18.6) | 816 |  | 321 (40.2) | 369 (46.2) | 109 (13.6) | 799 | 0.395 | 0.389 | 0.879 |  | **0.00141**^&^ | **0.00035**^&^ | 1.25 (1.09-1.45)* |  |
|  |  | rs11641233 | miRNA binding | 516 (63.2) | 264 (32.3) | 37 (4.5) | 817 |  | 461 (57.5) | 286 (35.7) | 55 (6.9) | 802 | 0.227 | 0.670 | 0.254 |  | **0.00734** | **0.02599** | 1.25 (1.06-1.47) |  |
|  |  | rs6499244 | miRNA binding | 264 (32.4) | 390 (47.9) | 161 (19.8) | 815 |  | 293 (36.5) | 385 (48.0) | 124 (5.5) | 802 | 0.416 | 0.434 | 0.941 |  | **0.0156** | **0.00474** | 1.37 (1.03-1.37)* |  |
|  |  | rs12232410 | miRNA binding | 516 (63.3) | 262 (32.1) | 37 (4.5) | 815 |  | 463 (57.5) | 288 (35.8) | 54 (6.7) | 805 | 0.226 | 0.593 | 0.342 |  | **0.00749** | **0.0351** | 1.25 (1.06-1.47) |  |
|  |  | rs1043470 | miRNA binding | 590 (72.3) | 216 (26.5) | 10 (1.2) | 816 |  | 584 (73.0) | 197 (24.6) | 19 (2.4) | 800 | 0.146 | 0.048 | 0.575 |  | 0.853 | 0.242 | - |  |
|  |  |  |  |  |  |  |  |  |  |  |  |  |  |  |  |  |  |  |  |  |
|  | ***ELF1*** | rs10507488 | TFBS | 601 (73.7) | 190 (23.3) | 25 (3.1) | 816 |  | 588 (73.0) | 195 (24.2) | 22 (2.7) | 805 | 0.148 | 0.049 | 0.263 |  | 0.914 | 0.172 | - |  |
|  |  | rs10507487 | TFBS | 600 (73.6) | 191 (23.4) | 24 (2.9) | 815 |  | 588 (73.0) | 196 (24.3) | 22 (2.7) | 806 | 0.148 | 0.070 | 0.265 |  | 0.860 | 0.181 | - |  |
|  |  | rs9594470 | TFBS | 599 (73.9) | 187 (23.1) | 25 (3.1) | 811 |  | 587 (72.9) | 196 (24.3) | 22 (2.7) | 805 | 0.148 | 0.034 | 0.265 |  | 0.818 | 0.818 | - |  |
|  |  | rs7799 | Non synonymous | 413 (50.8) | 324 (39.9) | 76 (9.3) | 813 |  | 389 (48.4) | 338 (42.1) | 76 (9.5) | 803 | 0.299 | 0.309 | 0.868 |  | 0.448 | 0.160 | - |  |
|  |  | rs3764056 | Splicing | 394 (48.6) | 333 (41.1) | 84 (10.4) | 811 |  | 375 (46.8) | 347 (43.3) | 80 (10.0) | 802 | 0.312 | 0.285 | 1 |  | 0.662 | 0.494 | - |  |
|  |  | rs1056820 | Non synonymous | 394 (48.5) | 335 (41.2) | 84 (10.3) | 813 |  | 375 (46.7) | 348 (43.3) | 80 (10.0) | 803 | 0.313 | 0.324 | 1 |  | 0.671 | 0.499 | - |  |
|  |  | rs1056824 | miRNA | 383 (48.0) | 328 (41.1) | 87 (10.9) | 798 |  | 370 (46.5) | 347 (43.6) | 78 (9.8) | 795 | 0.315 | 0.189 | 0.870 |  | 0.913 | 0.536 | - |  |
|  |  | rs1056835 | miRNA | 403 (49.4) | 324 (39.8) | 88 (10.8) | 815 |  | 387 (48.2) | 341 (42.5) | 75 (9.3) | 803 | 0.306 | 0.070 | 1 |  | 0.951 | 0.596 | - |  |
|  |  | rs9532662 | TFBS | 403 (49.5) | 324 (39.8) | 87 (10.7) | 814 |  | 388 (48.6) | 335 (42.0) | 75 (9.4) | 798 | 0.305 | 0.083 | 0.867 |  | 0.903 | 0.683 | - |  |
|  |  |  |  |  |  |  |  |  |  |  |  |  |  |  |  |  |  |  |  |  |
|  | ***PPP1R9A*** | rs10953132 | TFBS | 244 (29.9) | 406 (49.8) | 165 (20.6) | 815 |  | 216 (27.0) | 420 (52.4) | 165 (20.6) | 801 | 0.460 | 0.888 | 0.156 |  | 0.336 | 0.373 | - |  |
|  |  | rs854524 | Benign (polyphen) | 289 (35.5) | 423 (52.0) | 102 (12.5) | 814 |  | 305 (37.9) | 377 (46.8) | 123 (15.3) | 805 | 0.386 | **0.006** | 0.711 |  | - | - | - |  |
|  |  | rs854542 | miRNA binding | 608 (75.0) | 188 (23.2) | 15 (1.8) | 811 |  | 571 (71.1) | 219 (27.3) | 13 (1.6) | 803 | 0.143 | 0.880 | 0.134 |  | 0.136 | 0.239 | - |  |
|  |  |  |  |  |  |  |  |  |  |  |  |  |  |  |  |  |  |  |  |  |
|  | ***SEMA6D*** | rs4775708 | None / SD GWAS positive | 438 (54.1) | 318 (39.3) | 54 (6.7) | 810 |  | 455 (57.3) | 280 (35.3) | 59 (7.4) | 794 | 0.257 | 0.786 | 0.089 |  | 0.429 | 0.759 | - |  |
|  |  | rs76739 | TFBS | 267 (33.2) | 392 (48.8) | 145 (18.0) | 804 |  | 280 (35.0) | 376 (46.9) | 145 (18.1) | 801 | 0.420 | 0.943 | 0.345 |  | 0.634 | 0.501 | - |  |
|  |  | rs3809485 | None / SD GWAS positive | 205 (25.2) | 405 (49.8) | 204 (25.1) | 814 |  | 211 (26.3) | 390 (48.7) | 200 (25.0) | 801 | 0.496 | 0.889 | 0.480 |  | 0.724 | 0.685 | - |  |
|  |  | rs532598 | Non synonymous | 338 (41.5) | 373 (45.8) | 103 (12.7) | 814 |  | 359 (44.8) | 332 (41.4) | 111 (13.8) | 802 | 0.351 | 1 | 0.019 |  | 0.549 | 0.856 | - |  |
|  |  |  |  |  |  |  |  |  |  |  |  |  |  |  |  |  |  |  |  |  |
|  | ***IGF2BP3*** | rs10950949 | TFBS | 291 (35.9) | 387 (47.8) | 132 (16.3) | 810 |  | 255 (31.9) | 391 (48.9) | 153 (19.1) | 799 | 0.419 | 0.884 | 0.886 |  | **0.0492** | 0.407 | - |  |
|  |  |  |  |  |  |  |  |  |  |  |  |  |  |  |  |  |  |  |  |  |

MAF: Minimum Allele Frequency; HWE: Hardy-Weinberg Equilibrium; TFBS, Transcription Factor Binding Site; SD GWAS positive, Substance Dependence Genome Wide Association Study, SNP identified associated by Drgon et al PLoS One. 2010 Jan 21;5(1):e8832. In bold: significant p-values; ^1^ Log-additive model; ^2^ Adjusted by age; *When OR<1, the inverted score is shown; ^&^ Overcome Bonferroni correction p<0.0022.
