# Supplementary material for: Transcriptomic and genetic studies identify NFAT5 as a candidate gene for cocaine dependence
Source: Transl Psychiatry. 2015 Oct 27;5(10):e667–. doi: 10.1038/tp.2015.158 (PMC4930134; doi:10.1038/tp.2015.158)
Supplement: Supplementary Table 6 [file tp2015158x13.doc]

| **Supplementary Table 6**. Study of the effect of *NFAT5* variants on volumes of 7 brain regions involved in addiction using genetic and neuroimaging data from the BIG sample ([http://www.cognomics.nl](http://www.cognomics.nl/)). | | | | | | | |
| --- | --- | --- | --- | --- | --- | --- | --- |
|  |  | |  |  |  |  |  |
|  |  |  | **Discovery sample**  **(1.5 Tesla) N=645** |  | **Replication sample**  **(1.5 Tesla) N=645** | **Whole sample**  **N=1300** |  |
|  | **Brain volumes** | **SNPs** | **P-value** |  | **P-value** | **P-value** |  |
|  |  |  |  |  |  |  |  |
|  | Prefrontal cortex | rs1437134A | 0.1569 |  | 0.9645 | 0.3013 |  |
|  |  | rs11641233T | 0.9895 |  | 0.1267 | 0.259 |  |
|  | Insula | rs1437134A | 0.6895 |  | 0.0536 | 0.1047 |  |
|  |  | rs11641233T | 0.4842 |  | 0.9033 | 0.6997 |  |
|  | Hippocampus | rs1437134A | 0.3414 |  | 0.023 | 0.3328 |  |
|  |  | rs11641233T | 0.0188 |  | 0.9202 | 0.1619 |  |
|  | Nucleus accumbens | rs1437134A | 0.7974 |  | 0.488 | 0.6487 |  |
|  |  | rs11641233T | 0.1451 |  | 0.5691 | 0.605 |  |
|  | Caudate nucleus | rs1437134A | 0.2306 |  | 0.2402 | 0.9764 |  |
|  |  | rs11641233T | 0.0022 |  | 0.9314 | 0.0709 |  |
|  | Putamen | rs1437134A | 0.4312 |  | 0.2099 | 0.7661 |  |
|  |  | rs11641233T | 0.0347 |  | 0.5935 | 0.0644 |  |
|  | Medial orbitofrontal cortex | rs1437134A | 0.923 |  | 0.2342 | 0.4889 |  |
|  |  | rs11641233T | 0.2424 |  | 0.8316 | 0.262 |  |
|  |  | |  |  |  |  |  |
